# FGFR blockade inhibits targeted therapy-tolerant persister in basal FGFR1- and FGF2-high cancers with driver oncogenes

**DOI:** 10.1038/s41698-023-00462-0

**Published:** 2023-10-25

**Authors:** Koh Furugaki, Takaaki Fujimura, Hayato Mizuta, Takuya Yoshimoto, Takashi Asakawa, Yasushi Yoshimura, Shigeki Yoshiura

**Affiliations:** 1 Product Research Department, Chugai Pharmaceutical Co., Ltd., 216 Totsuka-cho, Totsuka-ku, Kanagawa 244-8602 Japan; 2 Biometrics Department, Chugai Pharmaceutical Co., Ltd., 2-1-1 Nihonbashi-muromachi, Chuo-ku, Tokyo 103-8324 Japan

**Keywords:** Lung cancer, Target identification

## Abstract

Cancer cell resistance arises when tyrosine kinase inhibitor (TKI)-targeted therapies induce a drug-tolerant persister (DTP) state with growth via genetic aberrations, making DTP cells potential therapeutic targets. We screened an anti-cancer compound library and identified fibroblast growth factor receptor 1 (FGFR1) promoting alectinib-induced anaplastic lymphoma kinase (ALK) fusion-positive DTP cell’s survival. FGFR1 signaling promoted DTP cell survival generated from basal FGFR1- and fibroblast growth factor 2 (FGF2)-high protein expressing cells, following alectinib treatment, which is blocked by FGFR inhibition. The hazard ratio for progression-free survival of ALK-TKIs increased in patients with ALK fusion-positive non-small cell lung cancer with *FGFR1*- and *FGF2*-high mRNA expression at baseline. The combination of FGFR and targeted TKIs enhanced cell growth inhibition and apoptosis induction in basal FGFR1- and FGF2-high protein expressing cells with ALK-rearranged and epidermal growth factor receptor (EGFR)-mutated NSCLC, human epidermal growth factor receptor 2 (HER2)-amplified breast cancer, or v-raf murine sarcoma viral oncogene homolog B1 (BRAF)-mutated melanoma by preventing compensatory extracellular signal-regulated kinase (ERK) reactivation. These results suggest that a targeted TKI-induced DTP state results from an oncogenic switch from activated oncogenic driver signaling to the FGFR1 pathway in basal FGFR1- and FGF2-high expressing cancers and initial dual blockade of FGFR and driver oncogenes based on FGFR1 and FGF2 expression levels at baseline is a potent treatment strategy to prevent acquired drug resistance to targeted TKIs through DTP cells regardless of types of driver oncogenes.

## Introduction

Oncogene addiction is a well-established paradigm in which a single gain-of-function oncogene sustains the growth and survival of cancer cells; targeting oncogene addiction by protein or pathway inhibition has great potential for use in cancer therapies^1^. Clinically actionable targets and active inhibitors against targets in cancer cells have been demonstrated^2^, and small-molecule inhibitors have been approved by the US Food and Drug Administration (FDA) for use against tumor cells with addicted oncogenes, including HER2, ALK, BRAF, and EGFR^3^. However, despite the response to these inhibitors, resistant tumor cells develop due to on-target alterations, bypass alterations, or alterations that cause phenotypic changes, loss of target, and target dependency^4^.

One process by which these three resistance mechanisms are acquired is entry into a reversible slow proliferation state known as drug-tolerant persister (DTP) state by a small population of cancer cells;^5^ DTP cells evade cell death induced by targeted therapy long enough to acquire additional multiple resistance mechanisms^6^. A stepwise accumulation of genetic mutations has been observed in resistant tumors during sequential therapy with several ALK-TKIs^7,8^. Furthermore, both on- and off-target mechanisms have been identified after acquired resistance even in the same patient^9^.

Multiple resistance mechanisms may coexist in tumors, making it difficult to devise second-line therapies and increase treatment response. Thus, more efficacious targeted therapies should be implemented up-front to prevent the emergence of additional resistance mechanisms rather than individually selecting drugs corresponding to each mechanism. However, even at the early stage of targeted TKI monotherapy, there are potentially complex mechanisms conferring a DTP state^6^. Thus, combinatorial approaches may be effective in initial treatments, and it is essential to identify the mechanisms by which a targeted TKI-induced DTP state is acquired and select a suitable combination treatment that suppresses the generation of DTP cells to maximize expected outcomes. Despite the accumulation of evidence for the presence of DTP cells in vitro^6^, the mechanisms by which cancer cells modulate targeted TKI tolerance in DTP states remain poorly understood.

Therefore, we explore effective candidates that act synergistically with ALK-TKI by screening an anti-cancer compound library. Furthermore, we test oncogene-targeted TKIs with EGFR, HER2, and BRAF inhibitors using EGFR-mutant (EGFR+) NSCLC, HER2-amplified (HER2+) breast cancer (BC), and BRAF-mutant (BRAF+) melanoma cells^10^. To increase applicability to clinical practice, we select FDA approved agents for use in this study^3^.

## Results

### Compound screening identifies FGFR1 as a candidate that promotes alectinib-induced DTP cells

To find effective targets that promote cell survival against ALK inhibition in ALK + NSCLC cells, we generated DTP cells that survive targeted therapy through reversible and non-mutational mechanisms from ALK + NCI-H2228 cells following exposure to alectinib^11^. An anti-cancer compound library was screened in NCI-H2228 parental cells or DTP cells. We calculated the ratio of antiproliferative effect on DTP cells to parental cells to identify effective candidates specifically required for cell survival against alectinib treatment, and nine compounds showed ratios of less than 0.7 without suppression of parental cell growth (Supplementary Data 1 and Supplementary Fig. 1). To confirm the results of this screen, a cell proliferation assay was performed using these nine compounds to determine the IC_50_ values. Erdafitinib showed the strongest inhibitory effect on DTP cells as compared to parental cells, with IC_50_ values of 6.7 nM and 6136.4 nM in DTP and parental cells, respectively (Supplementary Fig. 2A, B). Erdafitinib is a pan-FGFR-TKI that inhibits all members of the FGFR family, including FGFR1, FGFR2, FGFR3, and FGFR4^12,13^. An analysis of mRNA expressions of these FGFR members in parental cells revealed the highest expression of *FGFR1*, which was greater than 1.00 transcript per million (TPM) (Supplementary Fig. 2C). Therefore, we examined whether FGFR1 contributed to cell survival in the presence of alectinib in NCI-H2228 cells.

### DTP cells escape ALK-TKI-induced cell death through activation of FGFR signaling

**Fig. 1:**
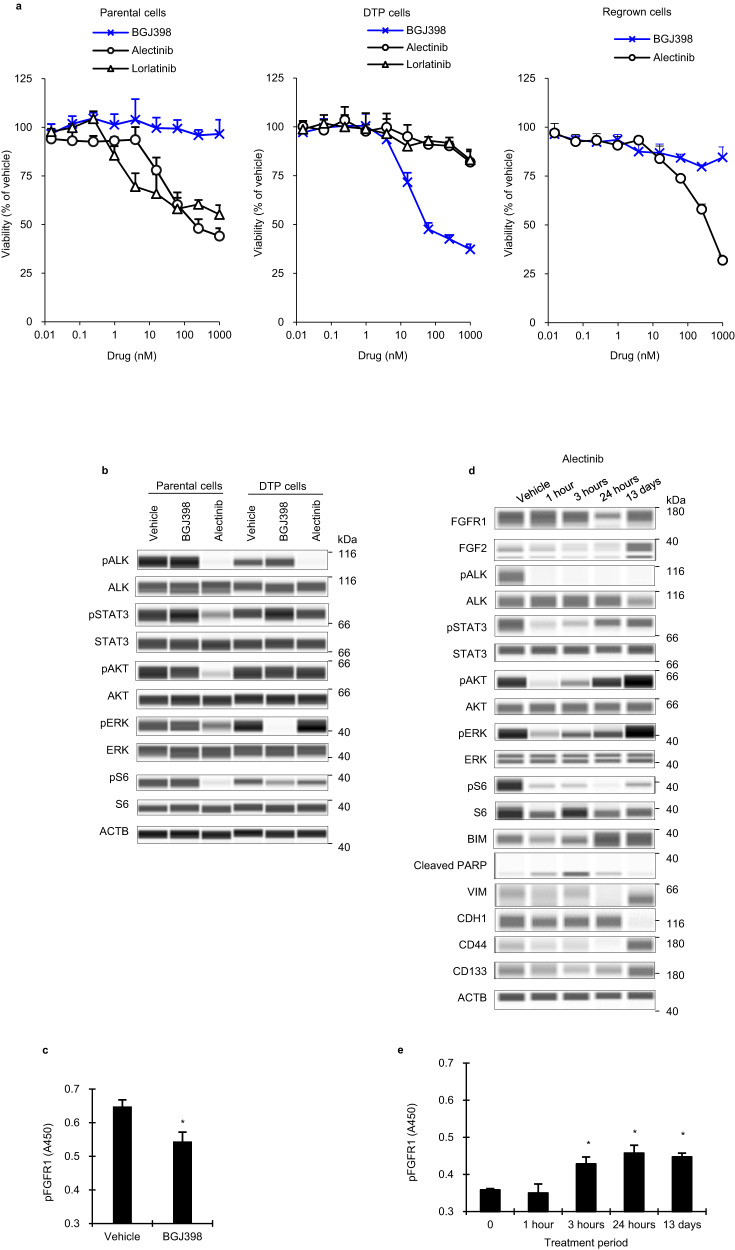
Effect of ALK or FGFR inhibitors on NCI-H2228 parental, DTP, and regrown cells. **a** Alectinib-DTP cells were generated from NCI-H2228 cells by treatment with 1000 nM alectinib for 13 days, and then the cells were cultured without alectinib for 37 days. Cells were cultured with alectinib, lorlatinib, or BGJ398 for 8 days. **b** Immunoblots of cell lysates of parental and alectinib-DTP cells treated with 1000 nM alectinib or 300 nM BGJ398 for 3 h. **c** FGFR1 phosphorylation levels of alectinib-DTP cells treated with 300 nM BGJ398 for 3 h measured by ELISA. Each point represents the mean + standard deviation of quadruplicate experiments. *P < 0.05 versus vehicle in the *t* test. **d** Immunoblots of cell lysates of parental cells were treated with 1000 nM alectinib for 1, 3, and 24 h, and 13 days. **e** FGFR1 phosphorylation levels of parental cells as described in Fig. 1d. Each point represents the mean + standard deviation of quadruplicate experiments. *P < 0.05 versus 0 h from Dunnett’s test.

To determine whether pharmacologic inhibition of FGFR1 sensitizes ALK + NCI-H2228 DTP cells, we assessed sensitivity to pan-FGFR-TKI (BGJ398). Parental cells were sensitive to ALK-TKIs (alectinib and lorlatinib) and insensitive to BGJ398 (IC_50_ values of 219.6 nM for alectinib and > 1000 nM for BGJ398; IC_30_ value of 3.8 nM for lorlatinib), whereas DTP cells were insensitive to ALK-TKIs and sensitive to BGJ398 (IC_50_ values of >1000 nM for alectinib and 57.8 nM for BGJ398; IC_30_ value of > 1000 nM for lorlatinib) (Fig. 1a, Supplementary Fig. 3A). Furthermore, although alectinib inhibited phosphorylation of ALK, as well as STAT3, AKT, ERK, and S6, which are involved in ALK or FGFR1 downstream signaling, in parental cells, it did not inhibit STAT3, AKT, ERK, and S6 phosphorylation in DTP cells regardless of complete suppression of ALK phosphorylation (Fig. 1b). BGJ398 did not inhibit phosphorylation of STAT3, AKT, ERK, and ALK in parental cells, whereas BGJ398 significantly inhibited FGFR1 phosphorylation and markedly inhibited ERK phosphorylation in DTP cells (Fig. 1b, c).

NCI-H2228 regrown cells, generated from DTP cells by culturing in the alectinib-free medium for 37 days, restored sensitivity to alectinib and insensitivity to BGJ398 compared with DTP cells (Fig. 1a, Supplementary Fig. 3A). The susceptibility of regrown cells was similar to that of parental cells, indicating that their reversibility is identical to that of DTP cells reported by Mikubo et al.^6^. Therefore, FGFR1 signaling may be essential for the survival of DTP cells upon the ALK blockade.

To examine the mechanism by which dependence on FGFR1 kinase is acquired in DTP cells, we analyzed the time course of ALK and FGFR1 signal transduction activation under alectinib treatment. FGF-1, 2, 3, 4, 5, 6, 10, 19, 20, 21, and 22 specifically bind to FGFR1 and activate signaling pathways involving AKT and ERK^12,14^. Analysis of the mRNA expression levels of these 11 FGFs in parental cells revealed that only *FGF2* was expressed at greater than 1.00 TPM (Supplementary Fig. 3B). Thus, we examined FGFR1 and FGF2 protein expression levels. We found that FGF2 protein levels and FGFR1 phosphorylation levels increased significantly 13 days after treatment with alectinib compared to those before treatment, whereas almost no difference in FGFR1 protein levels was observed (Fig. 1d, e). In addition, phosphorylation levels of STAT3, AKT, and ERK were elevated 13 days after treatment, despite the complete suppression of ALK phosphorylation during alectinib exposure (Fig. 1d). DTP cells reportedly have morphological alterations, stemness, or epithelial-mesenchymal transition (EMT), including vimentin (VIM) upregulation and cadherin 1 (CDH1) downregulation^6,15^. NCI-H2228-DTP cells also acquired these features (Fig. 1d, Supplementary Fig. 3C). BIM, a pro-apoptotic BCL2-family protein that mediates ALK inhibitor-induced apoptosis in ALK+lung cancer cells^16^, was increased from 24 h to 13 days after treatment, whereas the cleaved PARP protein, which indicates apoptosis, temporarily increased 3 h after treatment, but disappeared thereafter (Fig. 1d). These findings suggest that apoptosis was immediately induced in NCI-H2228 cells via suppression of ALK signaling 3 h after alectinib treatment, but cells survived by promoting a DTP state through the acquisition of stemness, EMT features, and activation of FGFR1 signaling, particularly downstream ERK reactivation, through increased FGF2 expression.

### FGFR1 and FGF2 expressions promote cell survival against ALK-TKI

**Fig. 2:**
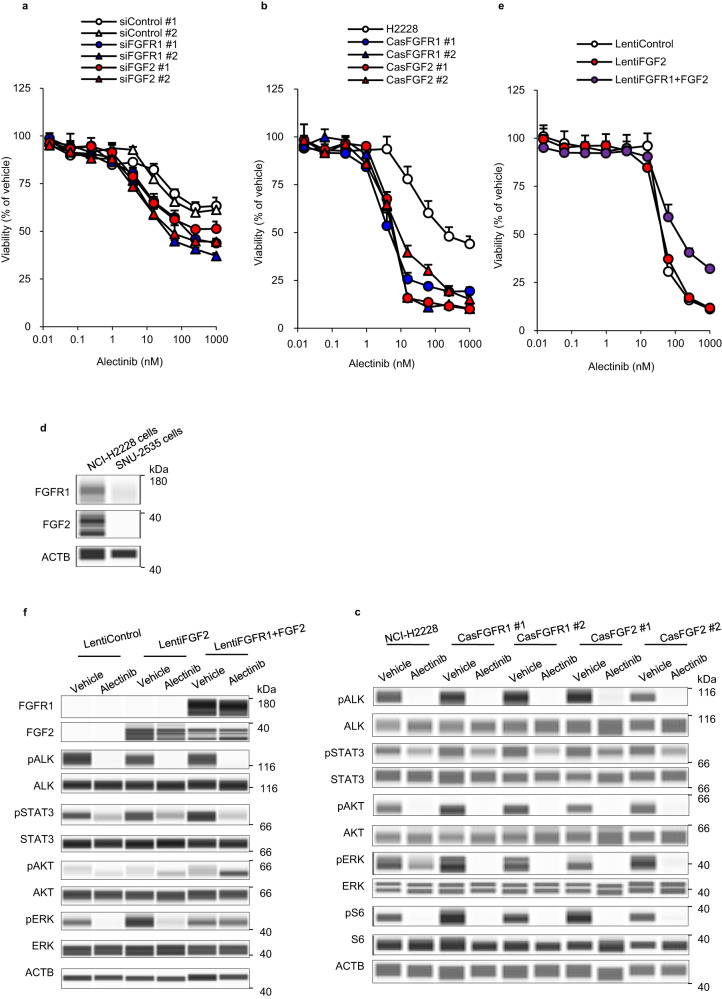
Effect of knockdown and knock-in of FGFR1 or FGF2 on sensitivity to alectinib. **a** NCI-H2228 cells transfected with two siRNAs against FGFR1, FGF2, or nontargeting control were cultured with alectinib for 6 days. **b** NCI-H2228 cells were transfected with Cas9 protein and crRNA against FGFR1 or FGF2. Two clones were isolated and cultured in the alectinib for 8 days. **c** Immunoblots of cell lysates of FGFR1 or FGF2-knockout clones treated with 1000 nM alectinib for 3 h. **d** Immunoblots of cell lysates of NCI-H2228 cells and SNU-2535 cells. **e** SNU-2535 cells were transfected with lentiviral overexpression vector of FGF2, FGFR1, and FGF2, or nontargeting control. Each clone was isolated and cultured with alectinib for 8 days. **f** Immunoblots of cell lysates of nontargeting control, FGF2-, or FGFR1- and FGF2-overexpressing clones treated with 1000 nM alectinib for 3 h.

To validate whether activation of FGFR1 signaling by elevated expression of the FGF2 protein is essential for cell survival against ALK-TKIs, we performed knockdowns of FGFR1 and FGF2 using small interfering RNA (siRNA) and CRISPR/Cas9 in NCI-H2228 cells. Alectinib sensitivity was increased in NCI-H2228 cells transfected with FGFR1 or FGF2 siRNA relative to control siRNAs, with IC_40_ values of > 1000 nM and 246.4 nM for control siRNAs, 47.0 nM and 14.7 nM for FGFR1 siRNAs, and 41.5 nM and 14.9 nM for FGF2 siRNAs (Fig. 2a, Supplementary Fig. 4A). In addition, alectinib sensitivity increased in both FGFR1- or FGF2-knockout clone transfected with FGFR1 or FGF2 crRNA relative to parental cells, with IC_50_ values of 5.4 nM and 7.3 nM for FGFR1-knockouts, 5.6 nM and 6.1 nM for FGF2-knockouts, and 219.6 nM for parental cells (Fig. 2b, Supplementary Fig. 4B). Alectinib-induced apoptosis and ERK inhibition were enhanced in both FGFR1- or FGF2-knockouts compared to those in parental cells (Fig. 2c, Supplementary Fig. 4C). FGFR1- or FGF2-knockout also enhanced sensitivity to lorlatinib, with IC_50_ values of 0.7 nM in both FGFR1-knockouts, 0.7 nM and 0.9 nM for FGF2-knockouts, and > 1000 nM for parental cells (Supplementary Fig. 4D).

To further verify that both FGFR1 and FGF2 are required for cell survival against ALK-TKIs, we established FGFR1- and FGF2-overexpressing SNU2535 cells with ALK + NSCLC. SNU2535 cells expressed extremely low levels of FGFR1 and FGF2 proteins (FGFR1^low^ and FGF2^low^) compared to NCI-H2228 cells (FGFR1^high^ and FGF2^high^) (Fig. 2d). Alectinib-induced inhibition of cell growth and AKT and ERK phosphorylation were restored in both FGFR1- and FGF2-overexpressing cells but not in control or FGF2-overexpressing cells (Fig. 2e, f).

Furthermore, to assess the generalizability of this study, we performed additional experiments with DTP cells using four ALK + NSCLC cells (three types of SNU-2535 cells transfected with control, FGF2, or FGFR1 plus FGF2 overexpressing lentiviral vector (lenti-control, FGF2^high^, FGFR1^high^+FGF2^high^ SNU-2535)), and NCI-H3122 cells). The alectinib-induced DTP cells from lenti-control and lenti-FGF2^high^ SNU-2535 cells showed approximately the same sensitivity to ALK-TKIs (alectinib and lorlatinib) as the parental cells, and their insensitivity to FGFR-TKIs (BGJ398 and AZD4547) was maintained (IC_20_ values > 1000 nM) (Supplementary Fig. 4E). On the other hand, lenti-FGFR1^high^+FGF2^high^ SNU-2535 DTP cells were insensitive to ALK-TKIs (IC_50_ values > 1000 nM) and sensitive to FGFR-TKIs (IC_20_ values of 206.6 nM with BGJ398 and 111.4 nM with AZD4547) compared to the parental cells (Supplementary Fig. 4E). Lenti-FGFR1^high^+FGF2^high^ SNU-2535 DTP cells markedly upregulated the levels of not only FGF2 and CD44 proteins but also ERK phosphorylation compared to the other two DTP cells (Supplementary Fig. 4F). Significant upregulation of FGFR1 phosphorylation was observed in lenti-FGFR1^high^+FGF2^high^ SNU-2535 DTP cells compared to that before treatment (Supplementary Fig. 4G). Furthermore, BGJ398 did not inhibit ERK phosphorylation and induce apoptosis as determined by the level of cleaved PARP protein in both the lenti-control and lenti-FGF2^high^ DTP cells, whereas BGJ398 completely inhibited ERK phosphorylation and markedly enhanced cleaved PARP protein level in lenti-FGFR1^high^+FGF2^high^ SNU-2535 DTP cells (Supplementary Fig. 4H). Lenti-FGFR1^high^+FGF2^high^ SNU-2535 regrown cells, generated from DTP cells by culturing in alectinib-free medium for 21 days, restored sensitivity to alectinib (IC_50_ value 149.3 nM) and insensitivity to BGJ398 (IC_20_ value > 1000 nM) compared with the DTP cells (Supplementary Fig. 4I). These susceptibilities of the regrown cells were similar to those of parental cells. These findings in lenti-FGFR1^high^+FGF2^high^ SNU-2535 DTP cells are consistent with the results obtained in FGFR1^high^+FGF2^high^ NCI-H2228 DTP cells, suggesting that lenti-FGFR1^high^+FGF2^high^ SNU-2535 cells also escape alectinib-induced cell death through activation of FGFR1 signaling via FGF2 upregulation in DTP cells.

Finally, we used NCI-H3122 cells, which exhibit lower FGFR1 and FGF2 protein expression compared to NCI-H2228 cells (Supplementary Fig. 4J). Although alectinib-induced NCI-H3122 DTP cells with CD133 upregulation showed insensitivity to ALK-TKIs (IC_50_ values > 1000 nM) compared to parental cells, they did not increase the sensitivity to FGFR-TKIs (IC_20_ values > 1000 nM) nor did they show elevated FGFR1 nor FGF2 protein levels (Supplementary Fig. 4K, L). BGJ398 did not inhibit ERK phosphorylation or induce apoptosis in DTP cells (Supplementary Fig. 4M). These findings are consistent with the results of FGFR1^low^+FGF2^low^ SNU-2535 cells, indicating that FGFR1^low^+FGF2^low^ NCI-H3122 DTP cells escape alectinib-induced cell death by activating signaling pathways other than those involving FGFR1 and FGF2 proteins. Therefore, in this analysis using the four additional ALK + NSCLC cells, we can confirm again our findings in this study showing that the dependency of alectinib-induced DTP cells on FGFR1/FGF2 signaling would increase in basal FGFR1^high^ and FGF2^high^ ALK + NSCLC cells. These findings suggest that cell survival against ALK-TKIs is activated by FGFR1 signaling involving AKT or ERK phosphorylation in FGFR^high^ and FGF2^high^ ALK + NSCLC cells, and the FGFR1 and FGF2 proteins promote escape from ALK-TKI-induced cell death.

### *FGFR1*- and *FGF2*-expressing patients with ALK + NSCLC show poor response to ALK-TKIs

**Fig. 3:**
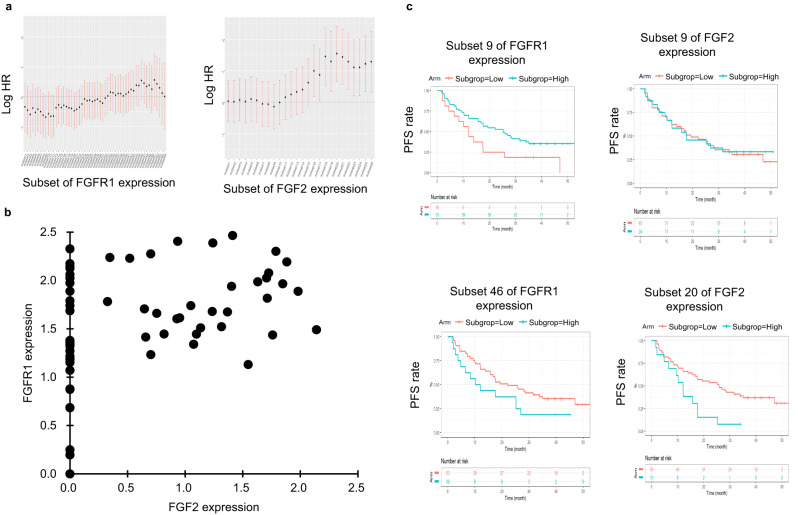
Impact of FGFR1 or FGF2 expression on response to ALK inhibitors in ALK + NSCLC patients. **a** Adjusted smooth log HR estimated with 95% confidence intervals for *FGFR1* or *FGF2* mRNA expression levels among patients in the J-ALEX study. **b** Scatter plot of *FGFR1* and *FGF2* mRNA expression level (Log_2_(TPM + 1)) for each patient. **c** Kaplan–Meir curves for PFS of patients with high or low-expression levels of *FGFR1* or *FGF2* mRNA in the subset of cases with the minimum or maximum log HR value from (**a**).

We retrospectively evaluated the association between the clinical efficacy of ALK-TKIs and *FGFR1* or *FGF2* expression levels using data from the J-ALEX phase III study of patients with ALK + NSCLC treated with alectinib or crizotinib^17^. Since the number of formalin-fixed, paraffin-embedded (FFPE) tumor samples collected before treatment with alectinib (*n* = 38) or crizotinib (*n* = 31) was limited, we assessed the relationship between both drugs and estimated hazard ratios (HR) using the various cut-off values for basal *FGFR1* or *FGF2* mRNA expression levels in tumors before starting the treatment determined by RNA sequencing, and estimated the HR of high to low-expression levels in each subset (Supplementary Fig. 5B). The number of patients and PFS events for each subset are shown in Supplementary Fig. 5C. We found that PFS against both *FGFR1* and *FGF2* tended to be short in an mRNA level-dependent manner (Fig. 3a). The group with the shortest PFS (log HR value over 1.0) had higher *FGF2* expression levels corresponding to subsets 18 to 26 (Fig. 3a). Although the results were unclear, patients with *FGF2*^high^ tended to express relatively high levels of *FGFR1* mRNA compared to patients with *FGF2*^low^ (Fig. 3b). When patients were categorized by the magnitude of *FGF2* mRNA expression in subset 20, which showed the maximum statistics for the multivariate Cox model, the PFS for patients with *FGF2*^high^ was shorter than that for *FGF2*^low^ patients (Fig. 3c). In contrast, when patients were categorized in subset 9, which showed the minimum statistics for the multivariate Cox model, PFS was similar between these patients. However, when we evaluated the relationship between *FGFR1* or *FGF2* expression and patient prognosis using the Gene Expression Profiling Interactive Analysis 2 database, high expression levels of *FGFR1* or *FGF2* were not significantly associated with poor prognosis in any cancer (Supplementary Fig. 5D). Collectively, the decreased clinical efficacy of ALK-TKIs for patients with *FGFR1*^high^ and *FGF2*^high^ was consistent with our nonclinical results in FGFR1^high^ and FGF2^high^ NCI-H2228 and FGFR1^low^ and FGF2^low^ SNU-2535 cells, suggesting that basal FGFR1^high^ and FGF2^high^ ALK + NSCLC tumors are associated with worse PFS in patients who received ALK-TKIs as a first-line treatment.

### Combined ALK- and FGFR-TKI suppresses the growth of FGFR1^high^ and FGF2^high^ ALK+cells

**Fig. 4:**
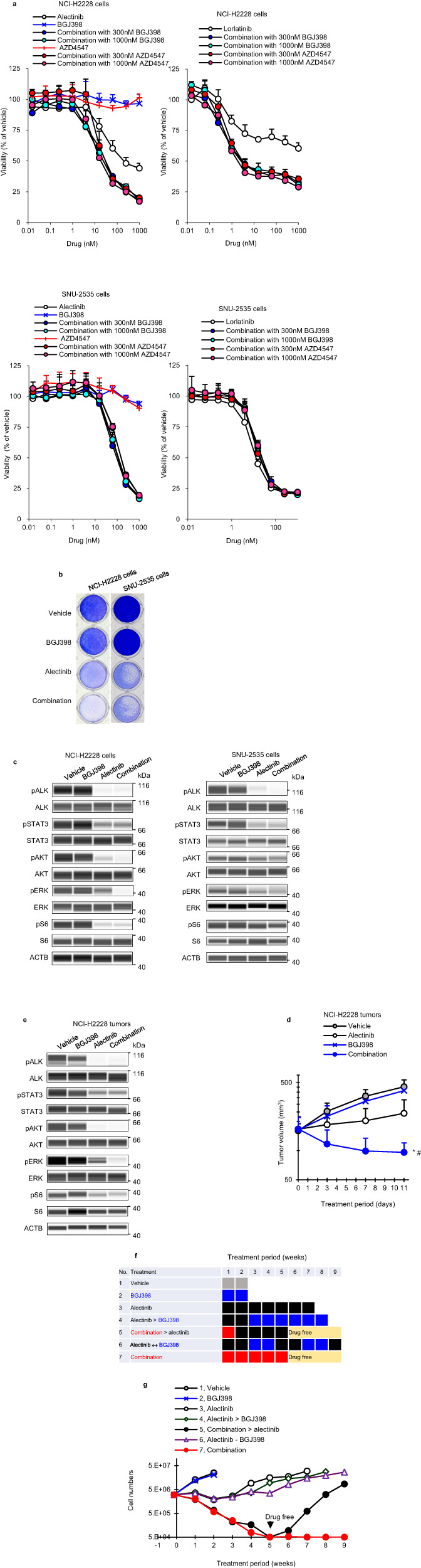
FGFR inhibition enhances effect of ALK inhibitor in FGFR1^high^ and FGF2^high^ cells. **a** Cells were cultured with alectinib, lorlatinib, BGJ398, or AZD4547, or alectinib, lorlatinib combined with BGJ398 or AZD4547 for 8 days. **b** Crystal violet growth assays following treatment with 1000 nM alectinib, 300 nM BGJ398, or a combination for 8 days. **c** Immunoblots of cell lysates treated with alectinib, 300 nM BGJ398, or a combination for 3 h. NCI-H2228 and SNU-2535 cells were treated with 1000 nM and 100 nM alectinib, respectively. **d** Mice with NCI-H2228 xenograft tumors were treated with vehicle, 2 mg/kg alectinib, 50 mg/kg BGJ398, or a combination for 11 days. Each point represents the mean + SD. *P < 0.05 versus alectinib, or ^#^*P* < 0.05 versus BGJ398; Wilcoxon rank sum test by the Holm–Bonferroni method. *n* = 6 mice per group. **e** Immunoblots of tumor lysates treated with vehicle, 2 mg/kg alectinib, 50 mg/kg BGJ398, or a combination for 6 h. **f** NCI-H2228 cells were treated with 1000 nM alectinib, 300 nM BGJ398, the combination or a sequence of both drugs, and washed after 5 weeks. **g** Cell numbers were measured by a cell counter. If the cell number was 5E4 or less, it was indicated as 5E4 due to the limitation of the cell counter.

To enhance the efficacy of ALK-TKIs in patients with FGFR1^high^ and FGF2^high^, we examined whether combined ALK- and FGFR-TKI suppresses proliferation of NCI-H2228 cells (Fig. 4a). Although NCI-H2228 cells were insensitive to FGFR-TKIs alone (BGJ398 and AZD4547) (IC_50_ values > 1000 nM), ALK-TKI (alectinib and lorlatinib)-induced cell growth inhibition and apoptosis were enhanced upon the combination with FGFR-TKIs (Fig. 4a, b, and Supplementary Fig. 6A). Co-treatment with alectinib and BGJ398 suppressed AKT and ERK phosphorylation compared to single agent treatment in NCI-H2228 cells (Fig. 4c). In contrast, there were no combinatorial effects on FGFR1^low^ and FGF2^low^ SNU-2535 cells. Therefore, the addition of FGFR-TKIs to ALK-TKIs can suppress the reactivation of cell survival signaling molecules through activation of FGFR1 kinase by binding FGFR1 and FGF2 proteins in FGFR1^high^ and FGF2^high^ ALK + NSCLC cells.

To evaluate the in vivo efficacy of these combinations, we treated mice xenografts of NCI-H2228 cells with BGJ398, alectinib, or a combination. NCI-H2228 tumors showed no response to BGJ398 alone, whereas the combination treatment resulted in significant tumor regression and a decrease in ERK phosphorylation compared with alectinib alone (Fig. 4d, e, Supplementary Fig. 6B, C). These in vivo combinatorial effects coincided with the in vitro effects on NCI-H2228 cells (Fig. 4a, b). Combination treatment was well tolerated, with no weight loss during treatment (Supplementary Fig. 6D).

We further evaluated the effects of sequential treatment of ALK- and FGFR-TKIs with the later withdrawal of FGFR-TKI on NCI-H2228 cells in vitro (Fig. 4f). Concurrent combination treatment (No. 6) markedly inhibited cell growth compared with sequential treatments (Nos. 4 and 7) after 5 weeks of treatment (Fig. 4g). The inhibitory effects of concurrent combination treatment (No. 7) and 1-week concurrent combination followed by 4 weeks of alectinib treatment (No. 5) were similar up to 5 weeks, whereas significant cell regrowth was observed in treatment No. 5, but not in treatment No. 7 (Fig. 4g). Therefore, it is important to continue concurrent combination therapies to suppress the growth of FGFR1^high^ and FGF2^high^ ALK + NSCLC cells.

### Combined inhibition of FGFR and EGFR, HER2, or BRAF enhances response in FGFR1^high^ and FGF2^high^ cells

**Fig. 5:**
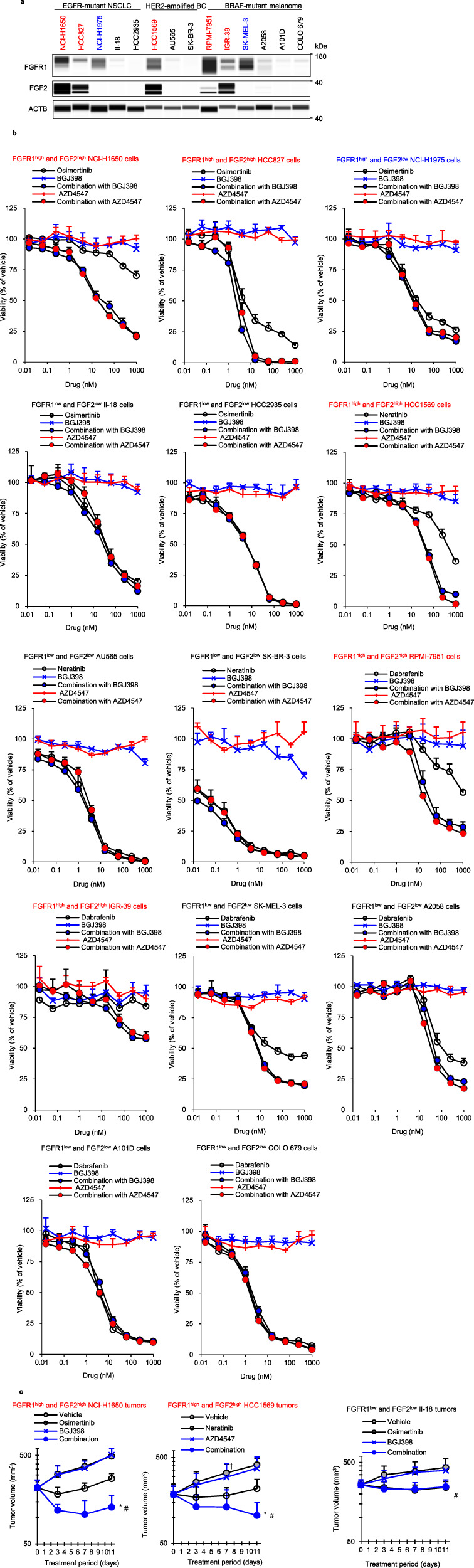
Effects of combination with FGFR inhibitors on EGFR-, HER2-, BRAF-mutant cells. **a** Immunoblots of cell lysates of NCI-H1650, HCC827, NCI-H1975, II-18, HCC2935, AU565, SK-BR-3, RPMI-7951, IGR-39, SK-MEL-3, A2058, A101D and COLO 679 cells. Cell lines highlighted in red express relatively high levels of both FGFR1 and FGF2 and those in blue color express relatively high levels of FGFR1, whereas cell lines in black express relatively low levels of FGFR1 and FGF2. **b** Cells were cultured with osimertinib, neratinib, dabrafenib, BGJ398, AZD4547, or osimertinib, neratinib, dabrafenib combined with 300 nM of BGJ398 or AZD4547 for 8 days. **c** Mice with xenograft tumors were treated with vehicle, 5 mg/kg osimertinib, 20 mg/kg neratinib, 50 mg/kg BGJ398, 10 mg/kg AZD4547 or the combination of both drugs for 11 days. *n* = 8, 6, and 6 mice per group in NCI-H1650, HCC1569, and II–18 tumors, respectively. Each point represents the mean + SD. *P < 0.05 versus osimertinib, neratinib, or ^#^*P* < 0.05 versus BGJ398, AZD4547; Wilcoxon rank sum test by the Holm–Bonferroni method. †: One mouse in the vehicle group was euthanized due to deterioration by fighting.

Next, we determined whether FGFR-TKIs combined with targeted agents other than ALK-TKIs enhanced responses. Among all cell lines tested in this study, FGFR1 was highly expressed in EGFR + NSCLC NCI-H1650, HCC827, NCI-H1975, and HER2 + BC HCC1569 cells and BRAF+melanoma RPMI-7951, IGR-39, and SK-MEL-3 cells, and FGF2 was highly expressed in NCI-H1650, HCC827, HCC1569, RPMI-7951, and IGR-39 cells (Fig. 5a). We treated each cell line with targeted agents against EGFR, HER2, BRAF, or MEK alone or combined with FGFR-TKIs. All cells were insensitive to FGFR-TKIs alone (IC_50_s > 1000 nM), but each combination strongly inhibited cell growth and induced apoptosis compared to targeted agents alone and enhanced suppression of ERK phosphorylation in all FGFR1^high^ and FGF2^high^ cells, but not in FGFR1^low^ and FGF2^low^ cells, regardless of the type of driver oncogenes (Fig. 5b, Supplementary Fig. 7A–D). Furthermore, there was no combinatorial effect of FGFR-TKIs with osimertinib in FGFR1^high^ and FGF2^low^ NCI-H1975 cells (Fig. 5b and Supplementary Fig. 7B).

In FGFR1^high^ and FGF2^high^ NCI-H1650 and HCC1569 xenograft tumors, co-treatment with FGFR-TKIs and EGFR-TKI osimertinib or HER2-TKI neratinib strongly inhibited tumor growth and ERK phosphorylation compared with osimertinib or neratinib alone, whereas FGFR1^low^ and FGF2^low^ II-18 xenograft tumors did not affect tumor growth (Fig. 5c and Supplementary Fig. 7E–G). Combination treatments were well tolerated, with no weight loss during treatment (Supplementary Fig. 7H).

To verify that both FGFR1 and FGF2 proteins are required for cell survival against these TKIs, we conducted knockdown of FGFR1 and FGF2 using siRNAs in FGFR1^high^ and FGF2^high^ NCI-H1650, HCC827, and RPMI-7951 cells. Each co-treatment with 1000 nM TKIs and siFGFR1 and or siFGF2 significantly inhibited the cell growth compared with siControl in each cell (Supplementary Fig. 7I). Therefore, FGF2-mediated FGFR1 activation significantly affects cell survival against each TKI.

These findings follow the results observed in ALK + NCI-H2228 cells and suggest that combination treatment with FGFR-TKIs and targeted agents is effective in FGFR1^high^ and FGF2^high^ cells having any driver oncogenes, indicating that FGFR1 protein expression alone is insufficient for the activation of FGFR1-induced survival against targeted agents, and coexistence with FGFR1 and FGF2 may be essential for FGFR1 activation to escape targeted agents-induced cell death.

### DTP cells escape EGFR- and HER2-TKI-induced cell death through activation of FGFR1 signaling

**Fig. 6:**
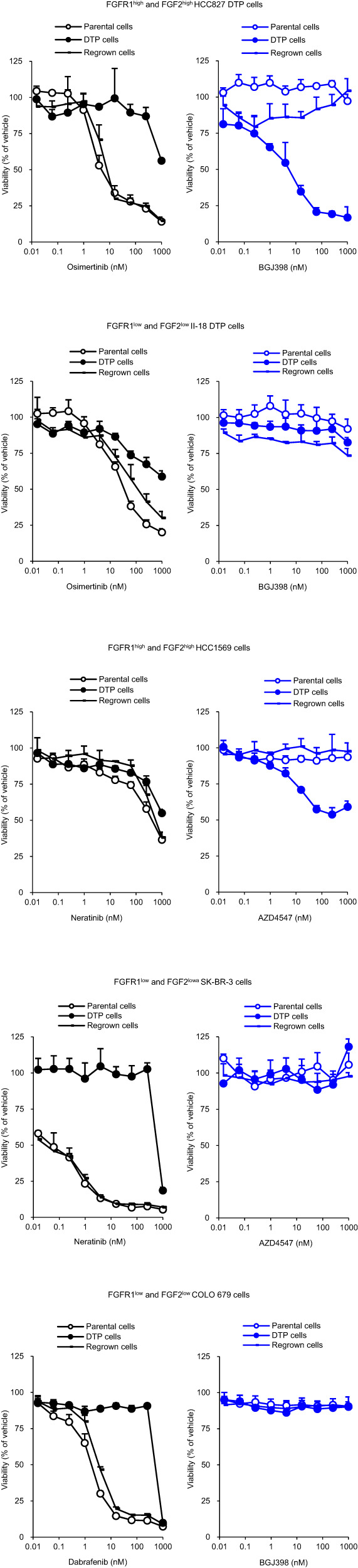
Effect of FGFR inhibitors on parental, DTP, and regrown cells with EGFR, HER2 or BRAF mutations. DTP cells were generated by treatment with 1000 nM osimertinib for 13 days (HCC827) or 36 days (II-18), 1000 nM neratinib (HCC1569) or 100 nM neratinib (SK-BR-3) for 13 days, or 100 nM dabrafenib for 13 days (COLO 679). Regrown cells were generated from DTP cells cultured in drug-free medium for 35 days (HCC827), 70 days (II-18), 30 days (HCC1569), 34 days (SK-BR-3), or 34 days (COLO 679). To perform cell proliferation assays, cells were cultured with osimertinib, neratinib, dabrafenib, or BGJ398 for 8 days.

We also assessed whether EGFR- or HER2-TKI induces a DTP state in FGFR1^high^ and FGF2^high^ cells by activation of FGFR1 survival signaling. Although these TKI-induced DTP cells showed the same features as H2228 DTP cells: upregulation of CD133 and a decrease in sensitivity to each targeted agent regardless of FGFR1 and FGF2 expression level, sensitivity to BGJ398 was increased in FGFR1^high^ and FGF2^high^ DTP cells, but not in FGFR1^low^ and FGF2^low^ DTP cells including BRAF + COLO 679 cells (Fig. 6 and Supplementary Fig. 8A). BGJ398 markedly inhibited AKT and ERK phosphorylation in FGFR1^high^ and FGF2^high^ DTP cells, but not in FGFR1^low^ and FGF2^low^ DTP cells (Supplementary Fig. 8B). The increased sensitivity to BGJ398 in FGFR1^high^ and FGF2^high^ DTP cells was lost in regrown cells. Therefore, the dependency of DTP cells on FGFR1 signaling may increase in FGFR1^high^ with FGF2^high^ cancer cells, regardless of tissue origin and driver oncogenes.

### FGFR1 signaling activation was maintained in resistance cells

**Fig. 7:**
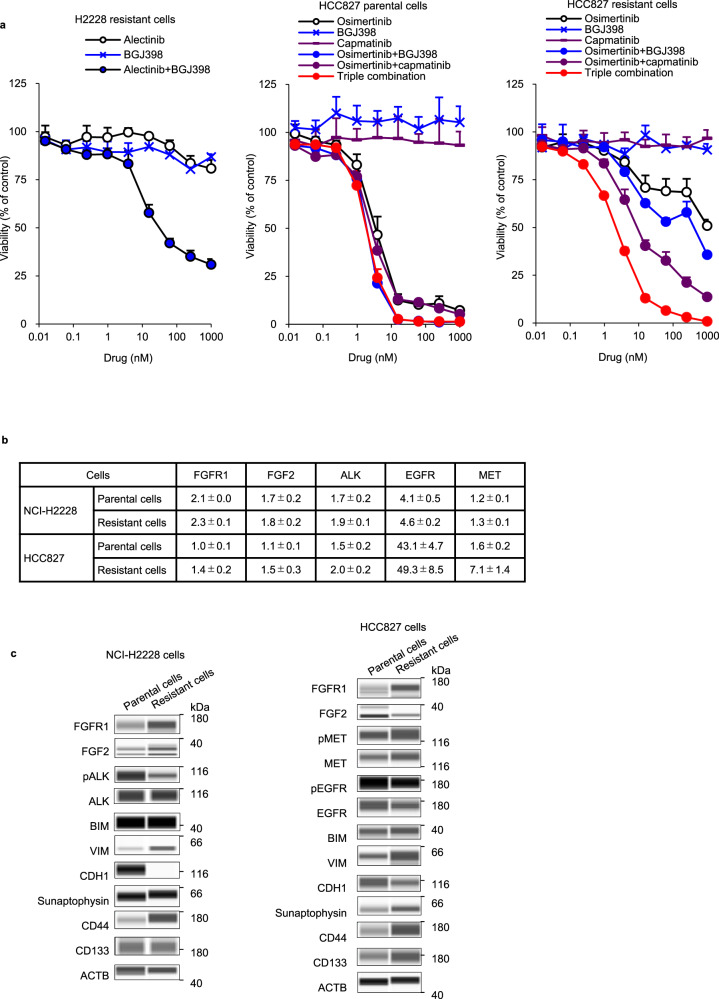
Combination with FGFR inhibitor is effective on alectinib- and osimertinib-resistant cells. **a** Cells were cultured with alectinib, osimertinib, capmatinib, BGJ398, or alectinib or osimertinib combined with 300 nM BGJ398 or 10 nM capmatinib and 300 nM BGJ398 for 8 days, respectively. **b** Gene copy numbers of parental and resistant cells. **c** Immunoblots of cell lysates of parental and resistant cells.

We investigated whether FGFR1 signaling is continuously activated after acquiring resistance to targeted treatment using alectinib-resistant NCI-H2228 and osimertinib-resistant HCC827 cells, established by exposing cells to 1000 nM of alectinib or osimertinib for 348 and 91 days, respectively. The IC_50_ values of resistant cells were >4.6- and 277.8 times higher than those of parental cells, respectively (Fig. 7a and Supplementary Fig. 9A).

In alectinib-resistant cells, no *ALK* mutation was detected, whereas upregulation of FGFR1 and FGF2 proteins was observed without an increase in *FGFR1* and *FGF2* copy number and induction of EMT and stemness characteristics (Fig. 7b, c). Unlike the alectinib-DTP cells, resistant cells were insensitive to BGJ398 alone (Supplementary Fig. 9A). However, combined BGJ398 and alectinib strongly inhibited cell growth and ERK phosphorylation compared to single agents in resistant cells (Fig. 7a and Supplementary Fig. 9A–C).

In osimertinib-resistant HCC827 cells, no *EGFR* resistance mutation was detected (data not shown), whereas upregulation of FGFR1 proteins without an increase in *FGFR1* copy number and the MET protein with an approximately 4.4-fold increase in *MET* copy number was observed (Fig. 7b). The characteristics of EMT and small cell lung cancer (SCLC) transformation were induced, indicated by an increase in synaptophysin protein (Fig. 7c). MET amplification has been reported in EGFR-TKI resistance cells^18^. Although a double combination of MET-TKI (capmatinib) plus osimertinib inhibited cell growth and inactivated ERK, a triple combination of capmatinib plus osimertinib plus BGJ398 was more effective in resistant cells (Supplementary Fig. 9A–C), indicating that FGFR1 survival signaling is partially retained when acquiring resistance to osimertinib, and MET-amplified resistant cells depend on EGFR, MET, and FGFR1 through *MET* amplification and upregulation of FGFR1 protein. Collectively, our findings suggest that activation of FGFR1 signaling is a mechanism underlying resistance to targeted therapy in FGFR1^high^ and FGF2^high^ cancer cells, and the addition of FGFR-TKIs to targeted therapy effectively suppresses the development of not only DTP cells but also resistance cells from treatment-naïve cancer in FGFR1^high^ and FGF2^high^ cancer, regardless of tissue origin or driver oncogenes.

## Discussion

Although genotype-directed target therapy is the standard of care and shows a dramatic response in many cancers, these therapies are rarely curative. In ALK+ and EGFR + NSCLC, a complete response is observed in < 5% of patients who receive ALK- and EGFR-TKIs^19,20^. Although small residual DTP cells can survive initial targeted TKI exposure, and develop drug resistance during treatment, predictive biomarkers underlying DTP mechanisms are unknown, despite target therapy-induced diverse DTP cells having been identified in vitro^6,21–26^. Therefore, it is essential to identify biomarkers before treatment to predict the weak response to targeted TKIs for establishing effective and less toxic DTP targeting therapies.

Here, we demonstrated that targeted TKIs for ALK, EGFR, or HER2-induced DTP cells generated from FGFR1^high^ and FGF2^high^ cells acquired a dependency on FGFR signaling by activation of downstream molecules, including AKT and ERK. Furthermore, knockdown of FGFR1, FGF2, or both increased targeted TKI-induced cell death, and co-treatment with FGFR-TKIs strongly enhanced the TKI-induced ERK inactivation, apoptosis, and cell death compared with single agents in FGFR1^high^ and FGF2^high^ cells. The FGFR and FGF ligand family comprises 4 and 23 members, and binding of FGFs to FGFRs activates FGFR kinase followed by downstream signaling components^12,27^.

To determine which of the FGFRs and FGFs, other than FGFR1 and FGF2, contribute to survival in cancer cells, we developed four FGFR-overexpressing cells using FGFR1^low^ and FGF2^low^ II-18 cells (Supplementary Fig. 10A) and treated them with osimertinib in the presence of seven exogenous FGF proteins including FGF1, FGF2, FGF7, FGF9, FGF11, FGF12, and FGF18 expressed in NSCLC tumors in the J-ALEX study (data not shown). The pairs of FGFR1 and FGF2 or FGF9; FGFR2 and FGF2, FGF7, or FGF9; FGFR3 and FGF2 recovered more strongly from osimertinib-induced cell death than other pairs (viability values > 2.0), and these effects were strongly abrogated following addition to BGJ398 (Supplementary Fig. 10B). Consistent with these results, the coexistence of FGFR1 or FGFR2 with FGF2 protein completely reactivated the osimertinib-induced decrease in phosphorylation of AKT, ERK, and S6, whereas the coexistence of FGFR3 or FGFR4 with FGF2 only partially reactivated the decrease in phosphorylation of ERK (Supplementary Fig. 10C). The addition of the FGF2 or FGF9 protein in FGFR1^high^ and FGF2^low^ NCI-H1975 cells strongly rescued osimertinib-induced cell death than the other five FGFs (Supplementary Fig. 10D). Collectively, these findings suggest that cancer cells and patients with basal high protein expression levels of FGFR1 with FGF2 or FGF9; FGFR2 with FGF2, FGF7, or FGF9; FGFR3 with FGF2 may show a weak response to targeted therapy due to the activation of survival signaling from FGFR kinase, and the addition of FGFR-TKIs could overcome cell survival against targeted therapy in such FGFR- and FGF-positive cases at baseline. In line with this hypothesis, EGFR + NSCLC cells that co-express FGFR1 or FGFR2 with FGF2 or FGF9 are insensitive to EGFR-TKIs^28^. Both FGFR1- and FGF2-expressing EGFR+cells showed an EMT phenotype and upregulate FGF9 in FGFR1-expressing ALK+cells exhibiting characteristics of SCLC, and such features underly the acquired DTP state along with resistance to targeted TKIs^6,29–32^. Collectively, cells expressing specific FGFRs and FGFs high at baseline may concurrently activate not only oncogenic signaling but also FGFR signaling by binding of steady-state FGF, and this autocrine activation of FGFR signaling with FGF may contribute to the evasion of initial targeted TKI-induced acute apoptosis as an alternative pathway for cell survival and generate FGFR-dependent DTP cells immediately after targeted TKI treatment, regardless of driver oncogenes.

In this study, all FGF2-expressing cells highly expressed FGFR1 regardless of oncogenic mutation, and most patients with FGF2^high^ ALK+ had relatively high FGFR1 expression. Consistent with these observations, among the FGFRs and FGFs, FGFR1 and FGFR2 with FGF2, and FGF9 were frequently co-expressed in 33 NSCLC cell lines^28^, and *FGFR1* mRNA expression levels were also significantly correlated with *FGF2* and *FGF9* mRNA levels in patients with SCLC^33^. Furthermore, when we screened 135 lung cancer and 1,182 other cancer cell lines, numerous cells that expressed high *FGF2* or *FGF9* showed relatively higher expression levels of *FGFR1*, *FGFR2*, or both (Supplementary Fig. 11A). However, there were some *FGF2*- or *FGF9*-high expressing cells with low expression of *FGFR1* or *FGFR2* (Supplementary Fig. 11A). This was also observed in FGF2- and FGF9-high expressing patients with bronchus and lung cancer (Supplementary Fig. 11B). Collectively, high expression of both these FGFRs and FGFs can be a biomarker for the selection of patients who show a low response to targeted TKIs but are expected to respond to combination therapy with FGFR-TKIs.

Although three FGFR-TKIs monotherapies have been approved by the FDA^34,35^, no combination therapy with FGFR and targeted TKIs is approved. To perform clinical studies with these combination treatments, multiple expression levels of FGFRs and FGFs and driver oncogenes must be detected. However, NGS-based tests to detect various oncogenic mutations, including FoundationOne, cannot measure mRNA levels and immunohistochemistry is also impractical due to limited samples and cost. RNAscope is a commercially available and automated multiplex assay that simultaneously detects up to 4 RNAs per slide, and is a highly sensitive and specific assay can detect one RNA in fixed frozen, and FFPE tissue samples^36^. In this study, FGFR1 and FGF2 RNA stained by RNAscope within an FFPE cell block of FGFR1^high^ and FGF2^high^ cells were higher than those of FGFR1^low^ and FGF2^low^ cells (Supplementary Fig. 12), suggesting that FGFR1 and FGF2 expression levels observed in RNAscope and western blotting were comparable and RNAscope could be used to detect specific FGFRs and FGFs expression levels in cells in future clinical studies combining FGFR and targeted TKIs.

Several preclinical studies have shown that FGFR signaling is upregulated by increased expression of several FGFRs and/or FGFs, including FGF2, FGF9, FGF13, FGFR1, FGFR2, and FGFR3, after acquired resistance to various targeted TKIs^37^. Upregulation of FGF9 protein and transdifferentiation to SCLC was reported in patients with EGFR + NSCLC after acquired resistance to EGFR-TKIs^31^. Here, we found a close relationship between DTP cells and resistant cells generated from FGFR1^high^ and FGF2^high^ cells: both can use alternative FGFR signaling for cell survival and growth, namely alectinib-DTP cells increase FGF2 expression, and alectinib-resistant cells increase both FGFR1 and FGF2 expression compared with NCI-H2228 parental cells. In contrast, the dependency on FGFR signaling is maintained in osimertinib-resistant MET-amplified cells by increasing FGFR1 expression compared with HCC827 parental cells, whereas no increase in FGFR1 and FGF2 expression was observed in osimertinib-DTP cells despite their dependence on FGFR signaling. Since the osimertinib-resistant cells increased the expression of an SCLC marker (synaptophysin), we speculate that HCC827 cells may use FGFR1 with FGF2 as well as FGF9 as alternative autocrine signals for cell survival in a DTP state, and resistant cells acquire resistance from the DTP state by MET amplification and maintain FGFR signal activation through FGFR1 upregulation. Further experiments are required to verify this hypothesis.

Consistent with our results, Jenks et al. reported that the combination with ALK-TKI and FGFR-TKI was effective compared with each agent alone in ALK-TKI resistance NCI-H2228 cells^38^. Terp et al. reported that EGFR-TKI resistance HCC827 cells also upregulated FGFR1 and MET proteins, and both combinations with EGFR-TKI plus FGFR-TKI and EGFR-TKI plus MET-TKI were effective^39^, indicating these observations are consistent with our findings of resistance cells from FGFR1^high^ and FGF2^high^ cells. Furthermore, the EGFR-TKI resistance cells from other FGFR1^high^ and FGF2^high^ NCI-H1650 or FGFR1^high^ and FGF2^low^ NCI-H1975 increased FGFR1 and FGF2 proteins or FGFR1 protein, respectively^40^. However, no EGFR-TKI resistant cells were established from FGFR1^low^ and FGF2^low^ HCC2935 cells^40^, whereas the resistance cells from FGFR1^low^ and FGF2^low^ II-18 cells increased FGFR1 protein level as well as the sensitivity to FGFR-TKI compared to parental cells^39^. Considering these observations and our findings, FGFR1 signal activation is maintained during the state from DTP to resistance in FGFR1^high^ and FGF2^high^ cells, and could also not occur in DTP but resistance state in FGFR1^high^ and FGF2^low^ as well as FGFR1^low^ and FGF2^low^ cells. However, the mechanism by which FGFR activation is maintained in resistant cells is unknown, and no amplification of these genes was detected as described in Fig. 7B. More studies are needed to analyze the mechanisms to increase the expression of FGFR1 or FGF2 protein in resistance cells, for selecting which cells activate FGFR1 signaling after acquired resistance in such FGFR1^high^ and FGF2^low^ or FGFR1^low^ FGF2^low^ cells.

Raoof et al. reported that EGFR-TKI-induced EGFR + NSCLC DTP cells increase expression of FGFR3 and multiple FGFs, including FGF2^29^. Furthermore, FGFR3 knockdown, but not FGFR1, prevents the cell growth of DTP cells, and cell proliferation was strongly inhibited by co-treatment with EGFR-TKI and FGFR-TKI compared with each single agent during 5 weeks treatment in all four EGFR + NSCLC cells tested including FGFR1^high^ and FGF2^low^ NCI-H1975. In our study, we reasoned that no combination effect was observed during the 8-day treatment in NCI-H1975 cells due to low expression of FGF2 unless FGFR1 showed high expression. However, it is conceivable that combination effect appears by longer-term exposure even in basal FGFR1^low^ or FGF2^low^ cells via increasing of FGFR3 and multiple FGFs expressions as described by Raoof et al. Therefore, further studies are needed whether the differences in basal FGFR1 or FGF2 expression levels affect the generation of DTP cells through FGFR3 as well as the potential for combination effects with FGFR-TKIs in long-term treatment.

However, activation of FGFR with FGF autocrine signaling in specific FGFR^high^ and FGF^high^ cells, including FGFR1 with FGF2 or FGF9; FGFR2 with FGF2, FGF7 or FGF9; FGFR3 with FGF2 may have a compensatory role in promoting the survival and growth of DTP and resistant cells during treatment with targeted TKIs alone, and combined FGFR and targeted TKIs may be a promising therapeutic strategy to prevent a targeted TKI-induced DTP state or drug resistance against these specific FGFR^high^ and FGF^high^ cancer at baseline with various driver oncogenes.

This study has limitations. First, the criteria for FGFR and FGF expression levels that may predict the efficacy of combined FGFR and targeted TKIs remain unclear. Second, the efficacy of this combination treatment for cells with each pair of FGFR1–4 and FGF1–23 other than those examined is unknown. Third, we demonstrated the efficacy of combination treatment with ALK-, EGFR-, HER2-, and BRAF-TKIs and FGFR-TKIs, but did not assess other targeted TKIs such as ROS1- and RET-TKIs, since no FGFR- and FGF-positive cell lines harboring such mutations were available to us. Fourth, following Rooney’s report that all FGFR-TKI sensitive cell lines had relatively high expression of FGFR1 protein using western blotting analysis^41^, we used western blotting analysis on FGF2 protein to FGFR1 with no cut-off/quantitative threshold, and assessed the relationship between the relative expression level of these proteins and the combined effect of FGFR-TKI with targeted TKIs. However, to suggest clinical application and analysis of FGFR1 and FGF2 protein expression in a huge number of cancer cells, we need further studies to precisely measure these protein levels in each tumor cells by a quantitative analysis using a standard curve such as ELISA, and set a specific cut-off/quantitative threshold to predict the presence or absence of combination effect with FGFR-TKIs. Fifth, we retrospectively performed an integrated analysis of crizotinib and alectinib in patients with ALK + NSCLC between high or low *FGFR1* and *FGF2* expression because of the small sample size. Since the efficacy of alectinib is much stronger than crizotinib^17^, we added the treatment regimen as a covariate in the multivariate analysis. However, other potential prognostic factors may have been unbalanced between the high- and low-expression groups in some subsets. Regarding the impact of the unmeasured confounder on the exploratory analysis for *FGF2*, the average HR in Subset 16–26, which showed similar strength of correlation between the expression level of *FGF2* and PFS, was 3.45. Given this HR, the E-value^42^ was estimated as 4.079 indicated that the observed average HR of 3.45 could be explained away by an unmeasured confounder that was associated with both TPM of *FGF2* and the PFS events by a risk ratio of 4.079-fold each, above and beyond the measured confounders, but weaker confounding could not do so. It should be noted that approximation used to convert the HR to RR for the derivation of the E-value may be biased, possibly around 1/1.45-fold to true RR^43^.In this regard, with the alternative average HR derived by multiplying the original average HR of 3.45 by 1/1.45, the “conservative” E-value was estimated to be 2.58. Given that our analysis was adjusted for well-known prognostic factors, these E-values implied that findings from our exploratory analysis would be moderately robust against unmeasured confounders. Sixth, due to the small sizes of the patient-derived FFPE specimens, we had to use the entire samples for RNA extraction to obtain enough RNA content for RNA sequencing. Thus, we were unable to assess the impact of stromal infiltrating cells on *FGFR1* or *FGF2* mRNA expression in this J-ALEX clinical study. A further prospective study is needed to analyze the influence of stromal cells to *FGFR1* or *FGF2* expressions using new specimens and the RNAscope system. Furthermore, we did not assess patients with cancer with other driver mutations, because such clinical samples were not available. Finally, treatment regimens using more than one anti-cancer drug often result in increased toxic side effects that can compromise patient safety and treatment efficacy. In this mouse model, combining FGFR and targeted TKIs did not show obvious toxicity affecting body weight, whereas combination treatment with erlotinib and the unselective FGFR-TKI (dovitinib) was terminated for patients with NSCLC due to adverse effects^44^. To overcome these limitations, more preclinical and prospective clinical studies are needed to assess the efficacy or tolerability of combination treatment with various targeted TKIs and highly selective FGFR inhibitors.

To the best of our knowledge, this study is the first to report that high expression levels of specific FGFRs and FGFs (FGFR1 with FGF2 or FGF9; FGFR2 with FGF2, FGF7, or FGF9; FGFR3 with FGF2) at baseline rapidly promotes tolerance and continuously maintains survival and growth of DTP and resistant cells during treatment with targeted TKIs in various driver oncogene-positive cancer cells, including those with ALK, EGFR, HER2, or BRAF mutations. Therefore, it is plausible that an initial dual blockade of FGFR and various driver oncogenes may be a potent treatment strategy for patients with basal high expression of such FGFRs and FGFs to prevent the development of intrinsic resistance to targeted TKIs, and lead to tumor eradication.

## Methods

### Cell lines

The supplier and culture medium of human cancer cell lines in this study are listed in Supplementary Table 1. The oncogenic driver mutations were referred from the COSMIC cell database (https://cancer.sanger.ac.uk/cosmic) and previous reports^45–48^. Cells were maintained at 37 °C under 5% CO_2_.

### Drugs and reagents

Alectinib was synthesized by Chugai Pharmaceutical Co., Ltd. (Tokyo, Japan). Erlotinib was provided by F. Hoffmann-La Roche Ltd. (Basel, Switzerland). Infrigatinib (BGJ398), osimertinib, neratinib, and dabrafenib were obtained from LC Laboratories (Woburn, MA, USA). AZD4547 and trametinib were obtained from ChemScene (Monmouth Junction, NJ, USA). Lorlatinib, capmatinib, and lapatinib were obtained from Selleck Chemicals (Houston, TX, USA). The anti-cancer compound library was obtained from TargetMol (Boston, MA, USA). All agents were dissolved in dimethyl sulfoxide (DMSO: Sigma-Aldrich, St. Louis, MO, USA) for in vitro assays and in a 6% (w/v) solution of Captisol (ChemScene) for in vivo assays. DMSO and Captisol were used as vehicle controls. Recombinant human FGF1, FGF2, FGF7, and FGF12 proteins were obtained from R&D Systems (Minneapolis, MN, USA) and dissolved in phosphate-buffered saline (PBS, Sigma-Aldrich). Recombinant human FGF9 and FGF18 proteins were obtained from Abcam (Cambridge, UK) and dissolved in distilled water. Recombinant human FGF11 protein was obtained from LifeSpan Biosciences (Seattle, WA, USA) and dissolved in Tris buffer (Horizon Discovery, Cambridge, UK).

### Generation of DTP cells

NCI-H2228 DTP cells were generated from parental cells by treatment with 1000 nM alectinib for 13 days. HCC827 DTP cells were generated by treatment with 1000 nM osimertinib for 13 days. II-18 DTP cells were generated by treatment with 1000 nM osimertinib for 36 days. HCC1569 and SK-BR-3 DTP cells were generated by treatment with 1000 nM and 100 nM neratinib for 13 days, respectively. COLO 679 DTP cells were generated by treatment with 100 nM dabrafenib for 13 days.

### Anti-cancer compound library screen of NCI-H2228 cells

NCI-H2228 parental and DTP cells were seeded in 384-well plates, and 3114 agents in the anti-cancer compound library were added at 100 nM on the following day. After 6 days, cell viability was determined using the CellTiter-Glo 3D Cell Viability Assay kit. The viability of the agent- versus vehicle treatment was measured, and the ratio of the antiproliferative effect of each agent on DTP cells was calculated as follows: viability of DTP cells/viability of parental cells. Agents with an antiproliferative effect less than 0.7 and with the viability of parental cells between 0.9 and 1.1 were selected as candidate compounds.

### Cell proliferation assay

Cells were seeded in 384-well plates and drugs were added at the indicated concentrations the next day. After 8 days, cell viability was determined by quantification of cellular ATP, which indicates metabolically active cells, using the CellTiter-Glo 3D Cell Viability Assay (Promega, Madison, WI, USA). The viability of the agents was measured relative to the viability of the vehicle. Each point represents the mean + standard deviation of the triplicate experiments. The IC_50_, IC_40_, and IC_30_ values were calculated as previously described^49^.

### Western blotting

Cells were seeded in 6-well plates and drugs were added at the indicated concentrations the next day and cultured for the indicated time. The same amount of protein lysate was loaded for each western blot assay using the Sally Sue or Jess capillary electrophoresis-based protein analysis system (ProteinSimple, Santa Clara, CA, USA) according to the manufacturer’s protocol. Antibodies against ALK, phospho-ALK, FGFR1, FGFR2, FGFR3, FGFR4, ERK, phospho-ERK, AKT, phospho-AKT, EGFR, phospho-EGFR, MET, phospho-MET, β-actin, HER2, CD44, CD133, BIM, VIM, CDH1, S6, phospho-S6, and STAT3, phospho-STAT3 (Cell Signaling Technology, Danvers, MA, USA), cleaved PARP, phospho-HER2, FGF2 (Abcam) were used. The antibodies used in this study are shown in Supplementary Table 2. Uncropped immunoblots blots of each Figure are included in Supplementary Fig. 13.

### ELISA assay

Cells were seeded in 6-well plates and the following day, drugs were added at the indicated concentrations and cultured for the indicated time. The same amount of protein lysate was loaded for each ELISA assay conducted using the PathScan phospho-FGFR1 Sandwich ELISA Kit (Cell Signaling Technology) according to the manufacturer’s protocol.

### Apoptosis assay

Cells were seeded in 384-well plates and the following day, drugs were added at the indicated concentrations and cultured for the indicated time. Activity caspases 3 and 7 were determined using the Caspase-Glo 3/7 Assay (Promega). The activity was corrected for cell viability determined as described above and relative activity with the agents against the vehicle was calculated. Each point represents the mean + standard deviation of the triplicate experiments.

### Knockdown of FGFR1 or FGF2 by siRNA

Cells were transfected with ON-TARGETplus predesigned siRNA targeting *FGFR1*, *FGF2*, or nontargeting control (Horizon Discovery) using NEPA21 electroporation (Nepa Gene, Chiba, Japan) according to the manufacturer’s protocol, and the next day, drugs were added at the indicated concentrations and cultured for 6 days. Knockdown of FGFR1 or FGF2 was confirmed by observing the loss of each protein by western blotting after two days of siRNA transfection. The　target sequence of siRNAs used in this study is shown in Supplementary Table 3.

### Establishment of FGFR1- and FGF2-knockout cells by CRISPR/Cas9

NCI-H2228 cells were transfected with Cas9 Nuclease protein NLS (Nippon Gene, Tokyo, Japan), Edit-R tracrRNA, and crRNA-targeting human *FGFR1* (5’-GCATGGTTGACCGTTCTGGA-3’) or *FGF2* (5’-ATGTGGCACTGAAACGAACT-3’) (Fasmac, Kanagawa, Japan) constructs by NEPA21 electroporation. Subsequently, each clone was isolated using a Smart Aliquotor (NT Science, Aichi, Japan), and the *FGFR1*- or *FGF2*-knockout cells were confirmed by western blotting.

### Establishment of FGFR1- and FGF2-overexpressing SNU-2535 cells

The human CDS for the *FGFR1*, *FGF2*, or nontargeting control was synthesized, cloned, and inserted downstream of the EF1A promoter in a lentiviral vector by VectorBuilder (Chicago, IL, USA). SNU-2535 cells were infected with lentiviral vectors with a multiplicity of infection of 3 (MOI = 3) overnight according to the manufacturer’s protocol. On the following day, the culture medium was replaced with a puromycin-containing medium for 4 days to select FGFR and FGF2-overexpressing cells, and each stable clone was isolated using the Smart Aliquotor. Cells were maintained in a normal culture medium without puromycin, and FGFR1 or FGF2 protein expression was confirmed using western blotting.

### Establishment of FGFR1, 2, 3, 4–overexpressing II-18 cells

The human CDS for the four *FGFRs* (*FGFR1*, *FGFR2, FGFR3*, and *FGFR4*) or nontargeting control was synthesized, cloned, and inserted downstream of the EF1A promoter in a lentiviral vector by VectorBuilder. II-18 cells were infected with lentiviral vectors at MOI = 10 overnight according to the manufacturer’s protocol. On the following day, the culture medium was replaced with puromycin- or G418-containing medium for 4 or 8 days, respectively, to select FGFR-overexpressing cells. Cells were maintained in a normal culture medium without antibiotics, and expression levels of four FGFRs were confirmed using western blotting.

### Establishment of alectinib- and osimertinib-resistant cells

NCI-H2228 and HCC827 cells were exposed to 1000 nM alectinib and osimertinib for 348 and 91 days, respectively. After washing, cells were cultured in a medium without drugs for over 60 days. Resistance to each drug was confirmed using a cell proliferation assay.

### Detection of the EGFR mutation

Genomic DNA was obtained from cells using a Maxwell 16 Tissue DNA Purification Kit (Promega). *EGFR* mutations were measured using the AmoyDx EGFR 29 Mutations Detection Kit and fluorescent PCR (Amoy Diagnostics, Xiamen, China, Catalog number AMO-8.01.20201X024E) with a LightCycler 480 System (Roche Diagnostics, Basel, Switzerland), and positive mutations were defined based on the Ct value according to the manufacturer’s instructions.

### Detection of the ALK mutation

Briefly, DNA samples were quantified using a NanoDrop One Spectrophotometer (Thermo Fisher Scientific), and the DNA integrity number was determined using a Genomic DNA ScreenTape (Agilent, Santa Clara, CA, USA) on an Agilent 2200 TapeStation. Next, DNA sequencing libraries were prepared using SureSelect XT Low Input (Agilent) and SureSelect Human All Exon V7 (Agilent), and sequenced paired-end 100 bp with a NovaSeq6000 (Illumina). Reads were mapped to the human reference genome build hg38 using Burrows-Wheeler Aligner ver.0.7.17, and somatic SNVs and small indels were identified in the genomic data using Strelka ver.2.9.10.

### Copy number analysis

Copy numbers of DNA were measured using the predesigned TaqMan copy number probe (Thermo Fisher Scientific) for human *FGFR1*, *FGF2*, *ALK*, *EGFR*, and *MET* with quantitative real-time PCR using a LightCycler 480 System. Copy numbers normalized to the reference control gene of human RNase P were analyzed using CopyCaller ver. 2.1 software (Thermo Fisher Scientific) with human genomic DNA (Promega) as diploid control DNA.

### Animals

All animal experiments were reviewed and approved by the Institutional Animal Care and Use Committee at Chugai Pharmaceutical Co., Ltd., which is an institute accredited by AAALAC International and conformed to the Guide for the Care and Use of Laboratory Animals published by the Institute for Laboratory Animal Research. Male 5-week-old SCID mice (C.B-17/Icr-scid/scidJcl) were purchased from CLEA Japan, Inc (Tokyo, Japan). Male 4–5-week-old BALB/c-nu/nu mice (CAnN Cg-Foxn1<nu > /CrlCrlj nu/nu) were purchased from Charles River Laboratories Japan, Inc (Yokohama, Japan). All animals were acclimatized for > 5 days prior to the study. Chlorinated water and irradiated food were provided ad libitum, and the animals were kept in a controlled 12/12 h light-dark cycle.

### Mouse xenograft models

Mice were inoculated subcutaneously with 5 × 10^6^ cells per mouse on the right flank. Tumor volume and body weight were measured twice a week, and tumor volume was estimated as follows: tumor volume = ab^2^/2, where a and b are tumor length and width, respectively. After tumor establishment, mice were randomly allocated to 11-day treatment with the vehicle or indicated drug or drug combination in which each compound was orally administered daily at the same dose and schedule as the single agent. The mice were euthanized by exsanguination by cutting the aorta under isoflurane anesthesia. The tumors were harvested, frozen in liquid nitrogen, and crushed using a Multi-beads Shocker (Yasui kikai, Osaka, Japan). After adding and mixing of tumor samples in lysis buffer (Cell signaling), the lysates were centrifuged and the supernatants were used for Western blotting as tumor lysate.

### Retrospective analysis of the J-ALEX study

Japanese patients who were ALK inhibitor-naïve and chemotherapy-naïve or those who had received one prior chemotherapy regimen were enrolled and randomized to receive alectinib (*n* = 103) or crizotinib (*n* = 104) until progressive disease, unacceptable toxicity, death, or withdrawal in the phase III J-ALEX study (JapicCTI-132316; JO28928)^17^. In this study, we obtained 80 FFPE tumor specimens before the start of treatment with alectinib or crizotinib and stored them at 4 °C until use.

This study was conducted in accordance with the Declaration of Helsinki and Good Clinical Practice in Japan. The study protocol of JO28928 was reviewed and approved by the institutional review board from the perspective of ethical, scientific, and medical validity in Chugai Pharmaceutical Co. Ltd. All patients provided written informed consent prior to any study-related procedures.

### RNA sequence

Briefly, RNA samples were quantified using a NanoDrop One Spectrophotometer, and the RNA integrity number was determined using an RNA 6000 Nano Kit (Agilent) on an Agilent 2100 Bioanalyzer. Next, RNA sequencing libraries were prepared using the SMART-Seq Stranded Kit (Takara, Shiga, Japan). Since the library was less than 2 nM, we excluded six samples. The libraries were then pooled and sequenced, paired-end 150 bp with NovaSeq6000. Fastq sequence files were aligned with the human reference genome (hg38) using STAR 2.7.8a, and the reads were processed using StrandNGS 4.0 (Agilent). Since the mapping rate was less than 40%, we excluded five samples and analyzed the remaining 69 samples. The read counts for each gene were quantified as TPM.

### Statistical analysis

A multivariate Cox proportional hazard model was used to estimate the adjusted hazard ratio (HR) and the 95% confidence interval of PFS against ALK-TKIs associated with *FGFR1* or *FGF2* mRNA expression levels in each subset. Subsets were derived by changing category definitions in accordance with the observed TPM value of *FGFR1* or *FGF2* mRNA as cut-off values ranging from 0 to the 90^th^ percentile of the log_2_(TPM + 1) scale. In each subset, the analysis involved adjustment for covariates known to be associated with PD or death, including treatment drugs, ECOG performance status (PS) at baseline, treatment line, disease status, and brain metastases at baseline by IRF, which were used for the estimation (Supplementary Fig. 5A). Kaplan–Meier methodology was used to estimate the distribution of PFS for specific cut-off values. These clinical analyses were conducted with R Studio version 1.0153 and R version 3.6.0. In vitro, the experimental data were analyzed by Student’s *t* test or Dunnett’s test. In vivo, experimental data were analyzed using the Wilcoxon rank sum test, followed by the Holm–Bonferroni method. All statistical analyses were performed with JMP Ver. 15.0.0 (SAS Institute, Cary, NC, USA). The significance was established at a two-tailed *P* < 0.05.

### Reporting summary

Further information on research design is available in the Nature Research Reporting Summary linked to this article.

### Supplementary information

Supplementary Information

Supplementary Data

Reporting Summary

## Data Availability

Qualified researchers may request access to individual patient-level data of J-ALEX study through the clinical study data request platform (https://www.clinicalstudydatarequest.com/Default.aspx). For further details on Chugai’s Data Sharing Policy and how to request access to related clinical study documents, see here (www.chugai-pharm.co.jp/english/profile/rd/ctds_request.html). RNA sequencing data of the J-ALEX study cannot be deposited with a third party involving any public database, because we did not obtain the patient’s informed consent for depositing their RNA data into a third-party repository. Therefore, we comply with the Act on the Protection of Personal Information in Japan and we will not disclose individual RNA sequencing data. Tumor specimens in J-ALEX study cannot be used by any other research institution because we did not obtain the patient’s informed consent. Other experimental data supporting the findings of this study are available within the article and its supplementary information files.

## References

[1] Weinstein IB, Joe A (2008). Oncogene addiction. Cancer Res..

[2] Pagliarini R, Shao W, Sellers WR (2015). Oncogene addiction: pathways of therapeutic response, resistance, and road maps toward a cure. EMBO Rep.

[3] Francies HE, McDermott U, Garnett MJ (2020). Genomics-guided pre-clinical development of cancer therapies. Nat. Cancer.

[4] Aldea M (2021). Overcoming resistance to tumor-targeted and immune-targeted therapies. Cancer Discov..

[5] Sharma SV (2010). A chromatin-mediated reversible drug-tolerant state in cancer cell subpopulations. Cell.

[6] Mikubo M, Inoue Y, Liu G, Tsao MS (2021). Mechanism of drug tolerant persister cancer cells: the landscape and clinical implication for therapy. J Thorac Oncol.

[7] Dagogo-Jack I (2019). Treatment with next-generation ALK inhibitors fuels plasma <em>ALK</em> mutation diversity. Clin. Cancer Res..

[8] Yoda S (2018). Sequential ALK inhibitors can select for lorlatinib-resistant compound mutations in ALK-positive lung cancer. Cancer Discov..

[9] Recondo G (2020). Molecular mechanisms of acquired resistance to MET tyrosine kinase inhibitors in patients with MET exon 14-mutant NSCLC. Clin Cancer Res.

[10] Cohen P, Cross D, Jänne PA (2021). Kinase drug discovery 20 years after imatinib: progress and future directions. Nat. Rev. Drug Discov..

[11] Tanimura K (2021). Inhibition of c-Jun N-terminal kinase signaling increased apoptosis and prevented the emergence of ALK-TKI-tolerant cells in ALK-rearranged non-small cell lung cancer. Cancer Lett..

[12] Ornitz DM, Itoh N (2015). The fibroblast growth factor signaling pathway. Wiley Interdiscip Rev. Dev. Biol..

[13] Perera TPS (2017). Discovery and pharmacological characterization of JNJ-42756493 (Erdafitinib), a functionally selective small-molecule FGFR family inhibitor. Mol. Cancer Ther..

[14] Tiong KH, Mah LY, Leong C-O (2013). Functional roles of fibroblast growth factor receptors (FGFRs) signaling in human cancers. Apoptosis.

[15] Hangauer MJ (2017). Drug-tolerant persister cancer cells are vulnerable to GPX4 inhibition. Nature.

[16] Takezawa K, Okamoto I, Nishio K, Jänne PA, Nakagawa K (2011). Role of ERK-BIM and STAT3-survivin signaling pathways in ALK inhibitor–induced apoptosis in EML4-ALK–positive lung cancer. Clin. Cancer Res..

[17] Nakagawa K (2020). Final progression-free survival results from the J-ALEX study of alectinib versus crizotinib in ALK-positive non-small-cell lung cancer. Lung Cancer.

[18] Jakobsen KR (2017). MET amplification and epithelial-to-mesenchymal transition exist as parallel resistance mechanisms in erlotinib-resistant, EGFR-mutated, NSCLC HCC827 cells. Oncogenesis.

[19] Camidge DR (2019). Updated efficacy and safety data and impact of the EML4-ALK fusion variant on the efficacy of alectinib in untreated ALK-positive advanced non-small cell lung cancer in the global phase III ALEX study. J. Thoracic Oncol..

[20] Soria J-C (2017). Osimertinib in untreated EGFR-mutated advanced non-small-cell lung cancer. N. Engl. J. Med..

[21] Tsuji T (2020). YAP1 mediates survival of ALK-rearranged lung cancer cells treated with alectinib via pro-apoptotic protein regulation. Nat. Commun..

[22] Kurppa KJ (2020). Treatment-induced tumor dormancy through YAP-mediated transcriptional reprogramming of the apoptotic pathway. Cancer Cell.

[23] Tanimura K (2022). HER3 activation contributes toward the emergence of ALK inhibitor-tolerant cells in ALK-rearranged lung cancer with mesenchymal features. npj Precision Oncol..

[24] Taniguchi, H. et al. AXL confers intrinsic resistance to osimertinib and advances the emergence of tolerant cells. *Nat. Commun*. **10**, 10.1038/s41467-018-08074-0 (2019). 10.1038/s41467-018-08074-0 PMC6335418 30651547

[25] Yi Y (2018). Cancer-associated fibroblasts promote epithelial-mesenchymal transition and EGFR-TKI resistance of non-small cell lung cancers via HGF/IGF-1/ANXA2 signaling. Biochim. Biophys. Acta (BBA)—Mol. Basis. Dis..

[26] Arasada, R. R. et al. Notch3-dependent β-catenin signaling mediates EGFR TKI drug persistence in EGFR mutant NSCLC. *Nat. Commun*. **9**, 10.1038/s41467-018-05626-2 (2018). 10.1038/s41467-018-05626-2 PMC6090531 30097569

[27] Johnson DE, Williams LT (1993). Structural and functional diversity in the FGF receptor multigene family. Adv. Cancer Res..

[28] Marek L (2009). Fibroblast growth factor (FGF) and FGF receptor-mediated autocrine signaling in non-small-cell lung cancer cells. Molecular Pharmacology.

[29] Raoof S (2019). Targeting FGFR overcomes EMT-mediated resistance in EGFR mutant non-small cell lung cancer. Oncogene.

[30] Barretina J (2012). The Cancer Cell Line Encyclopedia enables predictive modelling of anticancer drug sensitivity. Nature.

[31] Ishioka K (2021). Upregulation of FGF9 in lung adenocarcinoma transdifferentiation to small cell lung cancer. Cancer Res..

[32] Leonetti A (2019). Resistance mechanisms to osimertinib in EGFR-mutated non-small cell lung cancer. Br. J. Cancer.

[33] Zhang L (2015). Fibroblast growth factor receptor 1 and related ligands in small-cell lung cancer. J. Thoracic Oncol..

[34] Jørgensen JT (2021). Oncology drug-companion diagnostic combinations. Cancer Treat. Res. Commun..

[35] Lee PC (2021). Targeting the Fibroblast Growth Factor Receptor (FGFR) in advanced cholangiocarcinoma: clinical trial progress and future considerations. Cancers (Basel).

[36] McGinnis LM, Ibarra-Lopez V, Rost S, Ziai J (2021). Clinical and research applications of multiplexed immunohistochemistry and in situ hybridization. J. Pathol..

[37] Zhou Y (2020). FGF/FGFR signaling pathway involved resistance in various cancer types. J. Cancer.

[38] Jenks AD (2018). Primary cilia mediate diverse kinase inhibitor resistance mechanisms in cancer. Cell Rep..

[39] Terp MG (2021). Combined FGFR and Akt pathway inhibition abrogates growth of FGFR1 overexpressing EGFR-TKI-resistant NSCLC cells. npj Precision Oncol..

[40] Ware KE (2013). A mechanism of resistance to gefitinib mediated by cellular reprogramming and the acquisition of an FGF2-FGFR1 autocrine growth loop. Oncogenesis.

[41] Rooney C (2016). Characterization of FGFR1 locus in sqNSCLC reveals a broad and heterogeneous amplicon. PLoS ONE.

[42] VanderWeele TJ, Ding P (2017). Sensitivity analysis in observational research: introducing the E-value. Ann. Intern. Med..

[43] VanderWeele TJ (2017). On a square-root transformation of the odds ratio for a common outcome. Epidemiology.

[44] Das M (2015). Dovitinib and erlotinib in patients with metastatic non-small cell lung cancer: a drug–drug interaction. Lung Cancer.

[45] Yoshimura Y (2016). Antitumor activity of alectinib, a selective ALK inhibitor, in an ALK-positive NSCLC cell line harboring G1269A mutation: Efficacy of alectinib against ALK G1269A mutated cells. Cancer Chemother. Pharmacol..

[46] Liu Q (2018). A novel HER2 gene body enhancer contributes to HER2 expression. Oncogene.

[47] Xiao J, Egger ME, McMasters KM, Hao H (2018). Differential expression of ABCB5 in BRAF inhibitor-resistant melanoma cell lines. BMC Cancer.

[48] Yuan J (2018). The dimer-dependent catalytic activity of RAF family kinases is revealed through characterizing their oncogenic mutants. Oncogene.

[49] Furugaki K (2016). Impact of bevacizumab in combination with erlotinib on EGFR-mutated non-small cell lung cancer xenograft models with T790M mutation or MET amplification. Int. J. Cancer.

