# Supplementary material for: FGFR blockade inhibits targeted therapy-tolerant persister in basal FGFR1- and FGF2-high cancers with driver oncogenes
Source: NPJ Precis Oncol. 2023 Oct 25;7:107. doi: 10.1038/s41698-023-00462-0 (PMC10600219; doi:10.1038/s41698-023-00462-0)
Supplement: Supplementary file 1 — Supplementary Information [file 41698_2023_462_MOESM1_ESM.pdf]

Index	Compound	Anti-proliferative effect on DTP cells	Targets
2788	THZ531	0.47	CDK12,CDK13
956	Hydrocortisone	0.62	Glucocorticoid Receptor
1415	Afatinib	0.65	EGFR (L858R); EGFR (L858R/T790M); EGFR (wt); HER2
1700	Poziotinib	0.67	HER1; HER2; HER4
2060	APY29	0.68	IRE1α
774	Dexamethasone	0.68	Annexin A1; Glucocorticoid Receptor; IL receptor; iNOS
737	Triamcinolone	0.69	Glucocorticoid Receptor
2301	AZD6482	0.69	DNA-PK; PI3Kα; PI3Kβ; PI3Kγ; PI3Kδ
2237	Erdafitinib	0.69	FGFR1; FGFR2; FGFR3; FGFR4

Supplementary Figure 1. Anti-cancer compound library screening in NCI-H2228 parental and DTP cells.

Nine compounds with strong anti-proliferative effects on DTP cells relative to parental cells are shown.

a

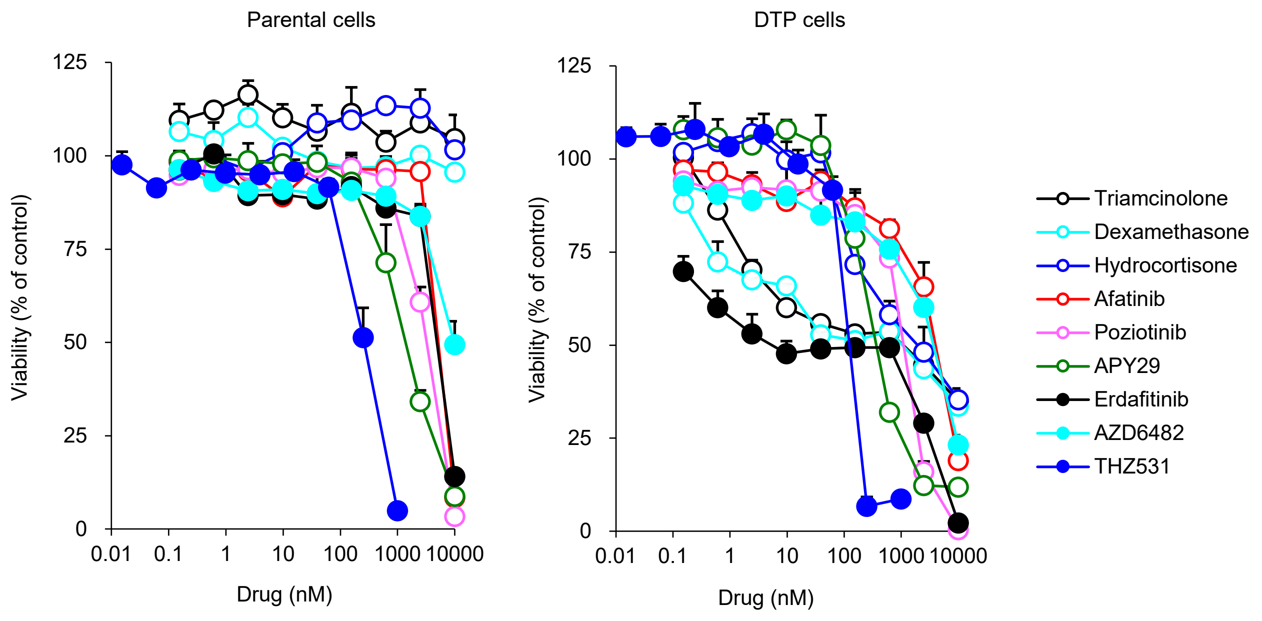

b

Compounds	IC_{50}		Ratio of parental to DTP cells
	DTP cells	Parental cells	
Erdafitinib	6.7	6136.4	920.8
APY29	444.8	1700.2	3.8
Poziotinib	1388.7	3915.8	2.8
AZD6482	4571.3	9873.5	2.2
THZ531	154.4	270.5	1.8
Afatinib	5022.5	6433.5	1.3
Dexamethasone	1322.1	>10000	ND
Triamcinolone	1364.3	>10000	ND
Hydrocortisone	2156.2	>10000	ND

c

FGFRs	mRNA expression (log_2(TPM+1))
FGFR1	5.85
FGFR2	0.07
FGFR3	0.62
FGFR4	0.80

Supplementary Figure 2. Comparison of inhibitory effects of the nine identified compounds on the growth of DTP cells as compared to the parental cells.

(**a**) Cells were cultured with nine compounds for 8 days. (**b**) IC_{50} values of the compounds, and the relative inhibitory effects on DTP cells relative to parental cells are shown. ND refers to cases where the relative inhibitory effect on DTP cells could not be calculated since the IC_{50} value in the parental cells was undetermined. (**c**) The mRNA levels of four FGFR members in NCI-H2228 cells were obtained from "Expression 21Q4 Public" of RNAseq TPM gene expression data in DepMap database (https://depmap.org/portal/download/).

a

Cells	Alectinib (nM)	Lorlatinib (nM)	BGJ398 (nM)
Parental cells	219.6	3.8	>1000
DTP cells	>1000	>1000	57.8
Regrown cells	480.0	ND	>1000

b

FGFs	mRNA expression (log_2(TPM+1))
FGF1	0.19
FGF2	4.90
FGF3	0.00
FGF4	0.00
FGF5	0.03
FGF6	0.00
FGF10	0.06
FGF19	1.00
FGF20	0.00
FGF21	0.34
FGF22	0.10

c

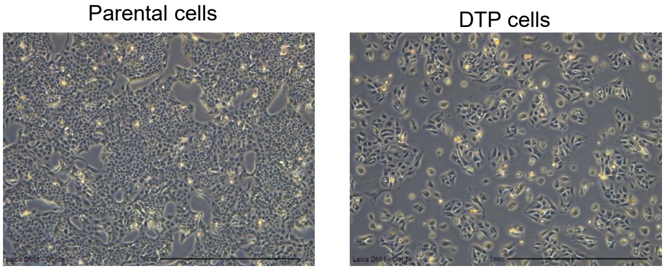

Supplementary Figure 3. Effect of ALK, or FGFR inhibitors on NCI-H2228 parental, DTP, and regrown cells.

(**a**) IC_{50} values to alectinib, and BGJ398, and IC_{30} values to lorlatinib of NCI-H2228 parental, alectinib-DTP, and alectinib-regrown cells. ND; Not determined. (**b**) The mRNA levels of 11 FGF family members of NCI-H2228 cells were obtained from "Expression 21Q4 Public" of RNAseq TPM gene expression data in DepMap database (https://depmap.org/portal/download/). (**c**) Microscopic examination of parental and alectinib-DTP cells. Scale bars indicate 1 mm.

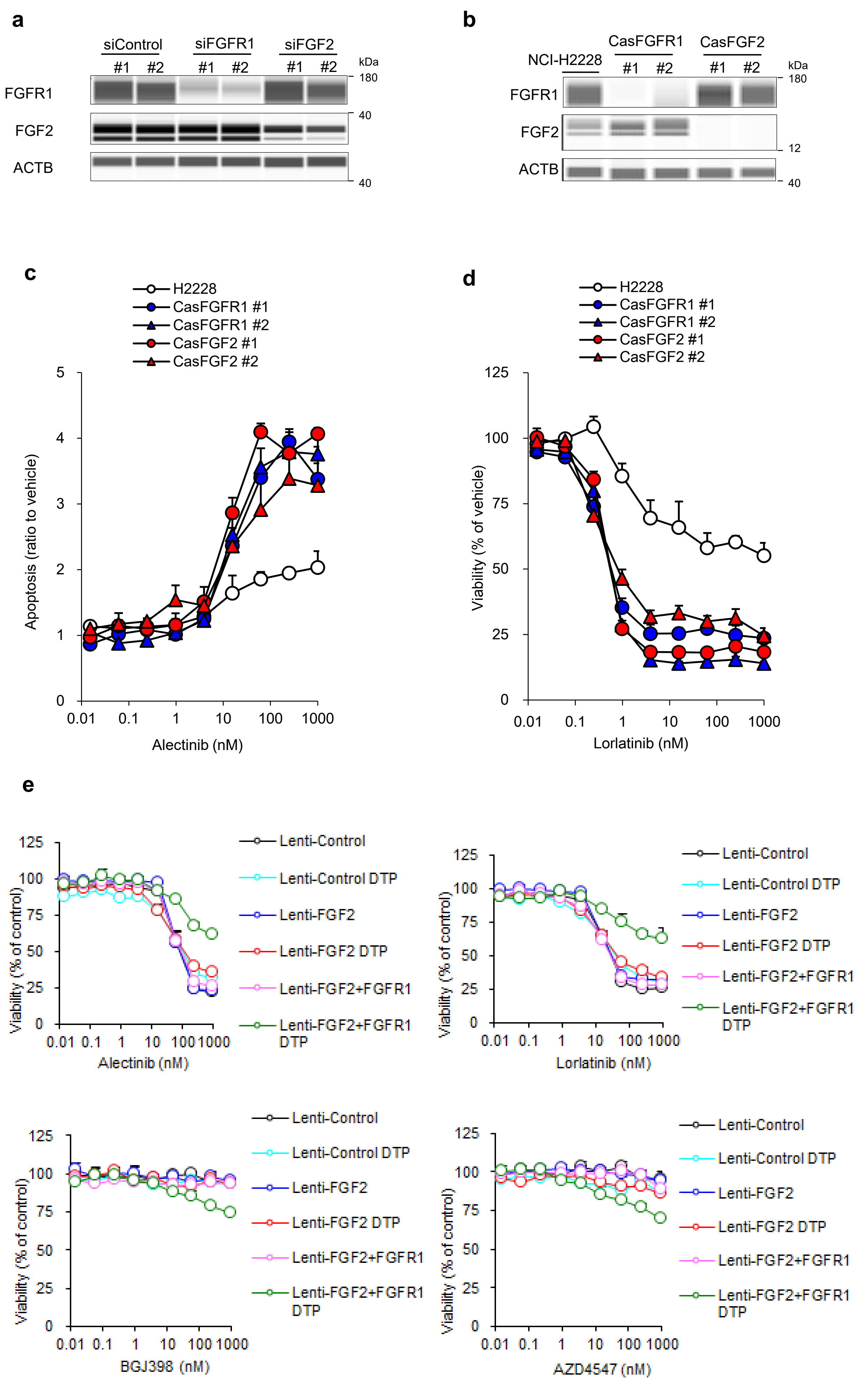

Cells	Alectinib (nM)	Lorlatinib (nM)	BGJ398 (nM)	AZD4547 (nM)
Lenti-control cells	98.5	35.8	>1000	>1000
Lenti-control DTP cells	124.7	48.0	>1000	>1000
Lenti-FGF2 cells	97.0	37.9	>1000	>1000
Lenti-FGF2 DTP cells	145.1	51.0	>1000	>1000
Lenti-FGFR1+FGF2 cells	107.6	35.2	>1000	>1000
Lenti-FGFR1+FGF2 DTP cells	>1000	>1000	206.6	111.4

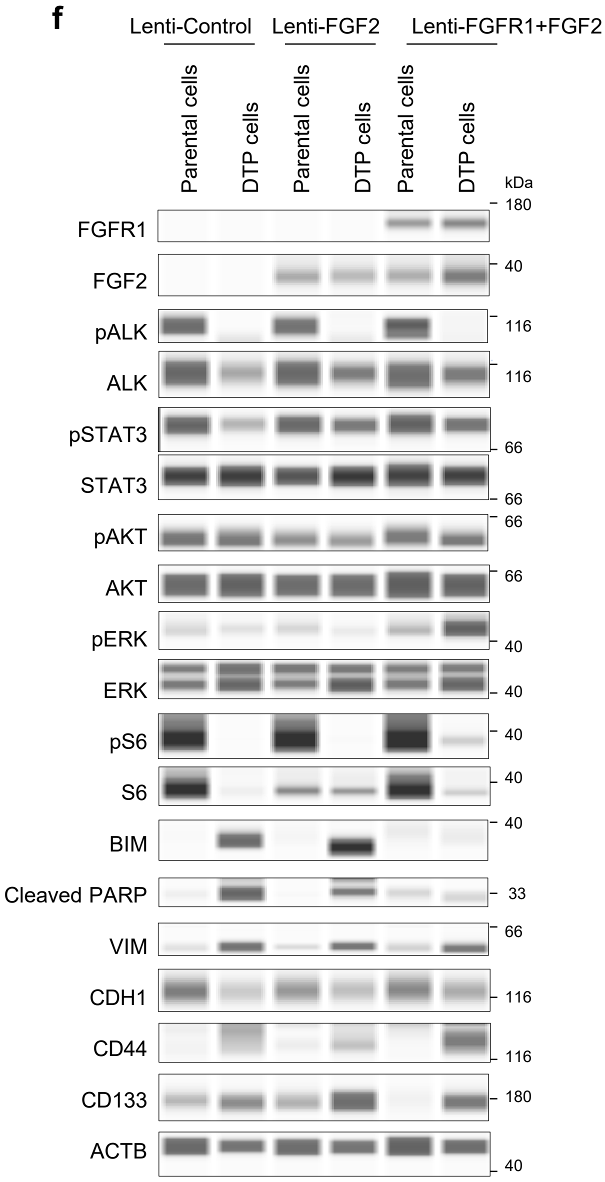
f
Lenti-Control
Lenti-FGF2
Lenti-FGFR1+FGF2
Parental cells
DTP cells
Parental cells
DTP cells
Parental cells
DTP cells
kDa
FGFR1
FGF2
pALK
ALK
pSTAT3
STAT3
pAKT
AKT
pERK
ERK
pS6
S6
BIM
Cleaved PARP
VIM
CDH1
CD44
CD133
ACTB
180
40
116
116
66
66
66
66
40
40
40
40
40
33
66
116
116
180
40

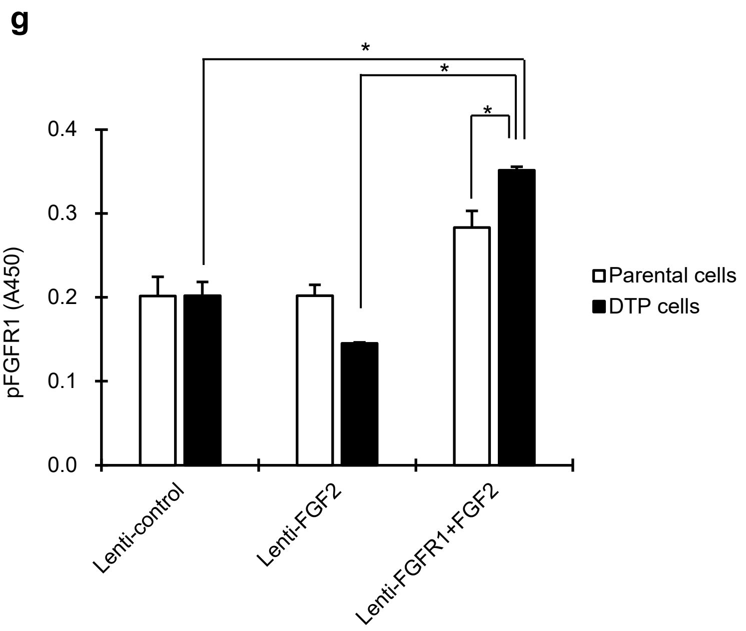
g
*
*
*
pFGFR1 (A450)
0.4
0.3
0.2
0.1
0.0
Lenti-control
Lenti-FGF2
Lenti-FGFR1+FGF2
Parental cells
DTP cells

h

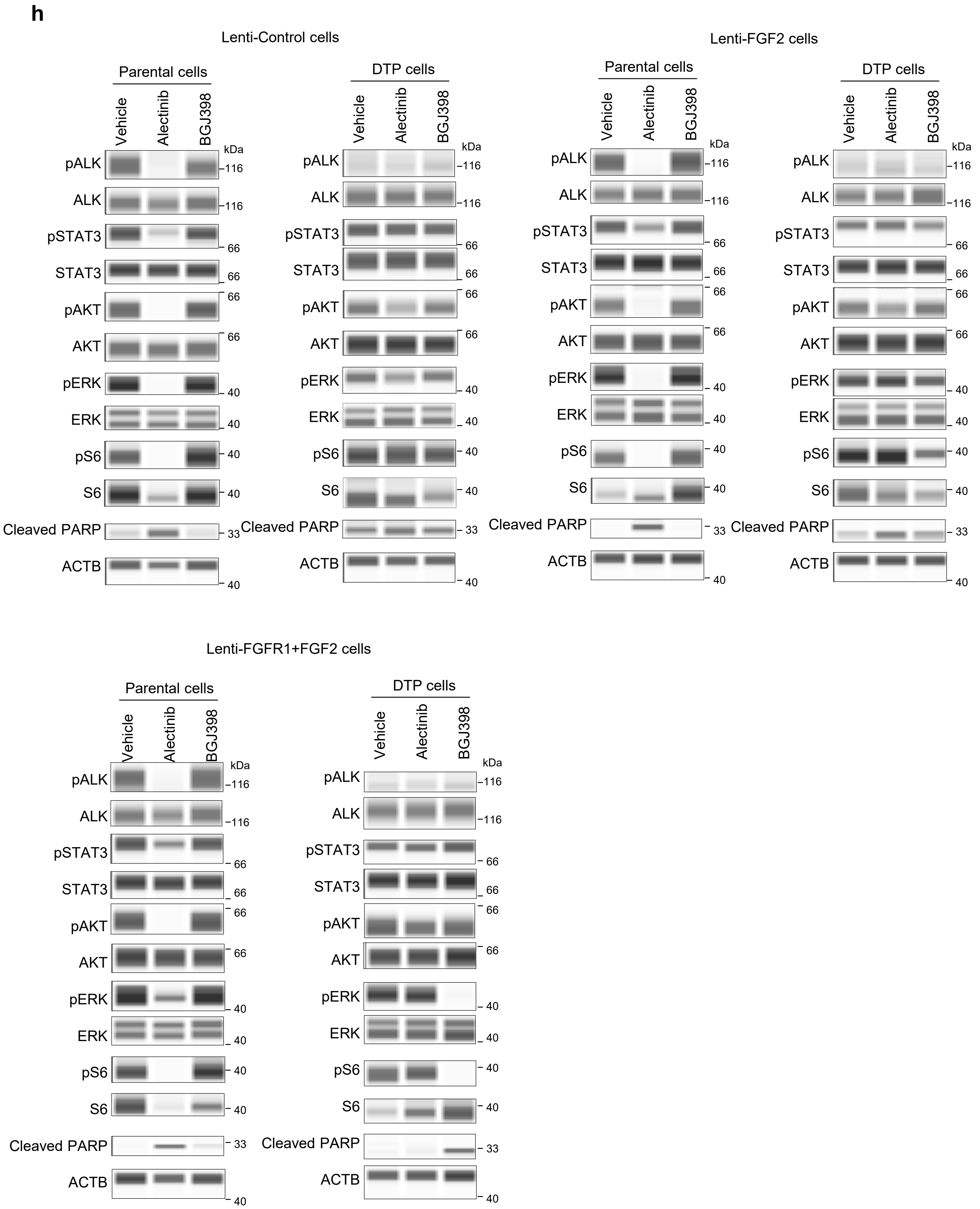

i

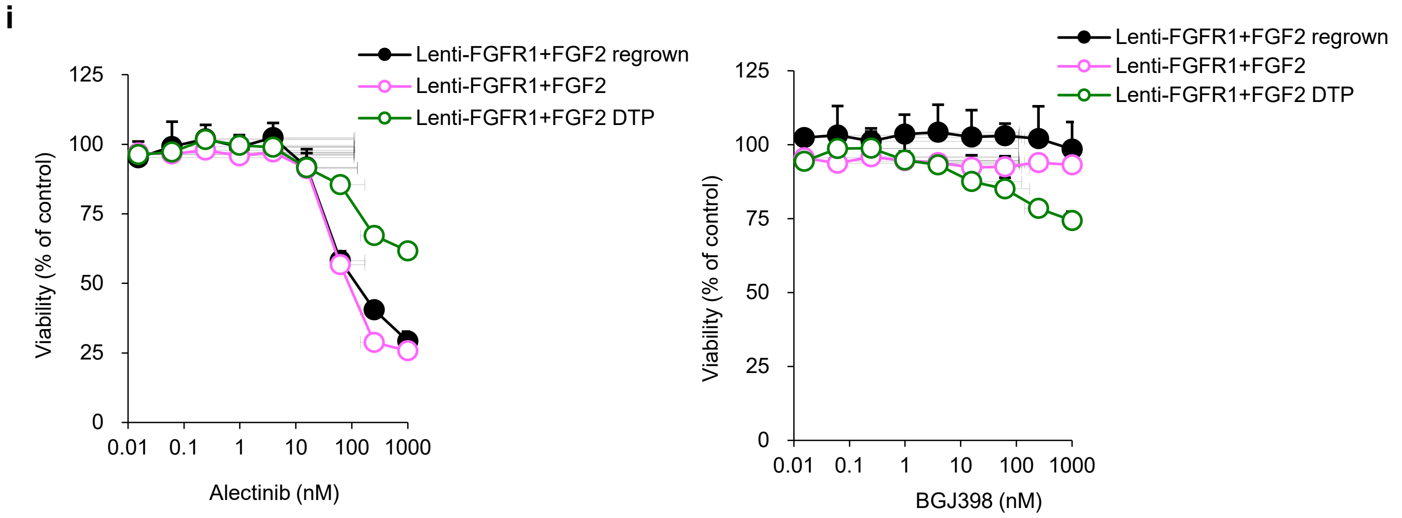

Cells	Alectinib (nM)	BGJ398 (nM)
Lenti-FGFR1+FGF2 regrown cells	149.3	>1000

j

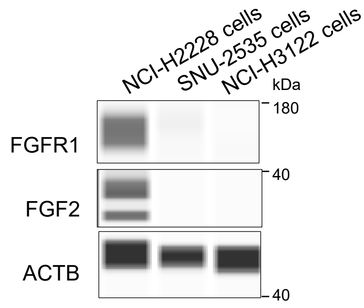

k

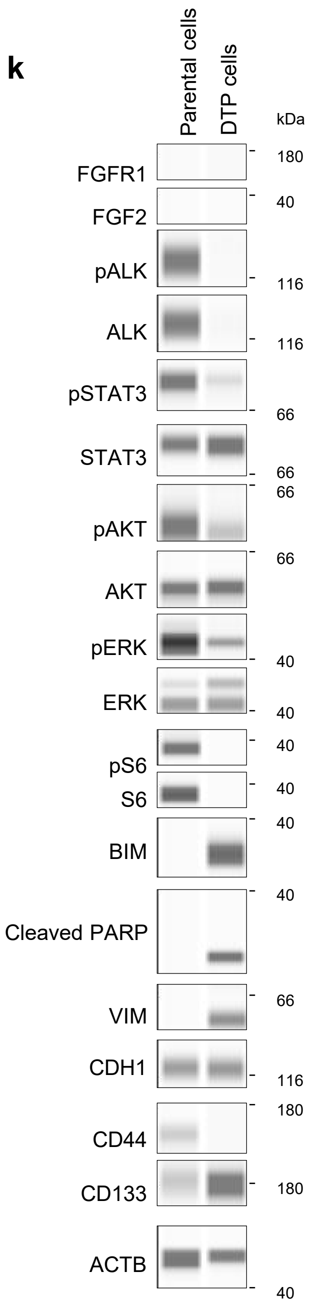

l

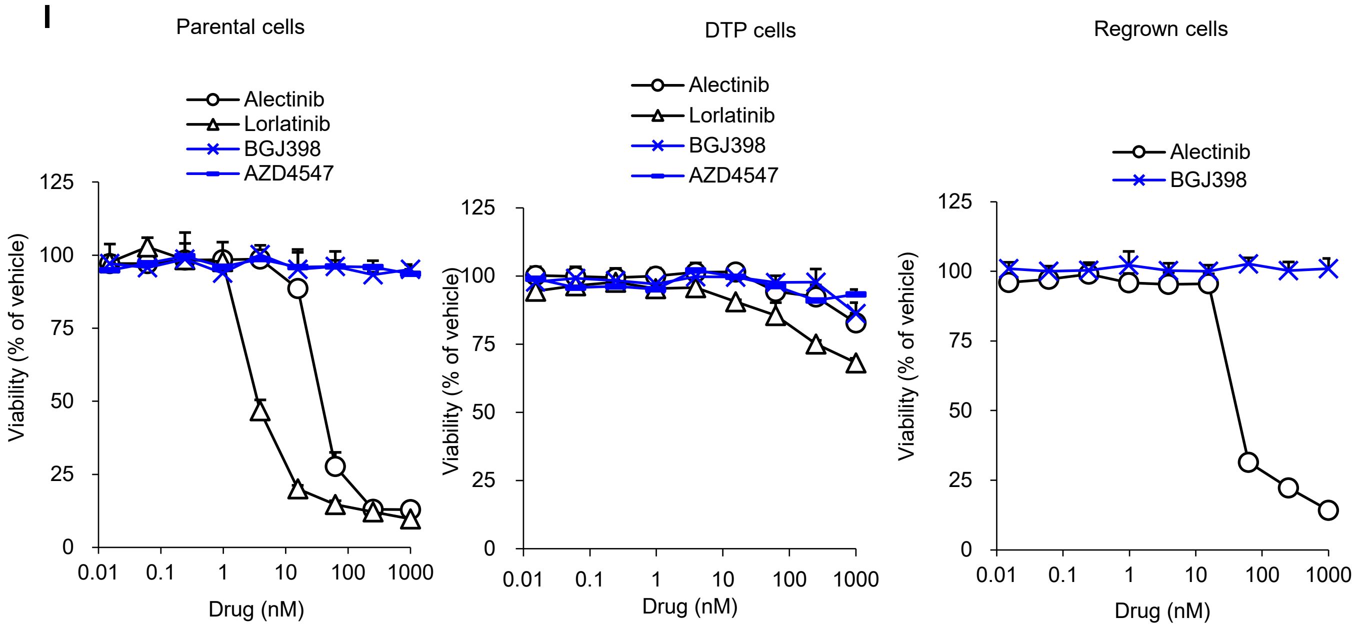

Cells	Alectinib (nM)	Lorlatinib (nM)	BGJ398 (nM)	AZD4547 (nM)
Parental cells	45.4	3.7	>1000	>1000
DTP cells	>1000	>1000	>1000	>1000
Regrown cells	48.9	ND	>1000	ND

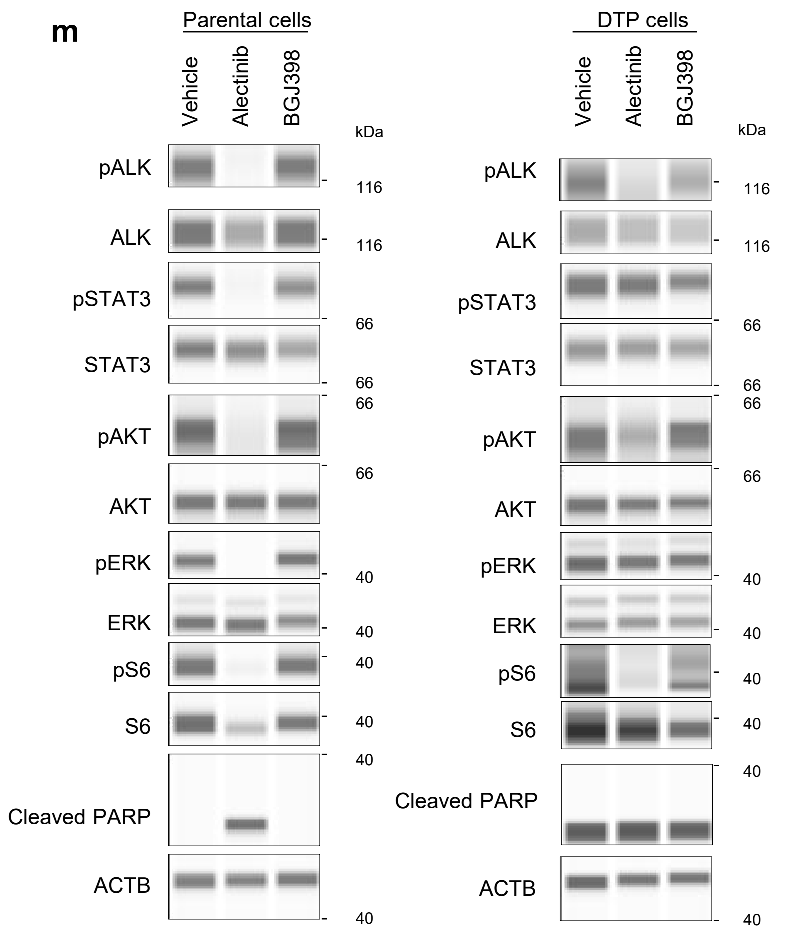

Supplementary Figure 4. Effect of FGFR1 or FGF2 on sensitivity to ALK inhibitors.

(**a**) Immunoblots of cell lysates described in Fig. 2a 2 days after transfection. (**b**) Immunoblots of cell lysates described in Fig. 2b. (**c**) NCI-H2228 parental, FGFR1-, or FGF2-knockout clone cells were cultured with alectinib for 2 days. Their caspase 3/7 activities in alectinib-treated versus vehicle-treated groups were measured. (**d**) NCI-H2228 parental, FGFR1-, or FGF2-knockout clone cells were cultured with lorlatinib for 8 days. (**e**) Lenti-control, lenti-FGF2, lenti-FGFR1+FGF2 SNU-2535 parental cells, and their DTP cells treated with 1000 nM alectinib for 13 days were cultured with alectinib, lorlatinib, BGJ398, or AZD4547 for 8 days. IC_{50} values of the ALK-TKIs, and IC_{20} values of the FGFR-TKIs are shown. (**f**) Immunoblots of these parental and DTP cells. (**g**) FGFR1 Phosphorylation levels of parental and DTP cells were measured by ELISA. Each point represents the mean + standard deviation of triplicate experiments. *$P < 0.05$ of lenti-control DTP, lenti-FGF2 DTP, and lenti-FGFR1+FGF2 cells versus lenti-FGFR1+FGF2 DTP cells in the Dunnett-test. (**h**) Immunoblots of cell lysates of these parental and DTP cells treated with 1000 nM alectinib, or 300 nM BGJ398 for 3 hours. (**i**) Lenti-FGFR1+FGF2 SNU-2535 parental, DTP, and regrown cells were cultured with alectinib, or BGJ398 for 8 days. IC_{50} values of the alectinib, and IC_{20} values of the BGJ398 are shown. (**j**) Immunoblots of cell lysates of NCI-H2228, SNU-2535, and NCI-H3122 cells. (**k**) Immunoblots of cell lysates of NCI-H3122 parental, and DTP cells. (**l**) NCI-H3122 parental, DTP, and regrown cells were cultured with alectinib, lorlatinib, BGJ398, or AZD4547 for 8 days. IC_{50} values of the alectinib and lorlatinib and IC_{20} values of the BGJ398 and AZD4547 are shown. (**m**) Immunoblots of cell lysates of NCI-H3122 parental, and DTP cells treated with 1000 nM alectinib, or 300 nM BGJ398 for 3 hours.

a

Covariate parameter	Variables
FGFR1 or FGF2 mRNA	High/Low
Treatment regimen	Alectinib / Crizotinib
ECOG PS	0/ 1
Treatment line	1/ 2
Disease status	Stage IIB or IV / Postoperative reccurence
Brain metastases at baseline by IRF	Yes/ No

b

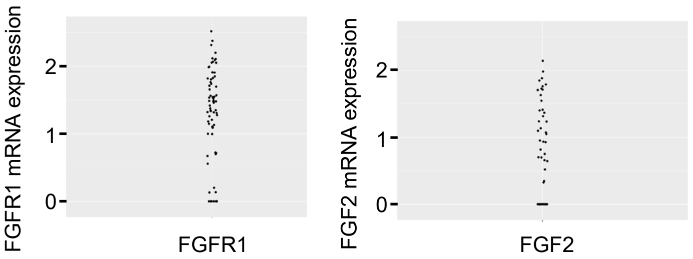

c

FGFR1

Subset	N (High)	N (Low)	Event (High)	Event (Low)
subset1	61	8	8	40
subset2	60	9	9	39
subset3	59	10	10	38
subset4	58	11	10	38
subset5	57	12	11	37
subset6	56	13	11	37
subset7	55	14	12	36
subset8	54	15	13	35
subset9	53	16	14	34
subset10	52	17	15	33
subset11	51	18	16	32
subset12	50	19	17	31
subset13	49	20	17	31
subset14	48	21	17	31
subset15	47	22	18	30
subset16	46	23	18	30
subset17	45	24	19	29
subset18	44	25	20	28
subset19	43	26	21	27
subset20	42	27	22	26
subset21	41	28	22	26
subset22	40	29	22	26
subset23	39	30	22	26
subset24	38	31	22	26
subset25	37	32	23	25
subset26	36	33	24	24
subset27	35	34	25	23
subset28	34	35	25	23
subset29	33	36	26	22
subset30	32	37	27	21
subset31	31	38	28	20
subset32	30	39	28	20
subset33	29	40	28	20
subset34	28	41	29	19
subset35	27	42	30	18
subset36	26	43	31	17
subset37	25	44	31	17
subset38	24	45	32	16
subset39	23	46	33	15
subset40	22	47	33	15
subset41	21	48	33	15
subset42	20	49	33	15
subset43	19	50	34	14
subset44	18	51	34	14
subset45	17	52	35	13
subset46	16	53	35	13
subset47	15	54	36	12
subset48	14	55	37	11
subset49	13	56	38	10
subset50	12	57	39	9
subset51	11	58	40	8
subset52	10	59	41	7
subset53	9	60	42	6
subset54	8	61	43	5
subset55	7	62	44	4

FGF2

Subset	N (High)	N (Low)	Event (High)	Event (Low)
subset1	32	37	26	22
subset2	31	38	27	21
subset3	30	39	27	21
subset4	29	40	28	20
subset5	28	41	28	20
subset6	27	42	29	19
subset7	26	43	30	18
subset8	25	44	31	17
subset9	24	45	32	16
subset10	23	46	32	16
subset11	22	47	33	15
subset12	21	48	33	15
subset13	20	49	33	15
subset14	19	50	33	15
subset15	18	51	33	15
subset16	17	52	33	15
subset17	16	53	34	14
subset18	15	54	35	13
subset19	14	55	36	12
subset20	13	56	36	12
subset21	12	57	37	11
subset22	11	58	38	10
subset23	10	59	39	9
subset24	9	60	40	8
subset25	8	61	40	8
subset26	7	62	41	7

d

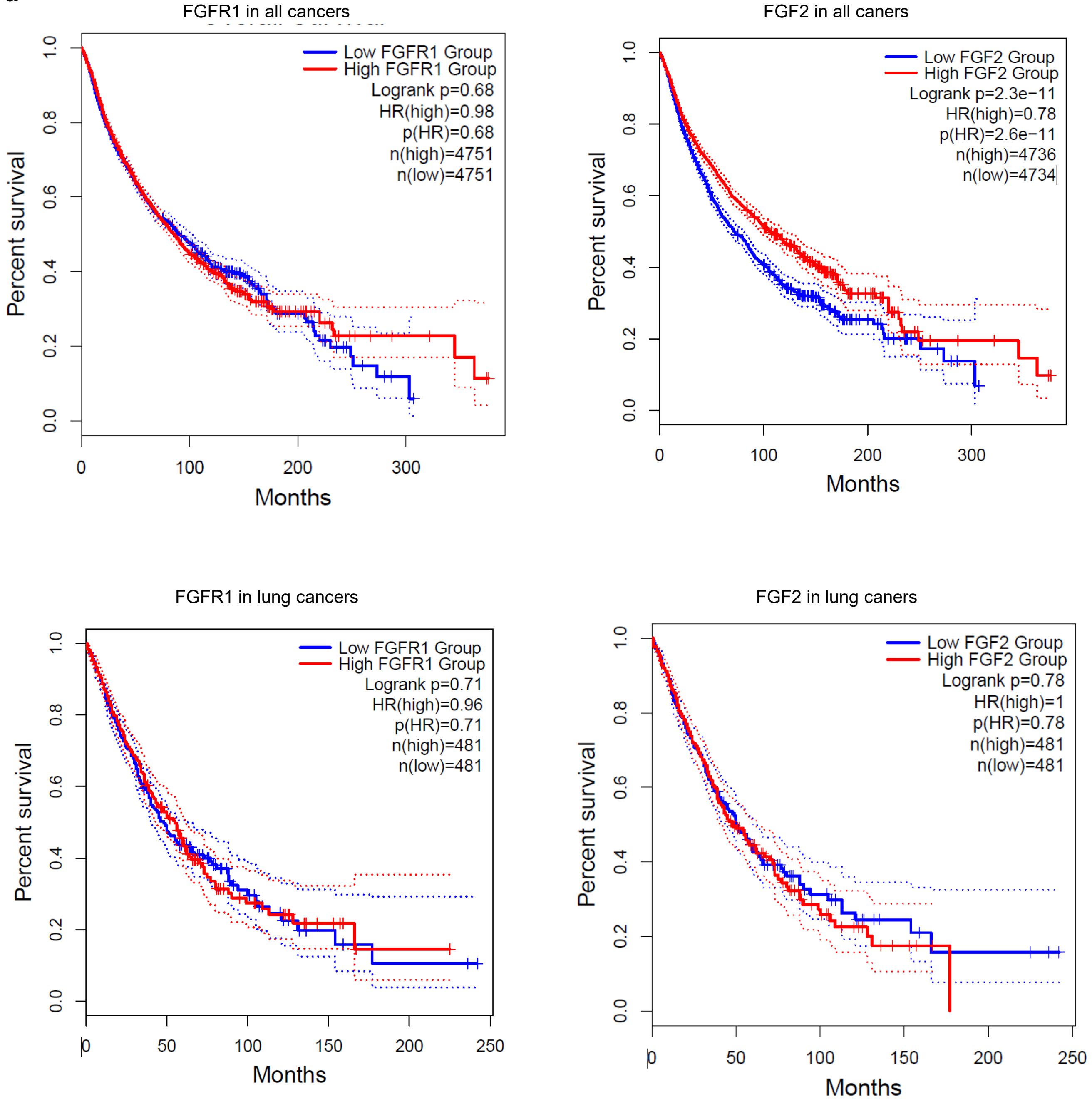

Supplementary Figure 5. Impact of FGFR1 or FGF2 expression on the response to ALK inhibitors in ALK+NSCLC patients.

(**a**) Clinical covariates for multivariate Cox proportional hazard model. (**b**) Plot of FGFR1 or FGF2 mRNA expression level (log_2(TPM+1)) for each patient in J-ALEX study. (**c**) Number of patients and events in each subset of FGFR1 or FGF2 mRNA expression was shown. (**d**) Kaplan–Meier curves comparing overall survival between patients with high vs. low mRNA expression of FGFR1 or FGF2 in was plotted for lung adenocarcinoma and lung squamous cell carcinoma or all cancer types by GEPIA2 database (http://gepia2.cancer-pku.cn.).

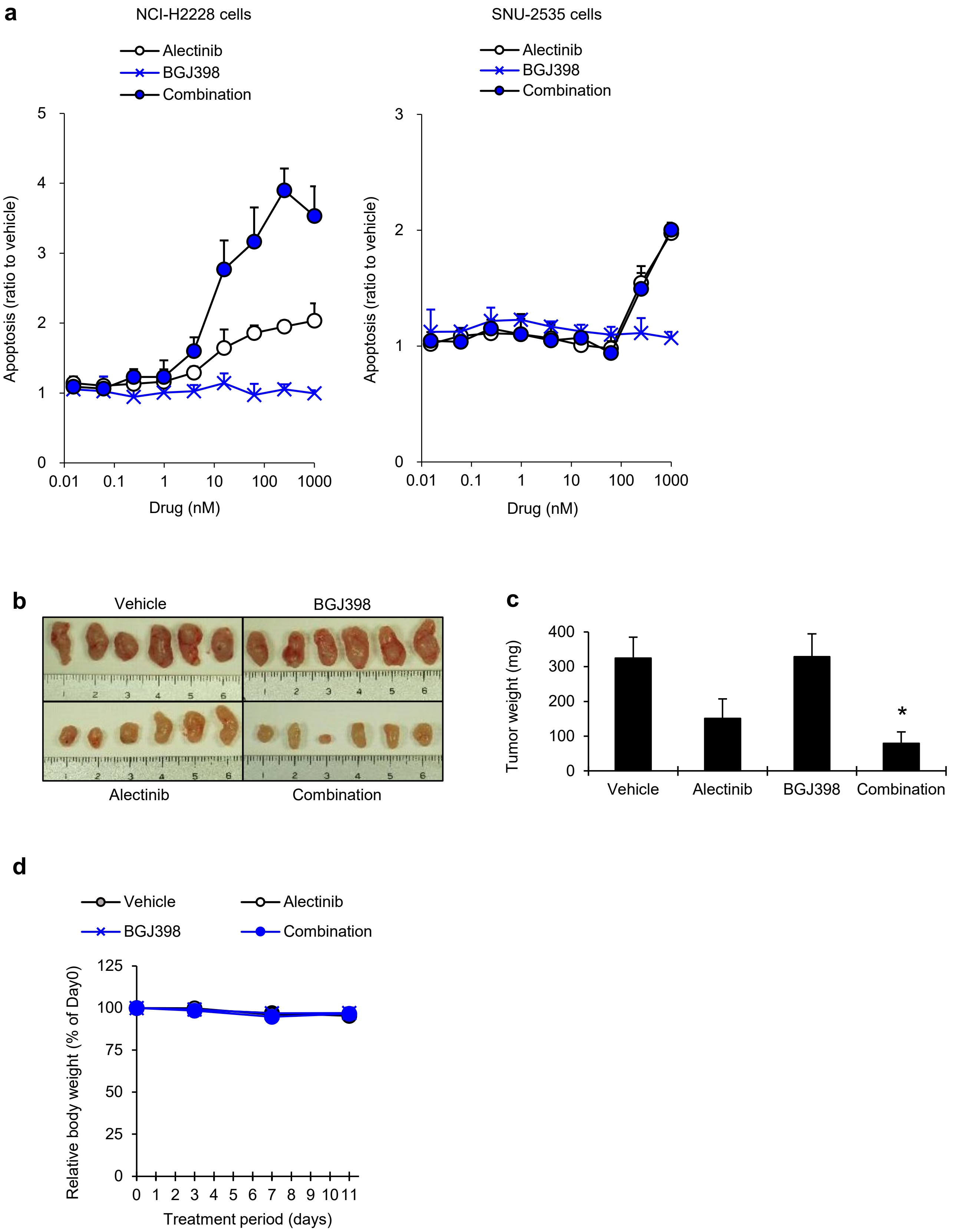

Supplementary Figure 6. FGFR inhibition enhances the effect of ALK inhibitor on $FGFR1^{high}$ and $FGF2^{high}$ cells.

(**a**) Cells were cultured with alectinib, BGJ398, or alectinib in combination with 300 nM BGJ398 for 2 days, and the caspase 3/7 activity was measured. (**b**) NCI-H2228 tumors excised from mice on Day 11. (**c**) Tumor weight was measured. Each bar represents the mean + SD. **P* < 0.05 versus alectinib or BGJ398; the Wilcoxon rank sum test by the Holm–Bonferroni method. (**d**) Body weight change of mice relative to the start of the treatment.

a

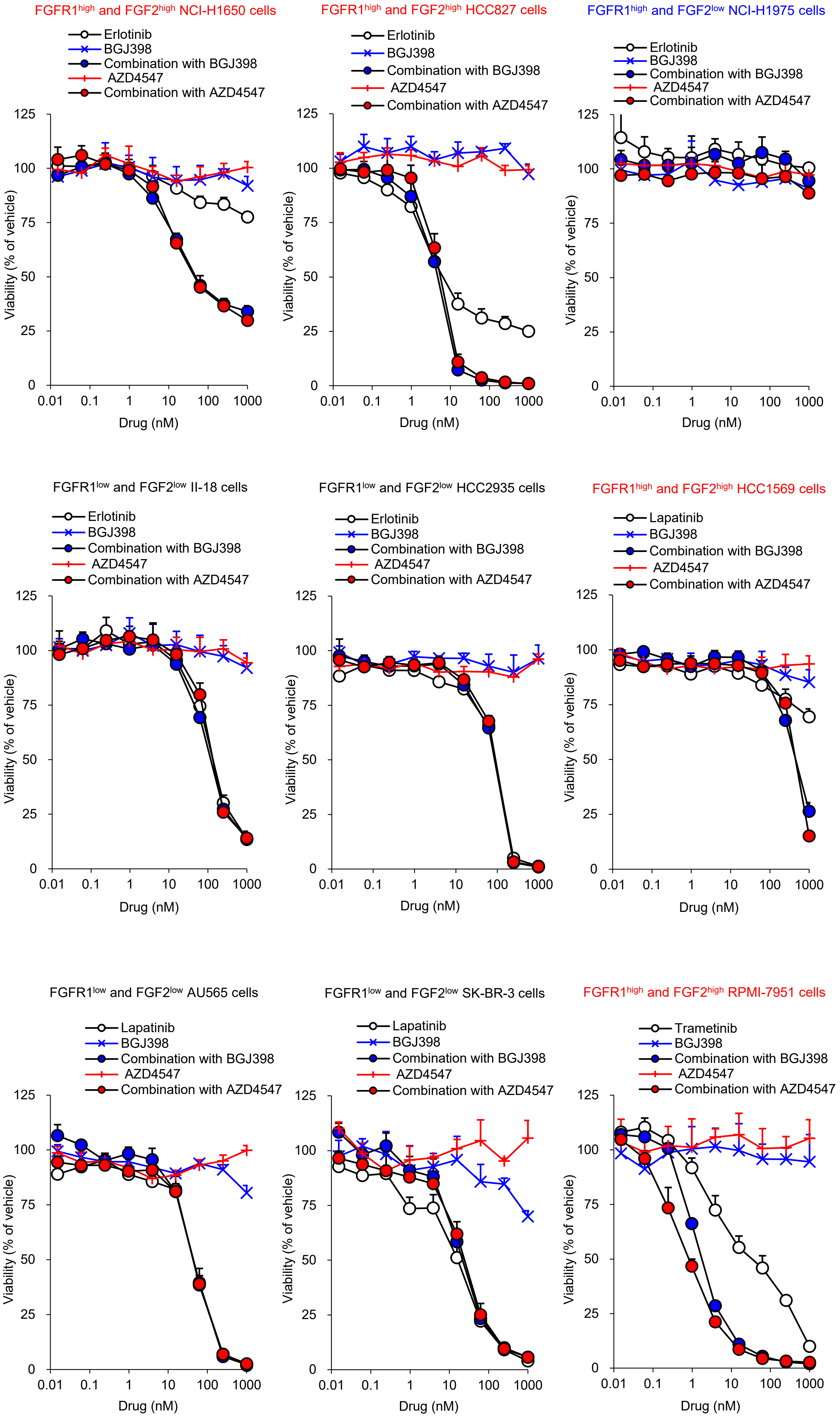
FGFR1high and FGF2high NCI-H1650 cells
Erlotinib
BGJ398
Combination with BGJ398
AZD4547
Combination with AZD4547
Viability (% of vehicle)
Drug (nM)
FGFR1high and FGF2high HCC827 cells
Erlotinib
BGJ398
Combination with BGJ398
AZD4547
Combination with AZD4547
Viability (% of vehicle)
Drug (nM)
FGFR1high and FGF2low NCI-H1975 cells
Erlotinib
BGJ398
Combination with BGJ398
AZD4547
Combination with AZD4547
Viability (% of vehicle)
Drug (nM)
FGFR1low and FGF2low II-18 cells
Erlotinib
BGJ398
Combination with BGJ398
AZD4547
Combination with AZD4547
Viability (% of vehicle)
Drug (nM)
FGFR1low and FGF2low HCC2935 cells
Erlotinib
BGJ398
Combination with BGJ398
AZD4547
Combination with AZD4547
Viability (% of vehicle)
Drug (nM)
FGFR1high and FGF2high HCC1569 cells
Lapatinib
BGJ398
Combination with BGJ398
AZD4547
Combination with AZD4547
Viability (% of vehicle)
Drug (nM)
FGFR1low and FGF2low AU565 cells
Lapatinib
BGJ398
Combination with BGJ398
AZD4547
Combination with AZD4547
Viability (% of vehicle)
Drug (nM)
FGFR1low and FGF2low SK-BR-3 cells
Lapatinib
BGJ398
Combination with BGJ398
AZD4547
Combination with AZD4547
Viability (% of vehicle)
Drug (nM)
FGFR1high and FGF2high RPMI-7951 cells
Trametinib
BGJ398
Combination with BGJ398
AZD4547
Combination with AZD4547
Viability (% of vehicle)
Drug (nM)
0
25
50
75
100
125
0.01
0.1
1
10
100
1000

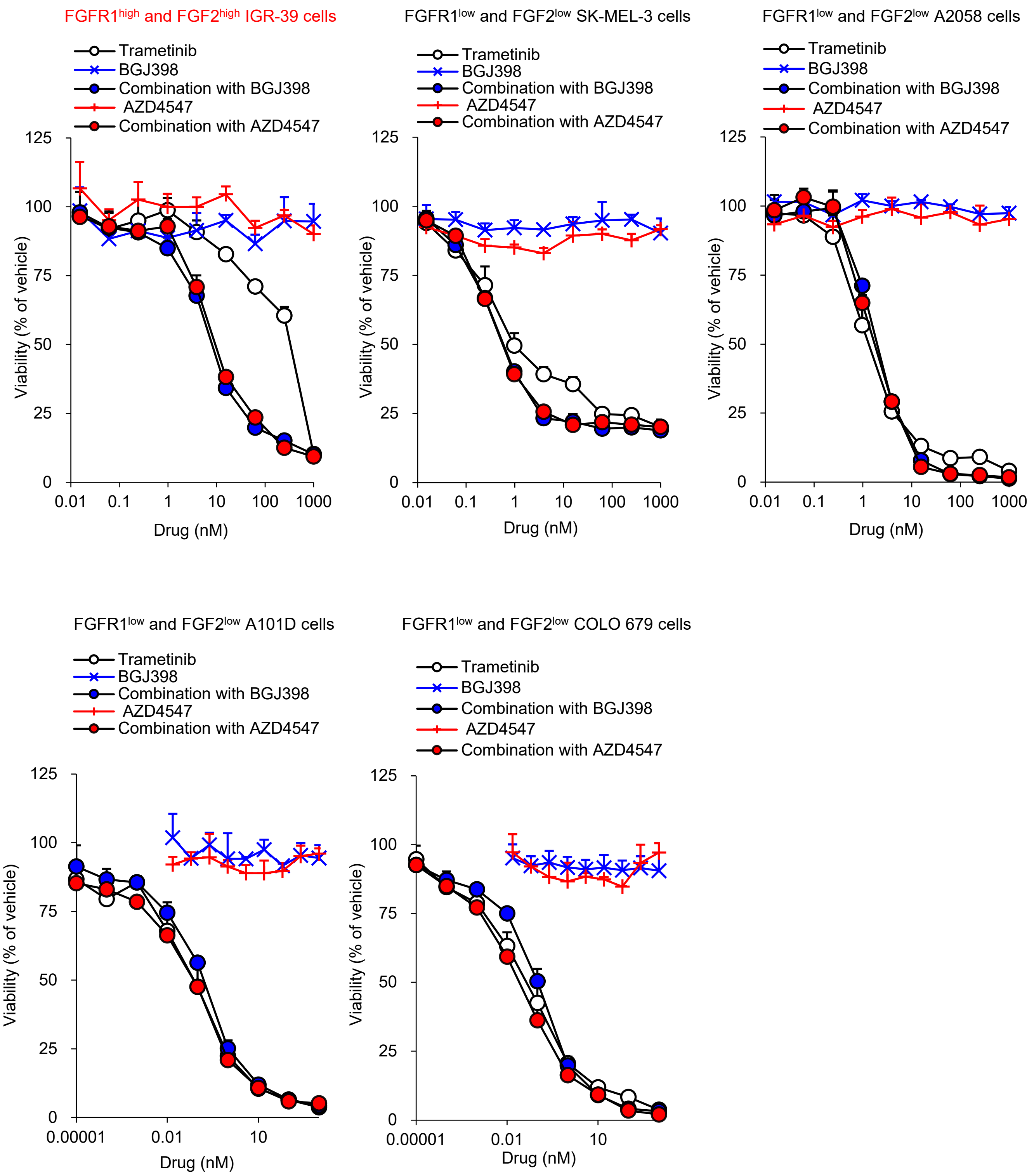
FGFR1^{high} and FGF2^{high} IGR-39 cells
FGFR1^{low} and FGF2^{low} SK-MEL-3 cells
FGFR1^{low} and FGF2^{low} A2058 cells
Trametinib
BGJ398
Combination with BGJ398
AZD4547
Combination with AZD4547
Viability (% of vehicle)
125
100
75
50
25
0
0.01
0.1
1
10
100
1000
Drug (nM)
FGFR1^{low} and FGF2^{low} A101D cells
FGFR1^{low} and FGF2^{low} COLO 679 cells
0.00001
0.01
10

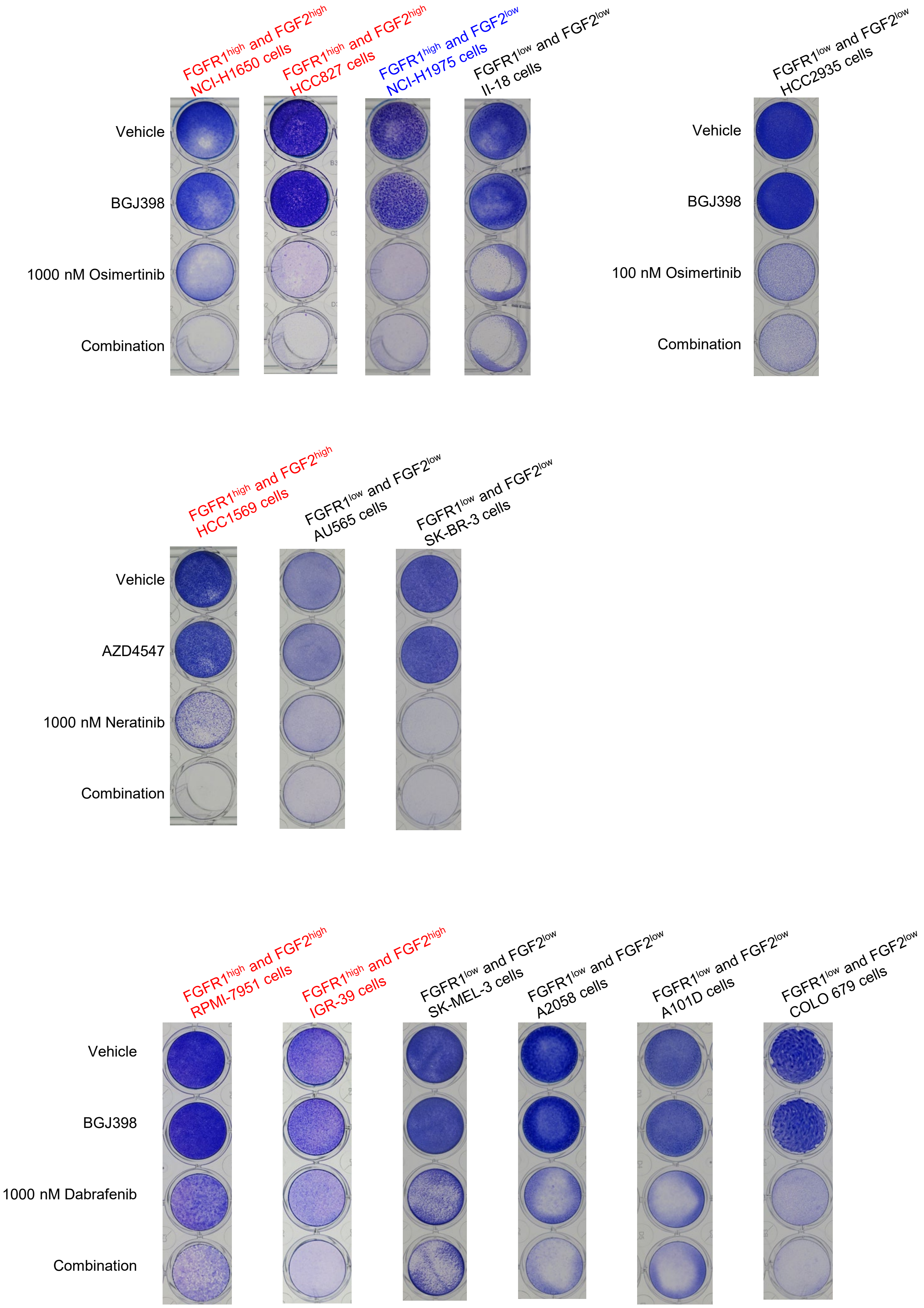

FGFR1^{high} and FGF2^{high}
NCI-H1650 cells
FGFR1^{high} and FGF2^{high}
HCC827 cells
FGFR1^{high} and FGF2^{low}
NCI-H1975 cells
FGFR1^{low} and FGF2^{low}
II-18 cells
FGFR1^{low} and FGF2^{low}
HCC2935 cells
Vehicle
BGJ398
1000 nM Osimertinib
Combination
Vehicle
BGJ398
100 nM Osimertinib
Combination
FGFR1^{high} and FGF2^{high}
HCC1569 cells
FGFR1^{low} and FGF2^{low}
AU565 cells
FGFR1^{low} and FGF2^{low}
SK-BR-3 cells
Vehicle
AZD4547
1000 nM Neratinib
Combination
FGFR1^{high} and FGF2^{high}
RPMI-7951 cells
FGFR1^{high} and FGF2^{high}
IGR-39 cells
FGFR1^{low} and FGF2^{low}
SK-MEL-3 cells
FGFR1^{low} and FGF2^{low}
A2058 cells
FGFR1^{low} and FGF2^{low}
A101D cells
FGFR1^{low} and FGF2^{low}
COLO 679 cells
Vehicle
BGJ398
1000 nM Dabrafenib
Combination

c

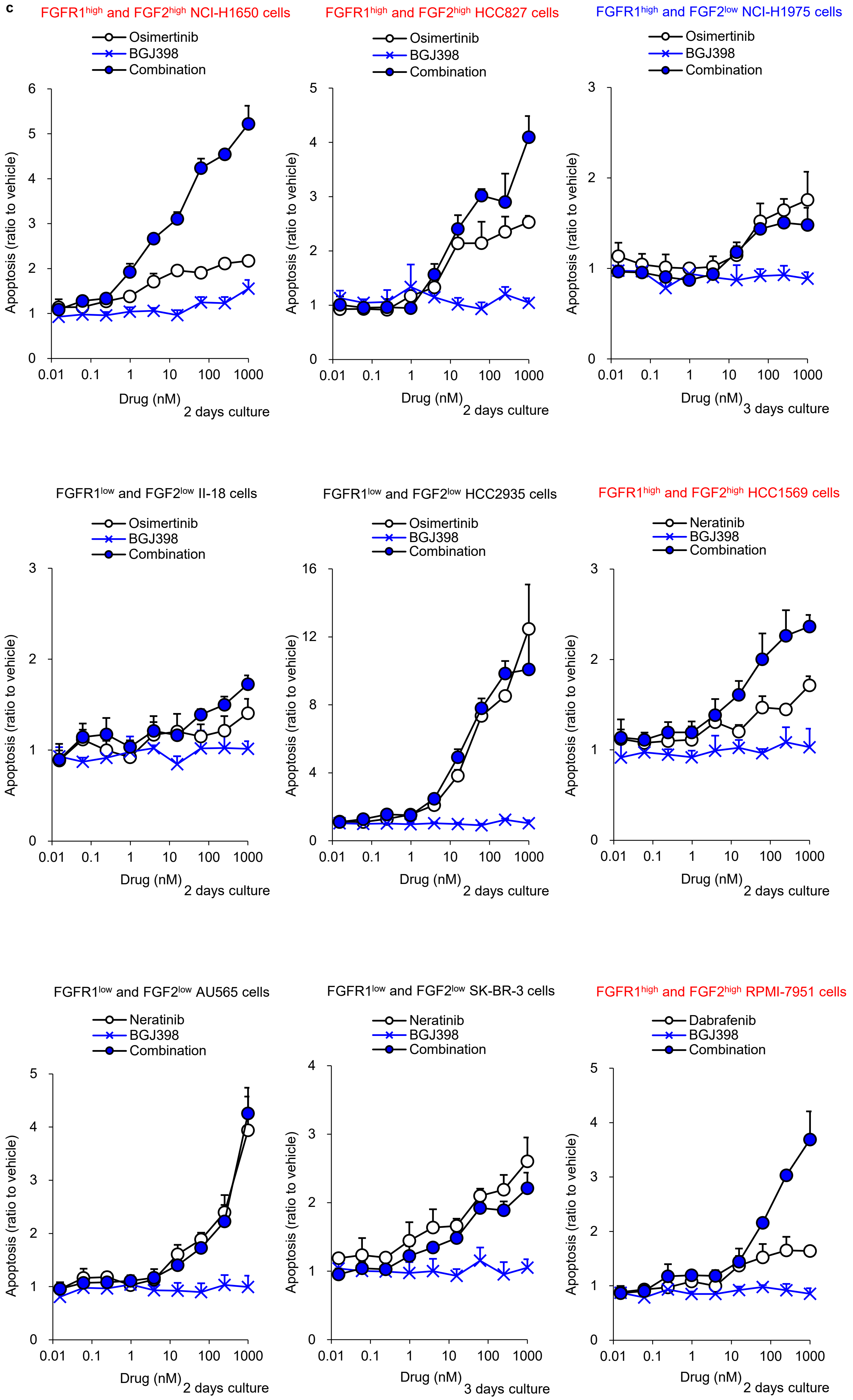
FGFR1^{high} and FGF2^{high} NCI-H1650 cells
Osimertinib
BGJ398
Combination
Apoptosis (ratio to vehicle)
Drug (nM)
2 days culture
FGFR1^{high} and FGF2^{high} HCC827 cells
Osimertinib
BGJ398
Combination
Apoptosis (ratio to vehicle)
Drug (nM)
2 days culture
FGFR1^{high} and FGF2^{low} NCI-H1975 cells
Osimertinib
BGJ398
Combination
Apoptosis (ratio to vehicle)
Drug (nM)
3 days culture
FGFR1^{low} and FGF2^{low} II-18 cells
Osimertinib
BGJ398
Combination
Apoptosis (ratio to vehicle)
Drug (nM)
2 days culture
FGFR1^{low} and FGF2^{low} HCC2935 cells
Osimertinib
BGJ398
Combination
Apoptosis (ratio to vehicle)
Drug (nM)
2 days culture
FGFR1^{high} and FGF2^{high} HCC1569 cells
Neratinib
BGJ398
Combination
Apoptosis (ratio to vehicle)
Drug (nM)
2 days culture
FGFR1^{low} and FGF2^{low} AU565 cells
Neratinib
BGJ398
Combination
Apoptosis (ratio to vehicle)
Drug (nM)
2 days culture
FGFR1^{low} and FGF2^{low} SK-BR-3 cells
Neratinib
BGJ398
Combination
Apoptosis (ratio to vehicle)
Drug (nM)
3 days culture
FGFR1^{high} and FGF2^{high} RPMI-7951 cells
Dabrafenib
BGJ398
Combination
Apoptosis (ratio to vehicle)
Drug (nM)
2 days culture

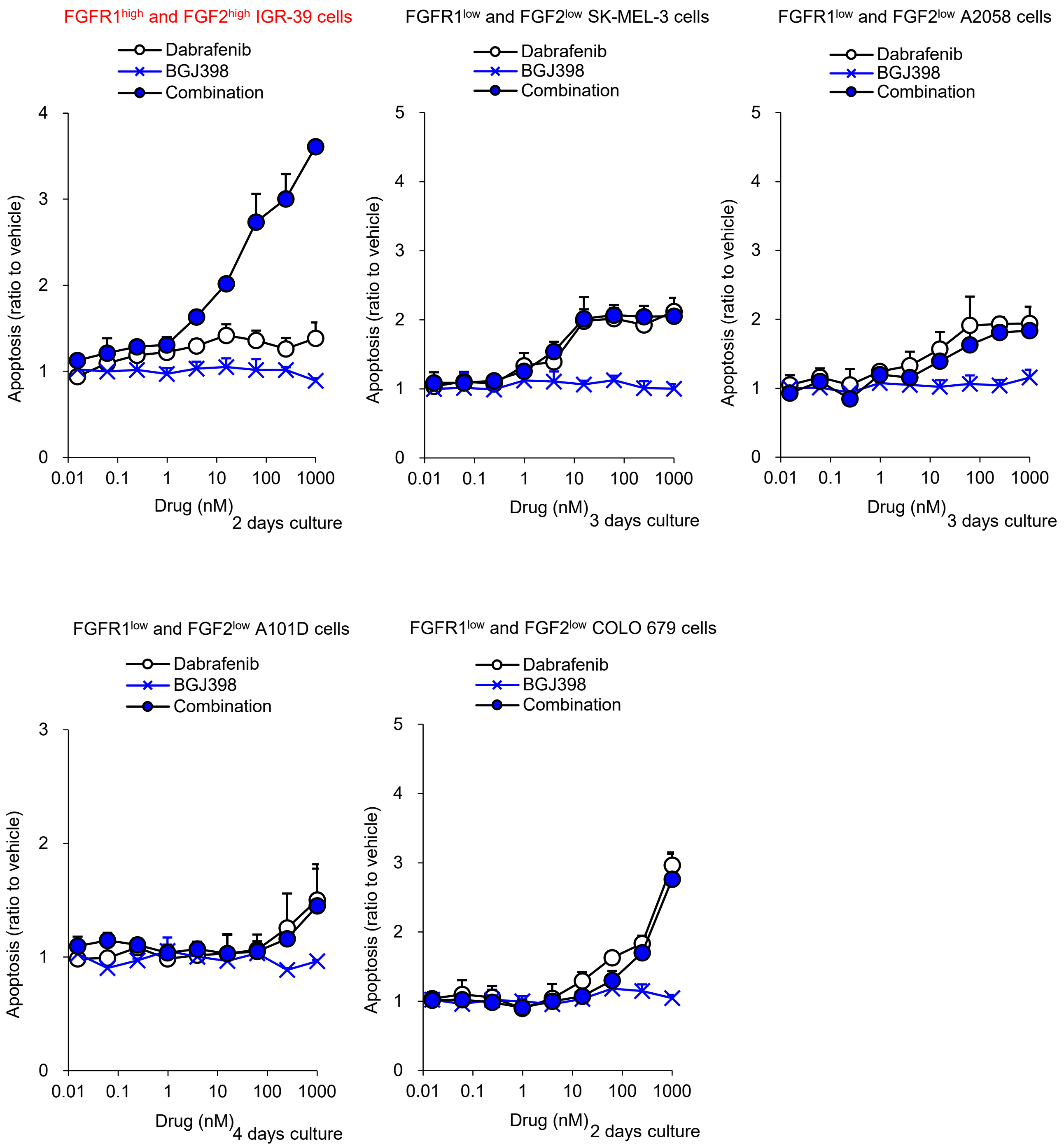
FGFR1high and FGF2high IGR-39 cells
Dabrafenib
BGJ398
Combination
Apoptosis (ratio to vehicle)
Drug (nM)
2 days culture
FGFR1low and FGF2low SK-MEL-3 cells
3 days culture
FGFR1low and FGF2low A2058 cells
3 days culture
FGFR1low and FGF2low A101D cells
4 days culture
FGFR1low and FGF2low COLO 679 cells
2 days culture

d

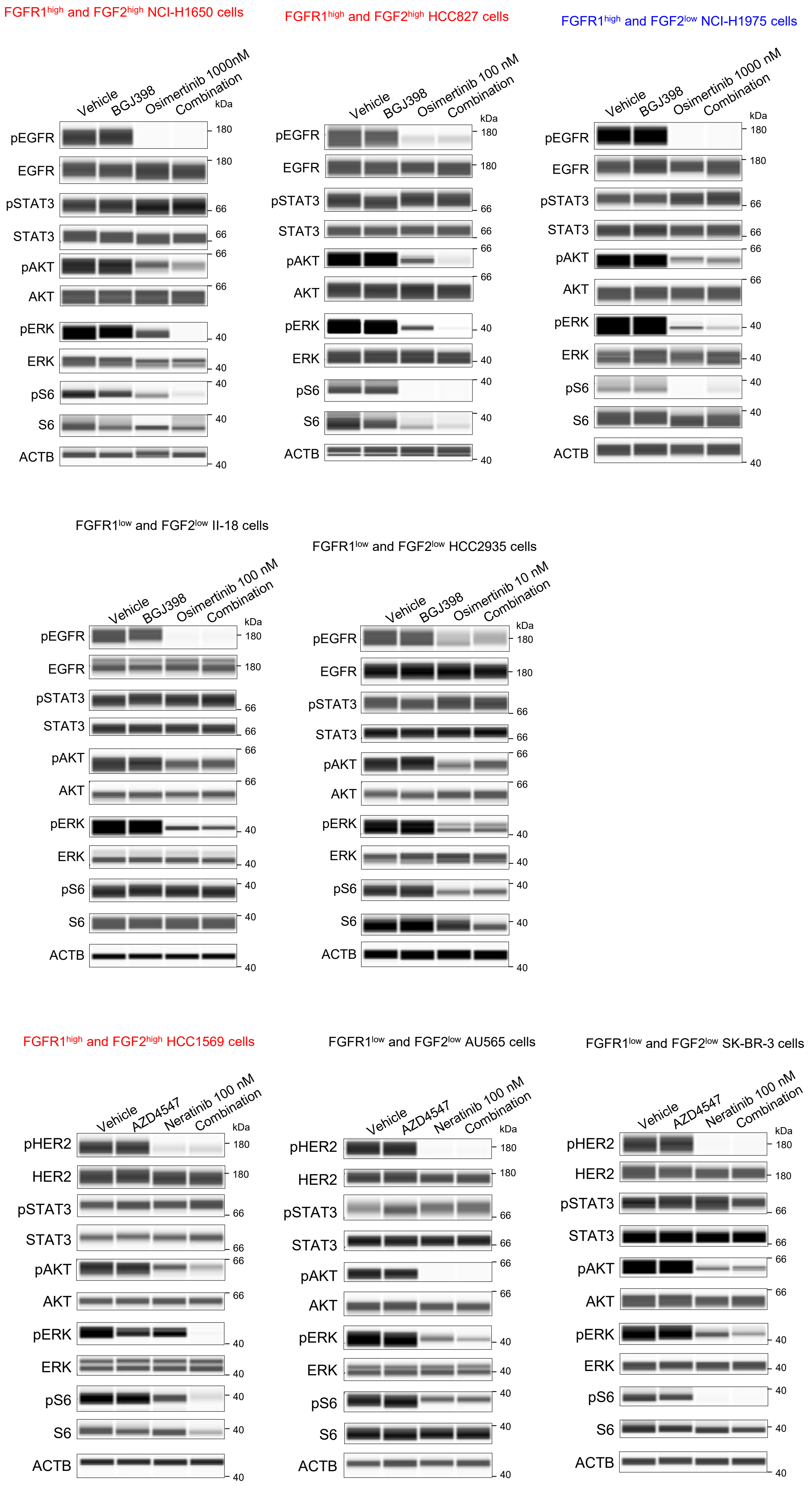
FGFR1^{high} and FGF2^{high} NCI-H1650 cells
Vehicle
BGJ398
Osimertinib 1000nM
Combination
kDa
pEGFR
EGFR
pSTAT3
STAT3
pAKT
AKT
pERK
ERK
pS6
S6
ACTB
180
66
40
FGFR1^{high} and FGF2^{high} HCC827 cells
Vehicle
BGJ398
Osimertinib 100 nM
Combination
FGFR1^{high} and FGF2^{low} NCI-H1975 cells
Vehicle
BGJ398
Osimertinib 1000 nM
Combination
FGFR1^{low} and FGF2^{low} II-18 cells
Vehicle
BGJ398
Osimertinib 100 nM
Combination
FGFR1^{low} and FGF2^{low} HCC2935 cells
Vehicle
BGJ398
Osimertinib 10 nM
Combination
FGFR1^{high} and FGF2^{high} HCC1569 cells
Vehicle
AZD4547
Neratinib 100 nM
Combination
pHER2
HER2
FGFR1^{low} and FGF2^{low} AU565 cells
Vehicle
AZD4547
Neratinib 100 nM
Combination
FGFR1^{low} and FGF2^{low} SK-BR-3 cells
Vehicle
AZD4547
Neratinib 100 nM
Combination

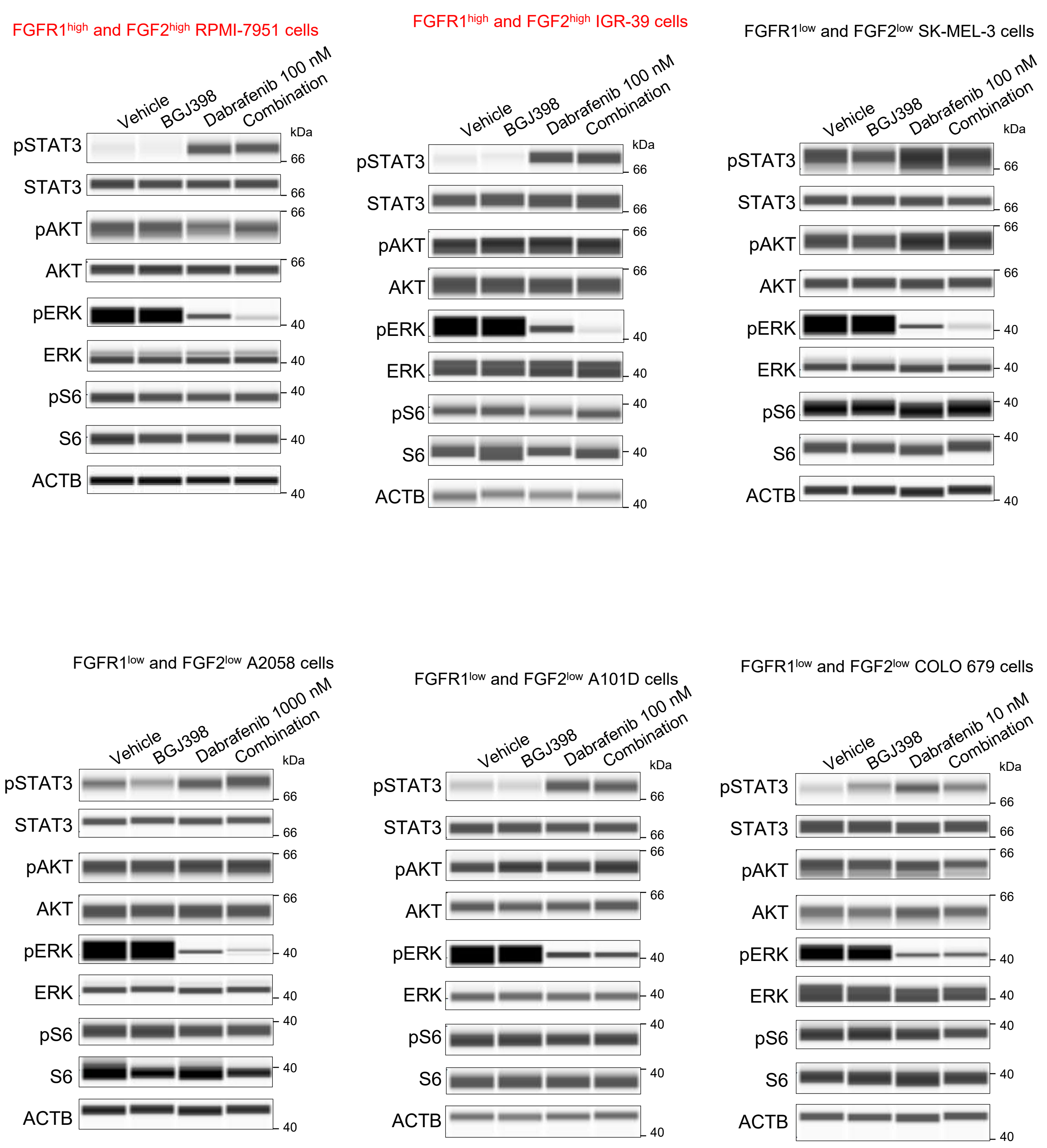
FGFR1^{high} and FGF2^{high} RPMI-7951 cells
Vehicle
BGJ398
Dabrafenib 100 nM
Combination
kDa
pSTAT3
STAT3
pAKT
AKT
pERK
ERK
pS6
S6
ACTB
66
40
FGFR1^{high} and FGF2^{high} IGR-39 cells
Vehicle
BGJ398
Dabrafenib 100 nM
Combination
FGFR1^{low} and FGF2^{low} SK-MEL-3 cells
Vehicle
BGJ398
Dabrafenib 100 nM
Combination
FGFR1^{low} and FGF2^{low} A2058 cells
Vehicle
BGJ398
Dabrafenib 1000 nM
Combination
FGFR1^{low} and FGF2^{low} A101D cells
Vehicle
BGJ398
Dabrafenib 100 nM
Combination
FGFR1^{low} and FGF2^{low} COLO 679 cells
Vehicle
BGJ398
Dabrafenib 10 nM
Combination

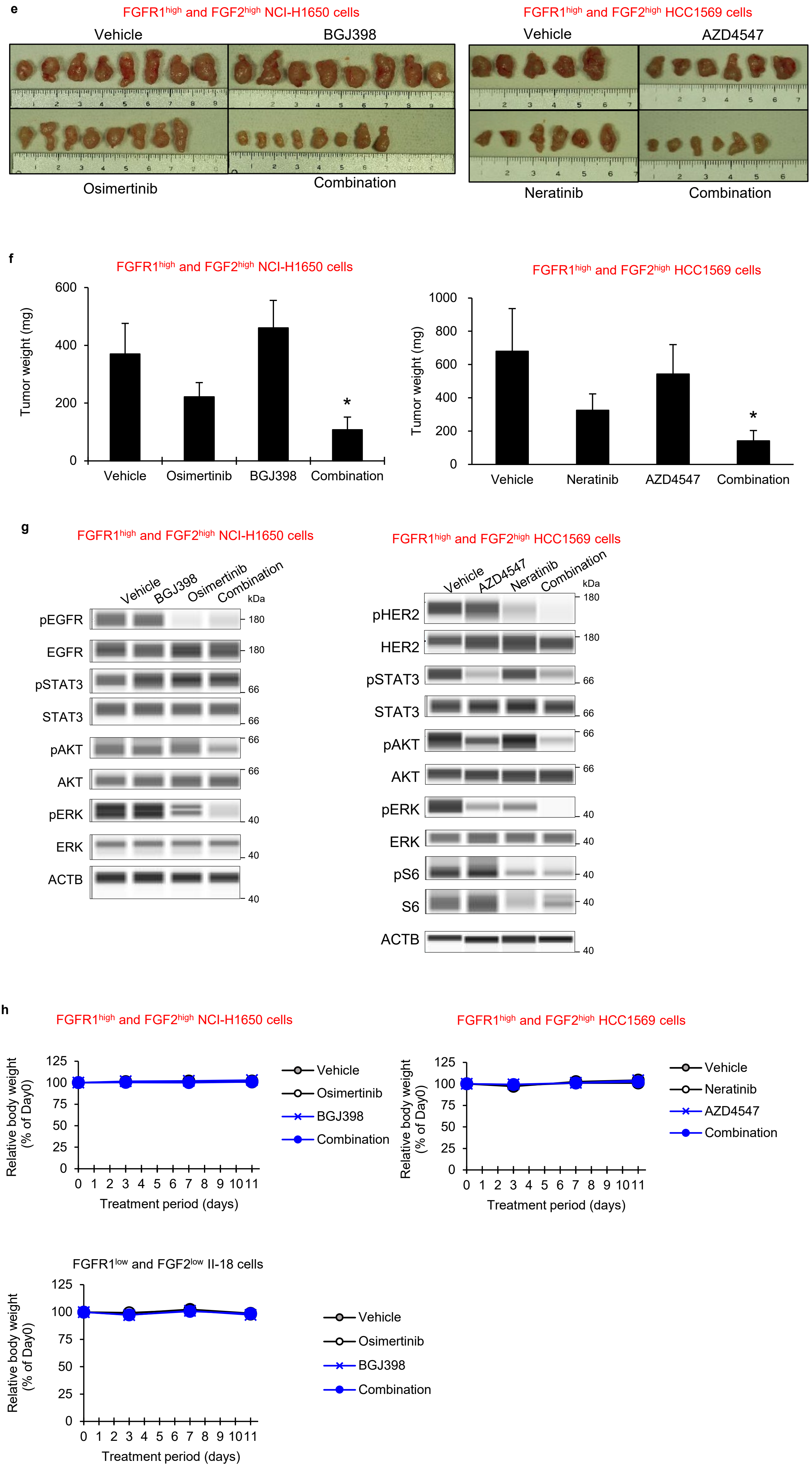
e
FGFR1high and FGF2high NCI-H1650 cells
Vehicle
BGJ398
Osimertinib
Combination
FGFR1high and FGF2high HCC1569 cells
Vehicle
AZD4547
Neratinib
Combination
f
FGFR1high and FGF2high NCI-H1650 cells
Tumor weight (mg)
600
400
200
0
Vehicle
Osimertinib
BGJ398
Combination
*
FGFR1high and FGF2high HCC1569 cells
Tumor weight (mg)
1000
800
600
400
200
0
Vehicle
Neratinib
AZD4547
Combination
*
g
FGFR1high and FGF2high NCI-H1650 cells
Vehicle
BGJ398
Osimertinib
Combination
kDa
pEGFR
EGFR
pSTAT3
STAT3
pAKT
AKT
pERK
ERK
ACTB
180
66
40
FGFR1high and FGF2high HCC1569 cells
Vehicle
AZD4547
Neratinib
Combination
kDa
pHER2
HER2
pSTAT3
STAT3
pAKT
AKT
pERK
ERK
pS6
S6
ACTB
180
66
40
h
FGFR1high and FGF2high NCI-H1650 cells
Relative body weight (% of Day0)
125
100
75
50
25
0
0 1 2 3 4 5 6 7 8 9 10 11
Treatment period (days)
Vehicle
Osimertinib
BGJ398
Combination
FGFR1high and FGF2high HCC1569 cells
Relative body weight (% of Day0)
125
100
75
50
25
0
0 1 2 3 4 5 6 7 8 9 10 11
Treatment period (days)
Vehicle
Neratinib
AZD4547
Combination
FGFR1low and FGF2low II-18 cells
Relative body weight (% of Day0)
125
100
75
50
25
0
0 1 2 3 4 5 6 7 8 9 10 11
Treatment period (days)
Vehicle
Osimertinib
BGJ398
Combination

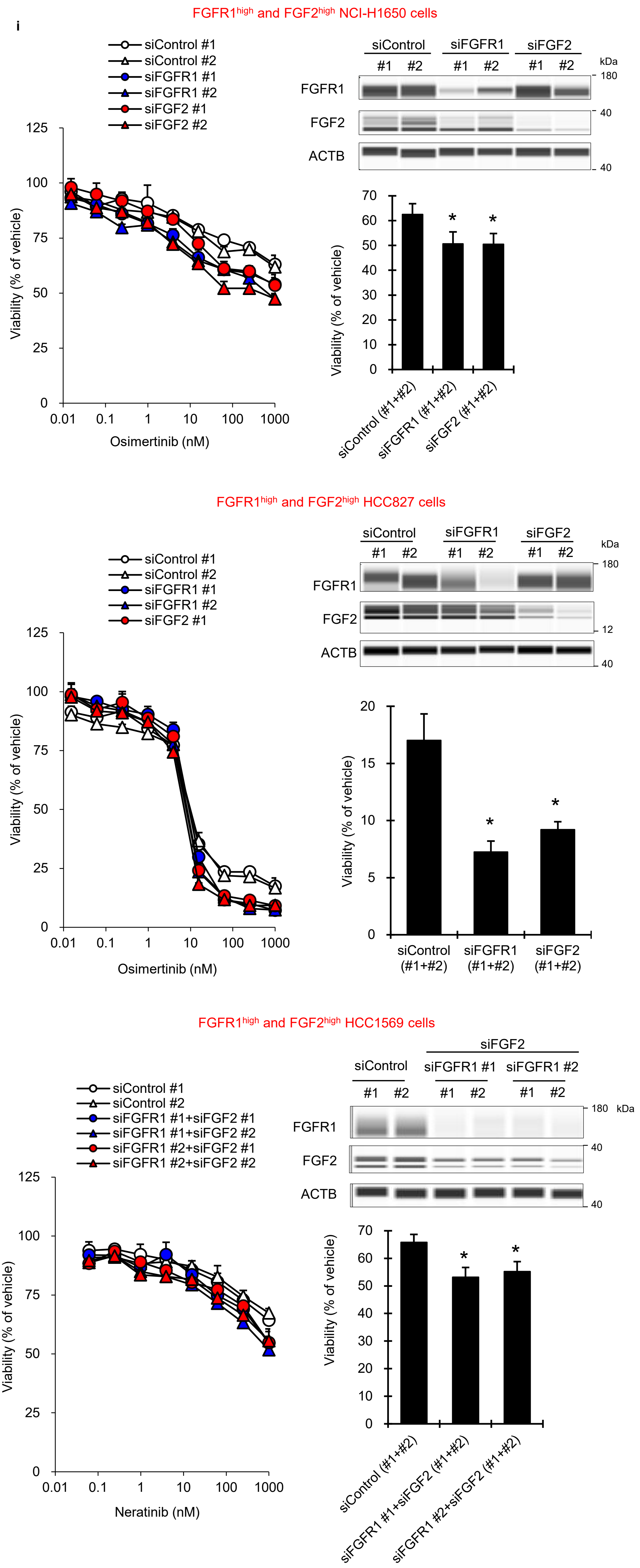
i
FGFR1high and FGF2high NCI-H1650 cells
siControl #1
siControl #2
siFGFR1 #1
siFGFR1 #2
siFGF2 #1
siFGF2 #2
Viability (% of vehicle)
125
100
75
50
25
0
0.01
0.1
1
10
100
1000
Osimertinib (nM)
siControl
siFGFR1
siFGF2
#1
#2
kDa
180
40
FGFR1
FGF2
ACTB
70
60
50
40
30
20
10
*
siControl (#1+#2)
siFGFR1 (#1+#2)
siFGF2 (#1+#2)
FGFR1high and FGF2high HCC827 cells
12
20
15
5
FGFR1high and FGF2high HCC1569 cells
siFGFR1 #1+siFGF2 #1
siFGFR1 #1+siFGF2 #2
siFGFR1 #2+siFGF2 #1
siFGFR1 #2+siFGF2 #2
Neratinib (nM)
siFGFR1 #1
siFGFR1 #2
siFGFR1 #1+siFGF2 (#1+#2)
siFGFR1 #2+siFGF2 (#1+#2)

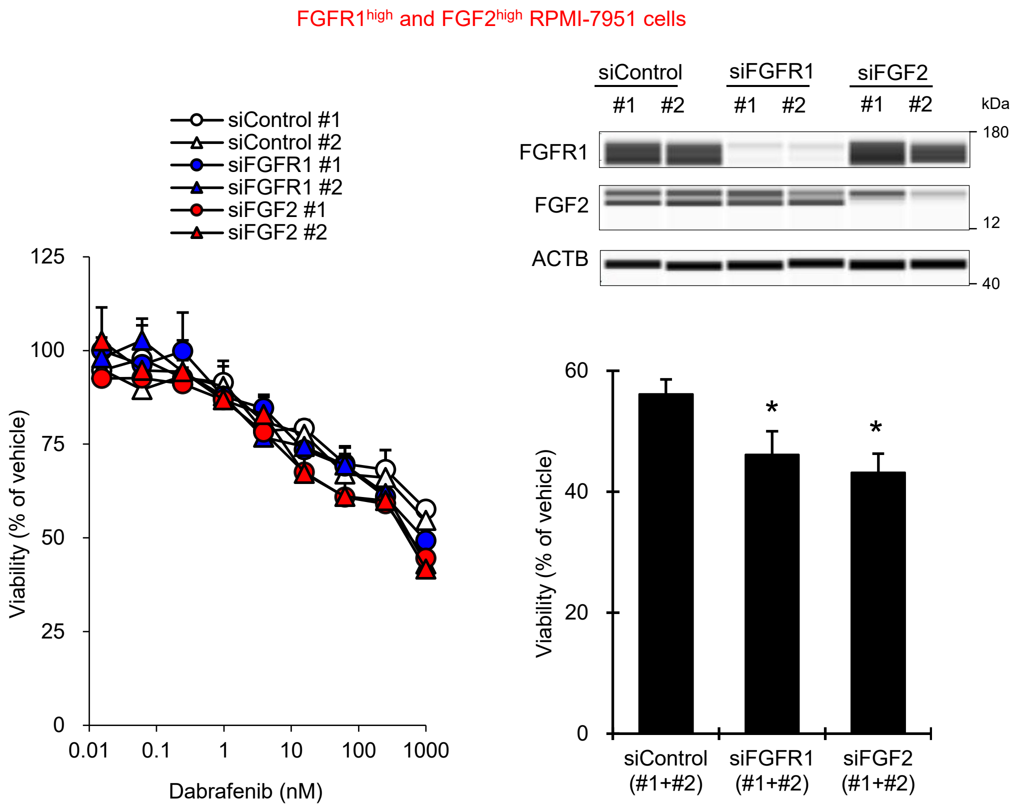

Supplementary Figure 7. Effects of combination with FGFR inhibitors on EGFR-, HER2-, BRAF-mutant cells.

(**a**) Cells were cultured with erlotinib, lapatinib, trametinib, BGJ398, AZD4547, or erlotinib, lapatinib, trametinib in combination with 300 nM of BGJ398 or AZD4547 for 8 days. (**b**) Crystal violet cell growth assays treated with osimertinib, neratinib, dabrafenib, FGFR-TKIs (300 nM BGJ398 or 300 nM AZD4547) or the combination of both drugs 8 days. (**c**) Cells were cultured with osimertinib, neratinib, trametinib, BGJ398, AZD4547, or osimertinib, neratinib, trametinib in combination with 300 nM of BGJ398 or AZD4547 for the indicated days. Their caspase 3/7 activities with treatments relative to their caspase 3/7 activities with vehicle were measured. (**d**) Immunoblots of cell lysates treated with osimertinib (NCI-H1650, NCI-H1975, HCC827, II-18, HCC2935), dabrafenib (A2058, RPMI-7951, IGR-39, SK-MEL-3, A101D, COLO 679), or 300 nM BGJ398 or the combination of both drugs for 3 hours. Immunoblots of cell lysates treated with neratinib (HCC1569, AU565, SK-BR-3), 300 nM AZD4547, or the combination of both drugs for 3 hours. (**e**) Mice bearing xenograft tumors were treated with vehicle, 5 mg/kg osimertinib, 20 mg/kg neratinib, 50 mg/kg BGJ398, 10 mg/kg AZD4547 or the combination of both drugs for 11 days. Tumors excised from mice on Day 11. (**f**) Tumor weight was measured. Each bar represents the mean + SD. *P < 0.05 versus each single agent; the Wilcoxon rank sum test using the Holm–Bonferroni method. (**g**) Immunoblots of tumor lysates treated as described in supplementary Fig. 7f for 6 hours. (**h**) Body weight change of mice relative to the start of the treatment. (**i**) Cells transfected with two siRNAs against FGFR1, FGF2, non-targeting control, or the combination of both siRNAs were cultured with osimertinib, neratinib, or dabrafenib for 6 days. Immunoblots of cell lysates 2 days after transfection. The viability in 1000 nM of TKIs with these siRNAs were shown. Each bar represents the mean + SD. *P < 0.05 versus siControl; Student's t-test using the Holm–Bonferroni method.

a

	HCC827	II-18	HCC1569	SK-BR-3	COLO 679
	Osimertinib (nM)	Osimertinib (nM)	Neratinib (nM)	Neratinib (nM)	Dabrafenib (nM)
Parental cells	5.7	42.3	527.5	0.05	2.2
DTP cells	>1000	>1000	>1000	720	627.6
Regrown cells	9.9	147	698.5	0.04	3.6

		FGFR-TKI (nM)
HCC827	Parental cells	>1000
	DTP cells	6.6
	Regrown cells	>1000
II-18	Parental cells	>1000
	DTP cells	>1000
	Regrown cells	>1000
HCC1569	Parental cells	>1000
	DTP cells	53.2
	Regrown cells	>1000
SK-BR-3	Parental cells	>1000
	DTP cells	>1000
	Regrown cells	>1000
COLO 679	Parental cells	>1000
	DTP cells	>1000
	Regrown cells	>1000

b

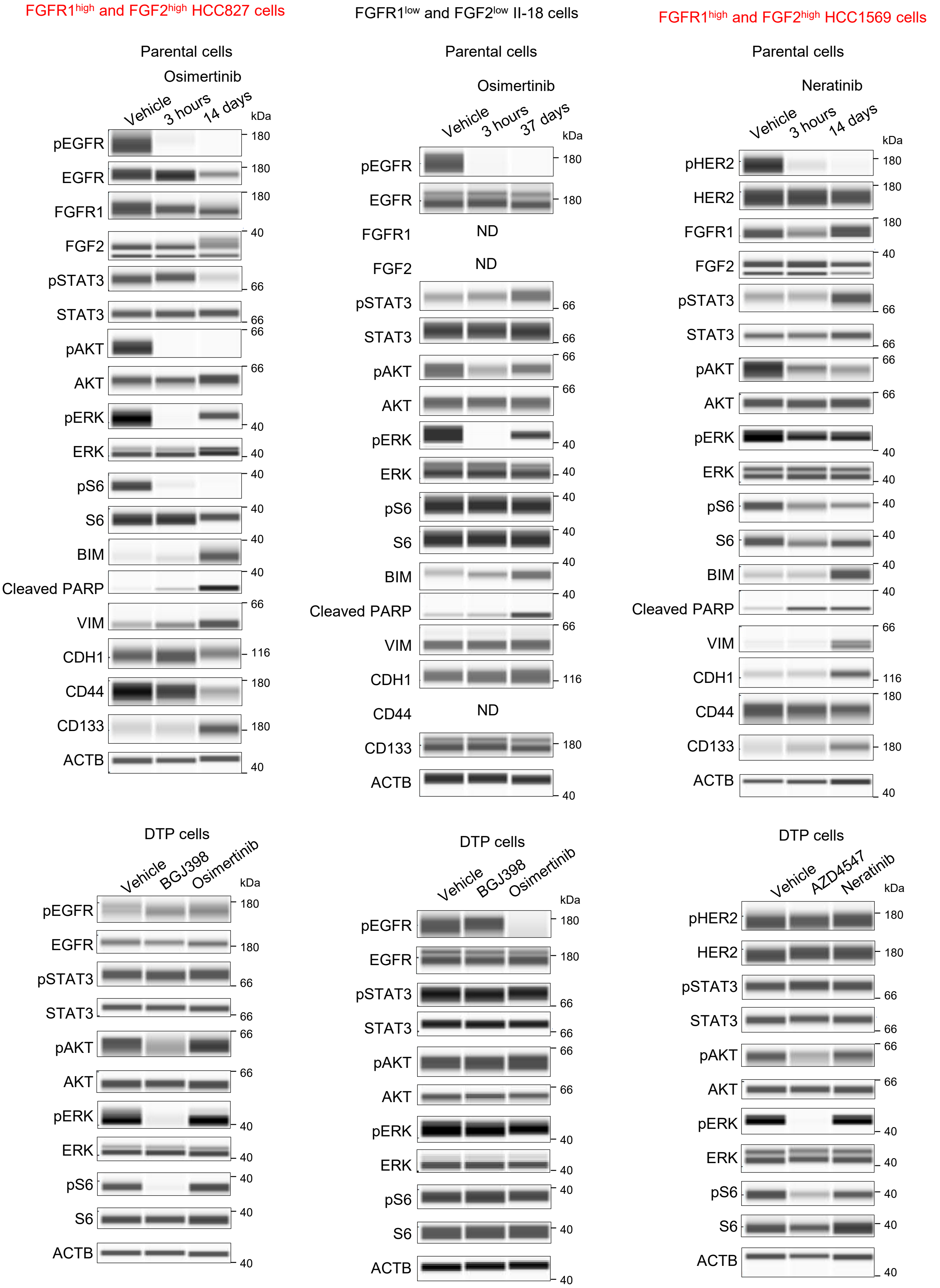

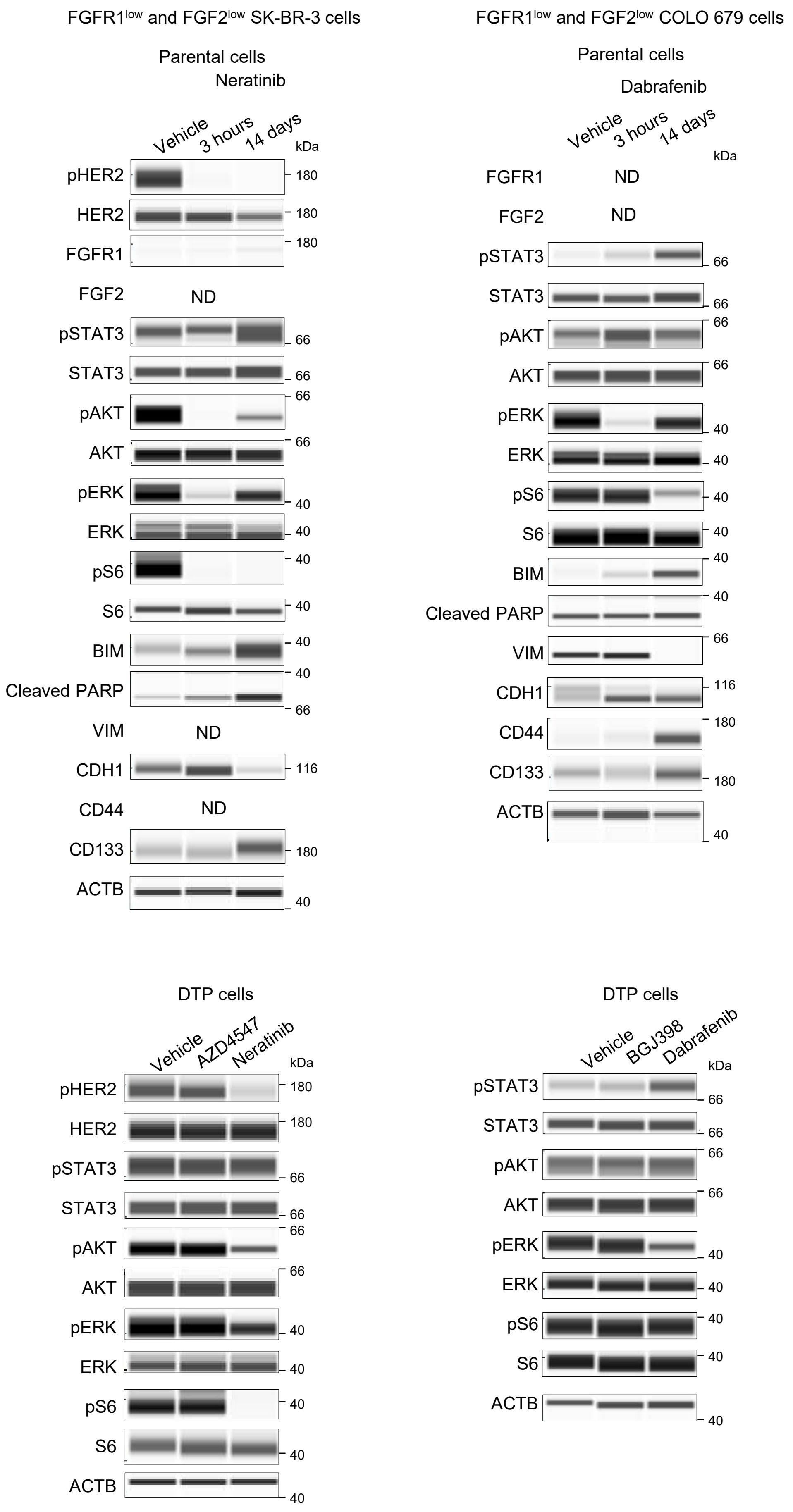

Supplementary Figure 8. Effect of FGFR inhibitors on parental, DTP, and regrown cells with EGFR, HER2 or BRAF mutations.

(**a**) IC_{50} values of parental, DTP, and regrown cells of HCC827, II-18, and COLO 679 cells with BGJ398. In HCC1569 cells, IC_{50} and IC_{60} values respect to neratinib and AZD4547 were shown, respectively. In SK-BR-3 cells, IC_{50} values respect to neratinib and AZD4547 were shown. ND; Not determined. (**b**) Immunoblots of cell lysates of parental cells treated as described in Fig. 6 for the indicated time. Immunoblots of cell lysates of DTP cells treated with 100 nM osimertinib (HCC827, II-18), 100 nM neratinib (HCC1569, SK-BR-3), 10 nM dabrafenib (COLO 679), 300 nM BGJ398 or AZD4547 for 3 hours. ND; Not detected any signals.

a

Cells		Alectinib (nM)	Osimertinib (nM)	BGJ398 (nM)	Capmatinib (nM)	Alectinib+BGJ398 (nM)	Osimertinib +BGJ398 (nM)	Osimertinib +capmatinib (nM)	Osimertinib +BGJ398+ capmatinib (nM)
NCI-H2228	Parental cells	219.6	ND	>1000	ND	37.8	ND	ND	ND
	Resistant cells	>1000	ND	>1000	ND	38.8	ND	ND	ND
HCC827	Parental cells	ND	3.6	>1000	>1000	ND	2.4	3.0	2.3
	Resistant cells	ND	>1000	>1000	>1000	ND	515.0	11.0	2.7

b

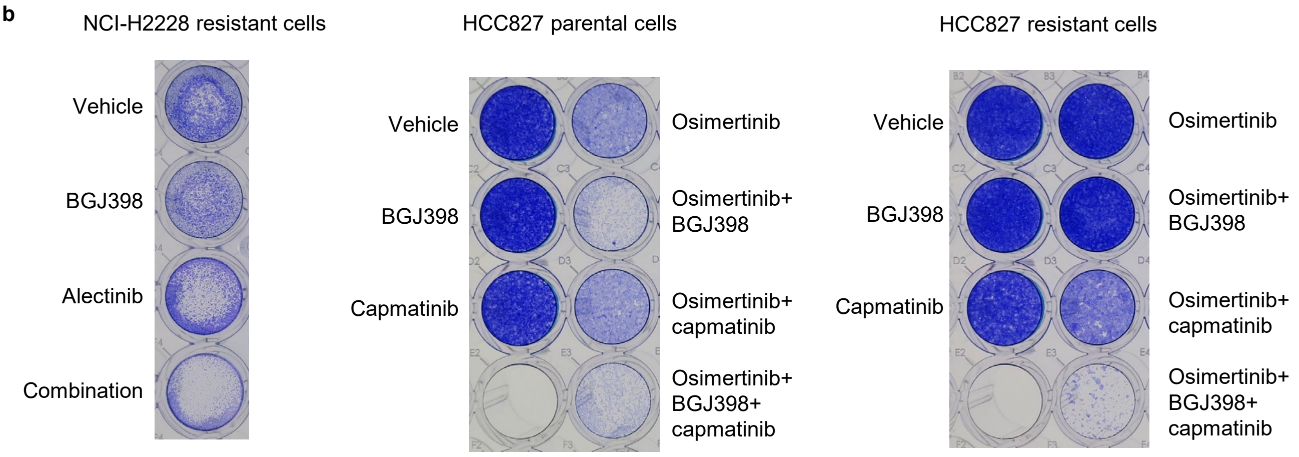

c

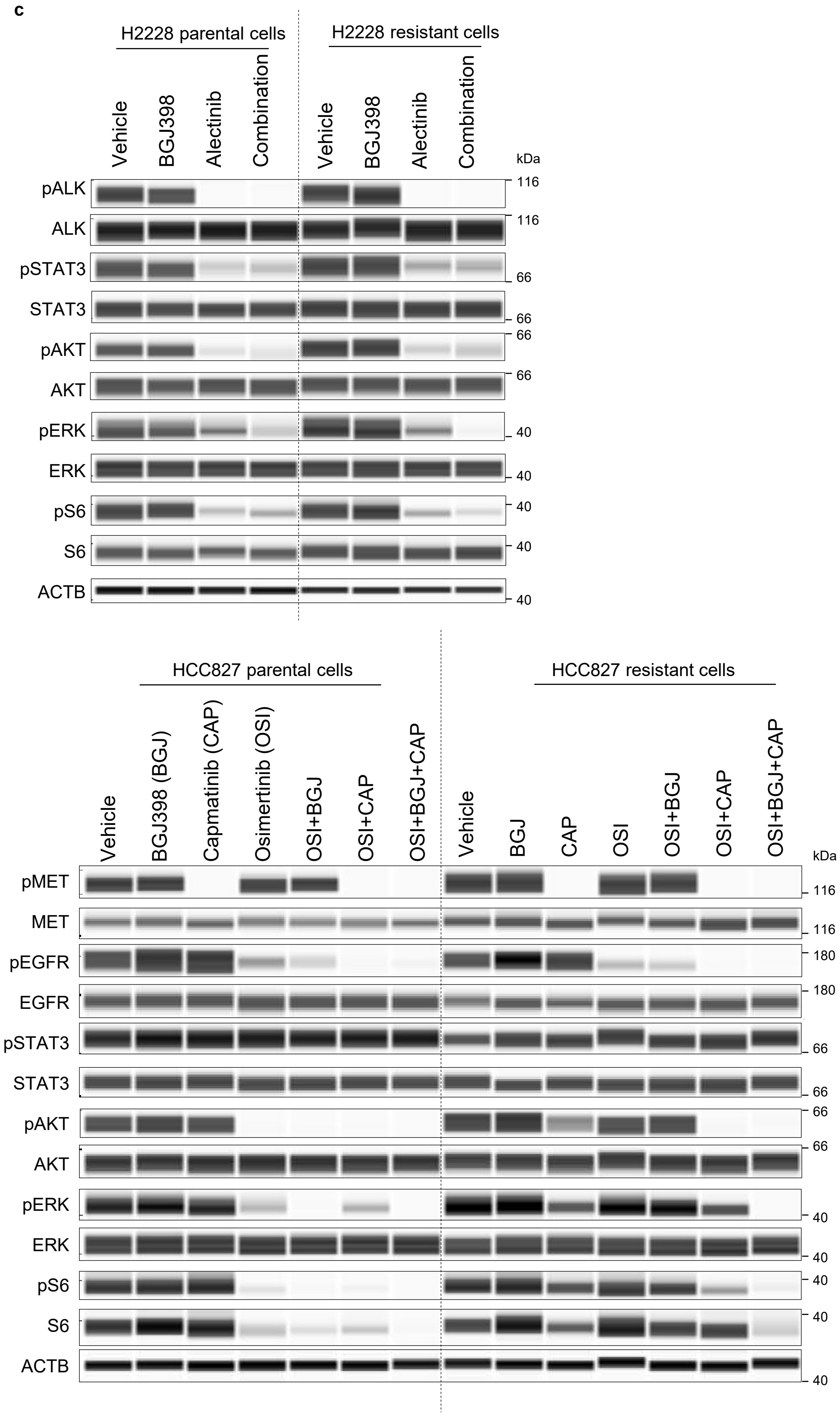

Supplementary Figure 9. Combination with FGFR inhibitor is effective on alectinib- and osimertinib-resistant cells.

(**a**) IC_{50} values of parental and resistant cells. (**b**) Crystal violet cell growth assays treated with 1000 nM of alectinib, osimertinib, 300 nM BGJ398, 10 nM capmatinib, and the combination of two or three drugs for 8 days. (**c**) Immunoblots of cell lysates from parental and resistant cells treated with1000 nM alectinib, 100 nM osimertinib, 300 nM BGJ398, and 10nM capmatinib for 3 hours.

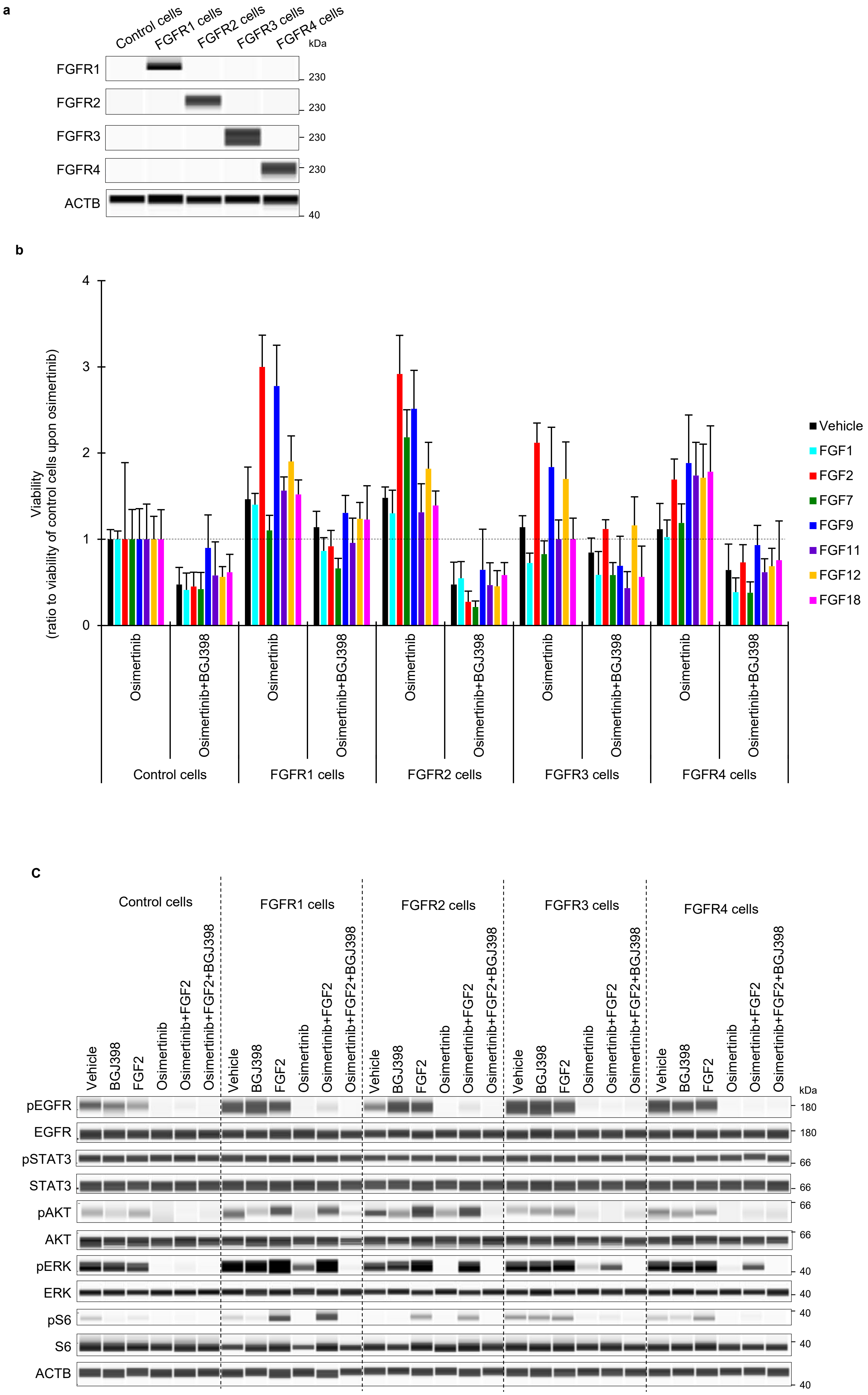
a
Control cells
FGFR1 cells
FGFR2 cells
FGFR3 cells
FGFR4 cells
kDa
FGFR1
FGFR2
FGFR3
FGFR4
ACTB
230
230
230
230
40
b
Viability
(ratio to viability of control cells upon osimertinib)
0
1
2
3
4
Vehicle
FGF1
FGF2
FGF7
FGF9
FGF11
FGF12
FGF18
Osimertinib
Osimertinib+BGJ398
Control cells
FGFR1 cells
FGFR2 cells
FGFR3 cells
FGFR4 cells
c
Control cells
FGFR1 cells
FGFR2 cells
FGFR3 cells
FGFR4 cells
Vehicle
BGJ398
FGF2
Osimertinib
Osimertinib+FGF2
Osimertinib+FGF2+BGJ398
kDa
pEGFR
EGFR
pSTAT3
STAT3
pAKT
AKT
pERK
ERK
pS6
S6
ACTB
180
180
66
66
66
66
40
40
40
40
40

d

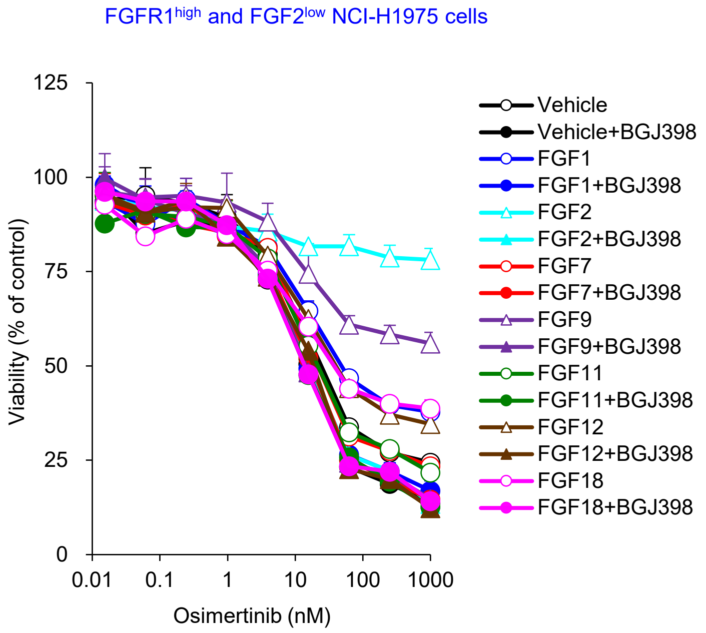

Supplementary Figure 10. FGF2 and FGF9 recovers osimertinib-induced inhibitory effect in FGFR1high or FGFR2high expressing cells.

(**a**) Immunoblots of cell lysates of II-18 cells transfected with lentiviral overexpression vector of non-targeting control, FGFR1, FGFR2, FGFR3 or FGFR4 protein. (**b**) Cells were cultured with 100 nM osimertinib in the presence of 50 ng/mL FGF ligands including FGF1, FGF2, FGF7, FGF9, FGF11, FGF12 or FGF18 with or without 300nM BGJ398 using 0.01% FBS RPMI1640 medium for 6 days. The ratio of control cells to osimertinib-exposed viability in the presence of each FGF was calculated. (**c**) Immunoblots of cell lysates of control, FGFR1, FGFR2, FGFR3 or FGFR4 overexpressing II-18 cells treated with 100 nM osimertinib in the presence of 50 ng/mL FGFs with or without 300 nM BGJ398 for 3 hours. (**d**) NCI-H1975 cells were cultured with osimertinib in the presence of 50 ng/mL FGF with or without 300nM BGJ398 for 8 days. Their viability with the treatments relative to their viability with vehicle was measured.

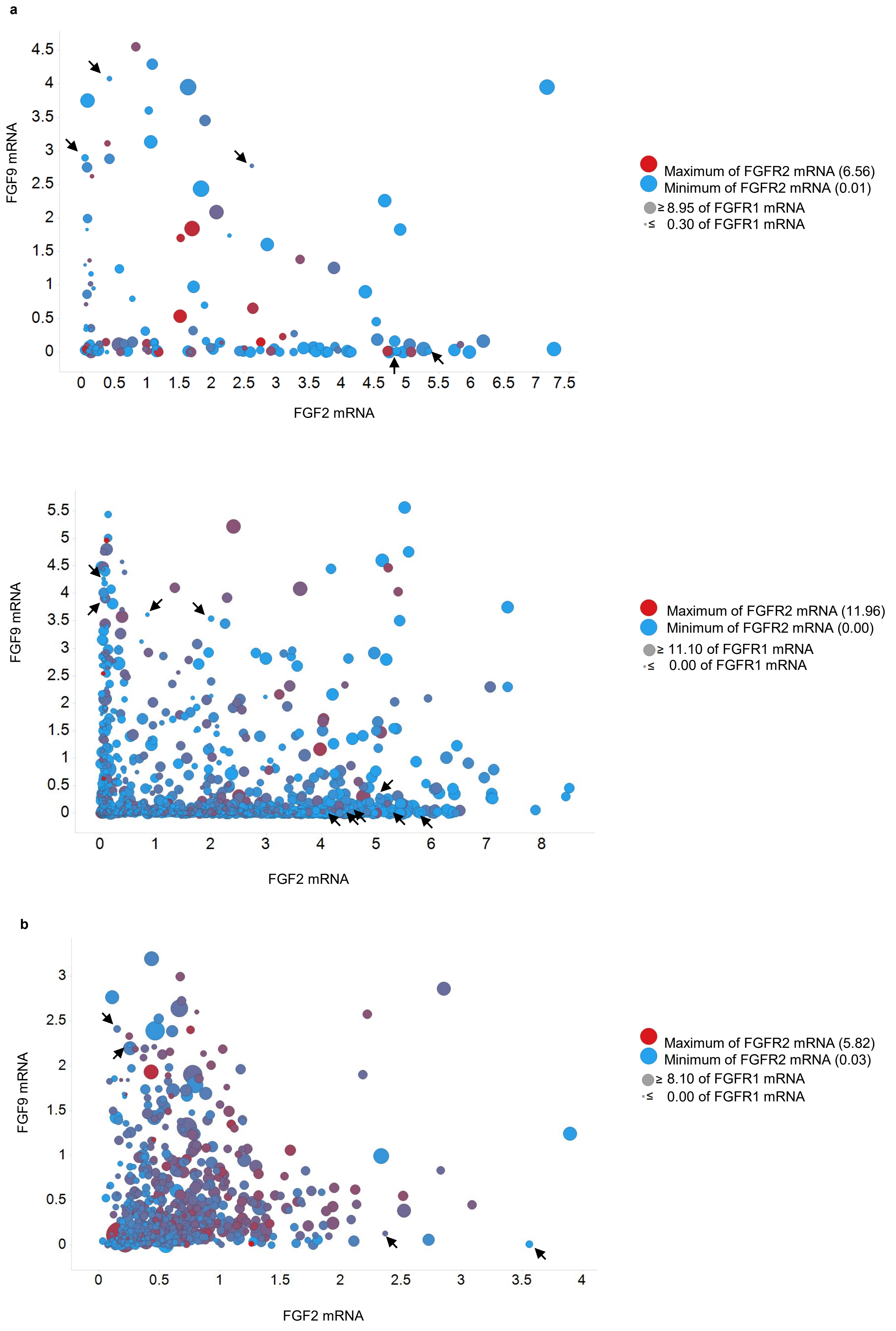

Supplementary Figure 11. Expression of FGF2 or FGF9 with FGFR1 or FGFR2 mRNA in cancer.

(A) The mRNA levels (log2(TPM+1)) of FGF2 or FGF9 with FGFR1 or FGFR2 in 135 NSCLC cell lines and 1182 cancer cell lines other than lung in DepMap database. (B) The mRNA levels (log2(FPKM+1)) of FGF2 or FGF9 with FGFR1 or FGFR2 in 607 primary tumor tissue samples of bronchus and lung adenomas and adenocarcinoma patients in the GDC Data Portal database (https://portal.gdc.cancer.gov/projects). These figures were shown by Spotfire software ver. 7.11.1.0.13. Arrows indicated FGF2 or FGF9 relatively high-expressing cells or patients with relatively low expression of FGFR1 or FGFR2.

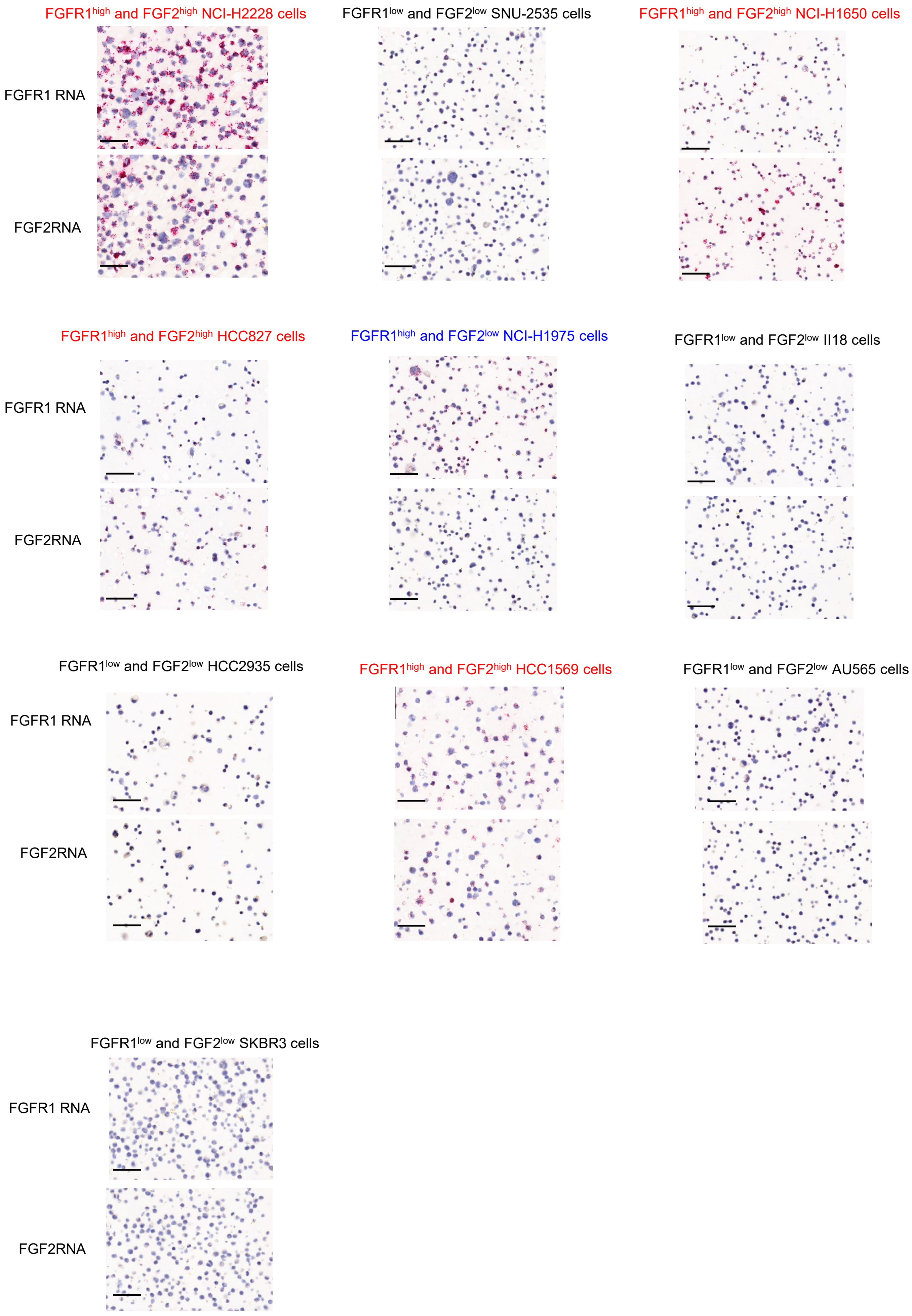

Supplementary Figure 12. Detection of FGFR1 and FGF2 mRNA expression in cancer cells via RNAscope.

Representative examples of FGFR1 and FGF2 staining of FFPE cell blocks of NCI-H1650, HCC827, NCI-H1975, II18, HCC2935, AU565, and SKBR3 cells using RNAscope assay via Advanced Cell Diagnostics. Scale bars indicate 50 µm.

Cell line	Tissue	Oncogenic driver mutation	Supplier	Culture medium
NCI-H2228	NSCLC	EML4-ALK[1]	ATCC	10%FBS-RPMI1640
SNU-2535	NSCLC	EML4-ALK, G1269A[2]	KCLB	10%FBS-RPMI1640
NCI-H3122	NSCLC	EML4-ALK	CLS	10%FBS-RPMI1640
NCI-H1650	NSCLC	EGFR E746_A750 DL[1]	ATCC	10%FBS-RPMI1640
HCC827	NSCLC	EGFR E746_A750 DL[1]	ATCC	10%FBS-RPMI1640
NCI-H1975	NSCLC	EGFR L858R, T790M[1]	ATCC	10%FBS-RPMI1640
II-18	NSCLC	EGFR L858R[1]	RIKEN	10%FBS-RPMI1640
HCC2935	NSCLC	EGFR E746_T751 DL, S752I[6]	ATCC	10%FBS-RPMI1640
HCC1569	BC	HER2 amplification[1]	ATCC	10%FBS-RPMI1640
AU565	BC	HER2 amplification[1]	ATCC	10%FBS-RPMI1640
SK-BR-3	BC	HER2 amplification[3]	ATCC	10%FBS-McCoy's 5a
RPMI-7951	Melanoma	BRAF V600E[1]	ATCC	10%FBS-EMEM
IGR-39	Melanoma	BRAF V600E[1]	DSMZ	15%FBS-DMEM
SK-MEL-3	Melanoma	BRAF V600E[1]	ATCC	15%FBS-McCoy's 5a
A2058	Melanoma	BRAF V600E[4]	JCRB	10%FBS-DMEM
A101D	Melanoma	BRAF V600E[5]	ATCC	10%FBS-DMEM
COLO 679	Melanoma	BRAF V600E[1]	ECACC	10%FBS-RPMI1640

Supplementary Table 1. List of human cancer cell lines.

All cancer cell lines were shown in this study. II-18 cell line was provided by the RIKEN BRC through the National Bio-Resource Project of the MEXT, Japan. NSCLC: non-small cell lung cancer, BC: breast cancer, DL Deletion. ATCC: American Type Culture Collection, KCLB: Korean Cell Line Bank, RIKEN: RIKEN BioResource Research Center, DSMZ: DSMZ-German Collection of Microorganisms and Cell Cultures. JCRB: Japanese Collection of Research Bioresources. ECACC: European Collection of Authenticated Cell Cultures. Mutations were referred from COSMIC cell database (1); Yoshimura et al. (2); Liu et al. (3); Xiao et al. (4); Yuan et al. (5); ATCC's data sheet.

References

1. https://cancer.sanger.ac.uk/cosmic, date of last visit was February27th, 2022.
2. Y. Yoshimura *et al.*, Antitumor activity of alectinib, a selective ALK inhibitor, in an ALK-positive NSCLC cell line harboring G1269A mutation: Efficacy of alectinib against ALK G1269A mutated cells. *Cancer Chemother Pharmacol* **77**, 623-628 (2016).
3. Q. Liu *et al.*, A novel HER2 gene body enhancer contributes to HER2 expression. *Oncogene* **37**, 687-694 (2018).
4. J. Xiao, M. E. Egger, K. M. McMasters, H. Hao, Differential expression of ABCB5 in BRAF inhibitor-resistant melanoma cell lines. *BMC Cancer* **18**, 675-675 (2018).
5. J. Yuan *et al.*, The dimer-dependent catalytic activity of RAF family kinases is revealed through characterizing their oncogenic mutants. *Oncogene* **37**, 5719-5734 (2018).

Antibody	Supplier	Catalog number	Dilution
ALK	Cell Signaling Technology	3633	2000
Phospho-ALK	Cell Signaling Technology	3341	50
FGFR1	Cell Signaling Technology	9740	100
FGFR2	Cell Signaling Technology	23328	100
FGFR3	Cell Signaling Technology	4574	100
FGFR4	Cell Signaling Technology	2894	100
ERK	Cell Signaling Technology	9102	1000
Phospho-ERK	Cell Signaling Technology	4377	1000
AKT	Cell Signaling Technology	4691	500
Phospho-AKT	Cell Signaling Technology	4058	100
EGFR	Cell Signaling Technology	4267	100
Phospho-EGFR	Cell Signaling Technology	3777	100
MET	Cell Signaling Technology	4560	100
Phospho-MET	Cell Signaling Technology	3077	100
β-actin	Cell Signaling Technology	4970	500
HER2	Cell Signaling Technology	3250	25
Phospho-HER2	Abcam	ab47262	25
CD44	Cell Signaling Technology	37259	25
CD133	Cell Signaling Technology	86781	25
BIM	Cell Signaling Technology	2933	100
VIM	Cell Signaling Technology	5741	100
CDH1	Cell Signaling Technology	3195	100
S6	Cell Signaling Technology	2217	100
Phospho-S6	Cell Signaling Technology	5364	200
STAT3	Cell Signaling Technology	4904	1000
Phospho-STAT3	Cell Signaling Technology	9145	500
cleaved PARP	Abcam	ab32064	100
FGF2	Abcam	ab208687	1000

Supplementary Table 2. Details of the antibodies used in this study

siRNA	Catalog#	Target sequence
ON-TARGETplus Non-targeting Control siRNAs #1	D-001810-01	UGGUUUACAUGUCGACUAA
ON-TARGETplus Non-targeting Control siRNAs #2	D-001810-03	UGGUUUACAUGUUUUCUGA
ON-TARGETplus Human FGFR1 siRNA #1	J-003131-11	CCACAGAAUUGGAGGCUAC
ON-TARGETplus Human FGFR1 siRNA #2	J-003131-13	GAAAUUGCAUGCAGUGCCG
ON-TARGETplus Human FGF2 siRNA #1	J-006695-06	UCAAAGGAGUGUGUGCUAA
ON-TARGETplus Human FGF2 siRNA #2	J-006695-08	GAUGGAAGAUUACUGGCUU

Supplementary Table 3. Details of the siRNAs used in this study

Supplementary Data. Anti-cancer compound library screening in NCI-H2228 parental and DTP cells.

The DTP cells were generated from NCI-H2228 cells following treatment with 1000 nM alectinib for 13 days. The cells were cultured with the anti-cancer compound library for 6 days. This library was obtained using TargetMol. The names and targets of compounds, and the viability of parental and DTP cells are shown

Supplementary Figure 13 of uncropped immunoblots blots of Figure 1b

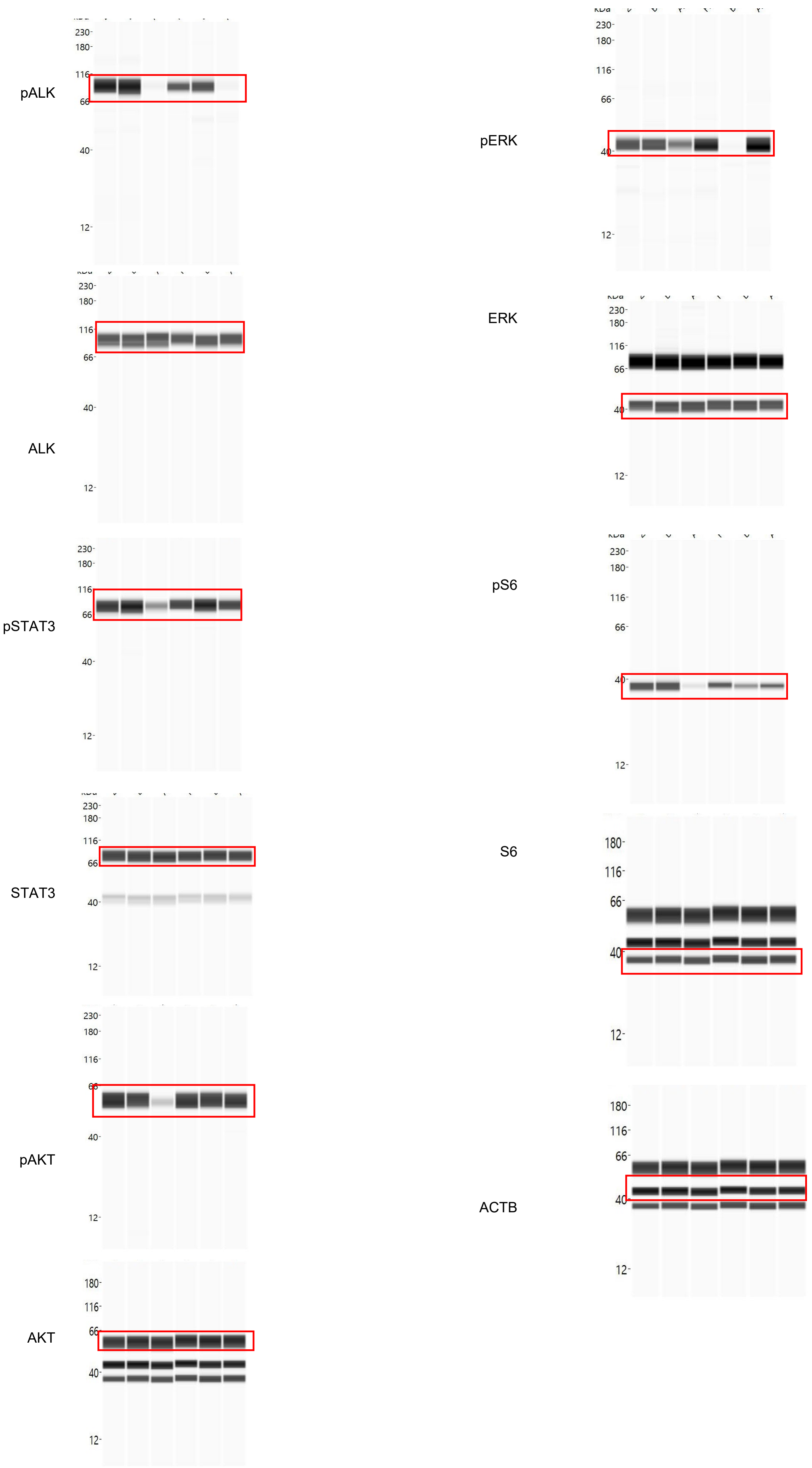

Supplementary Figure 13 of uncropped immunoblots blots of Figure 1d

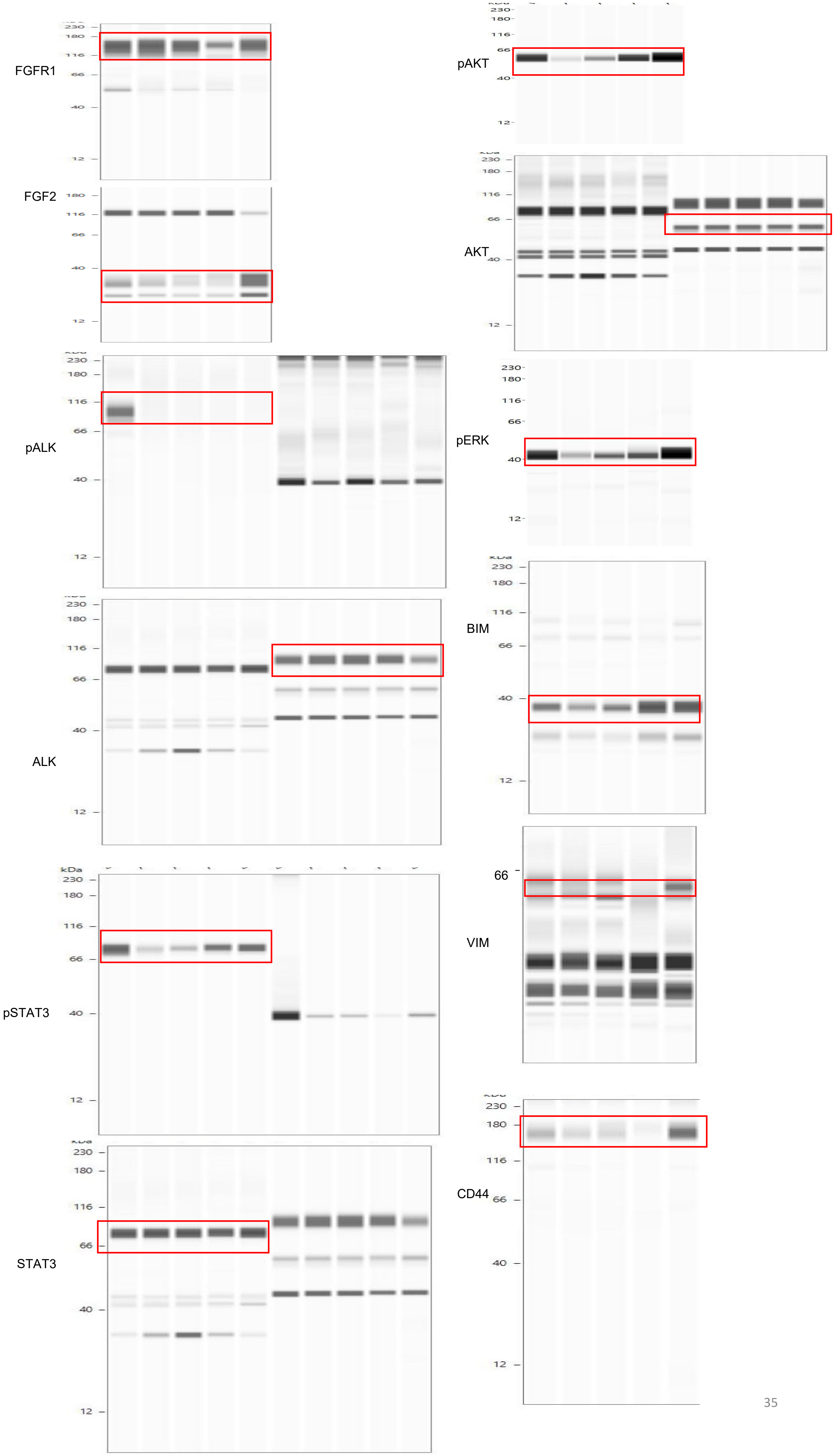

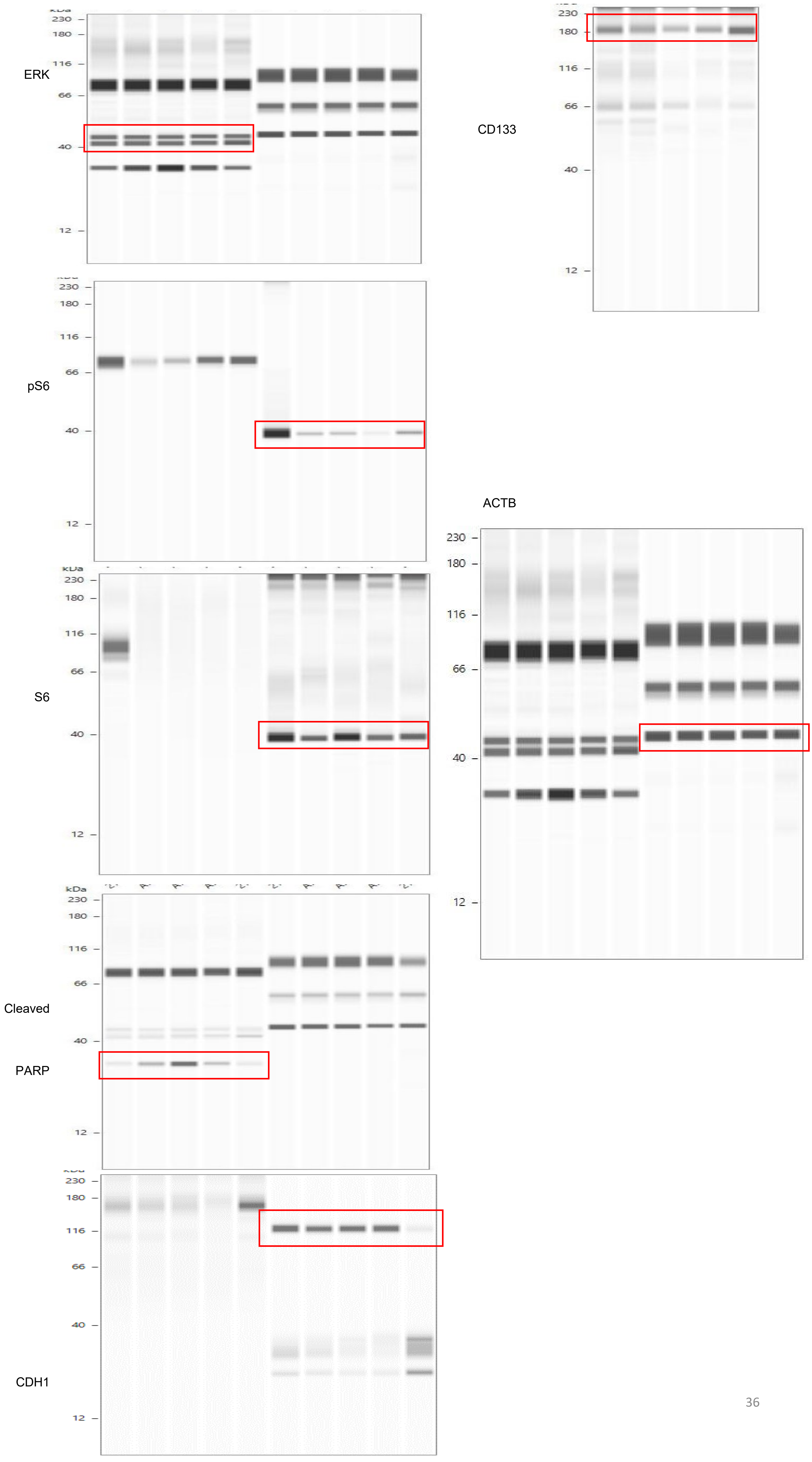
ERK
CD133
pS6
ACTB
S6
Cleaved
PARP
CDH1
kDa
230
180
116
66
40
12

Supplementary Figure 13 of uncropped immunoblots blots of Figure 2e

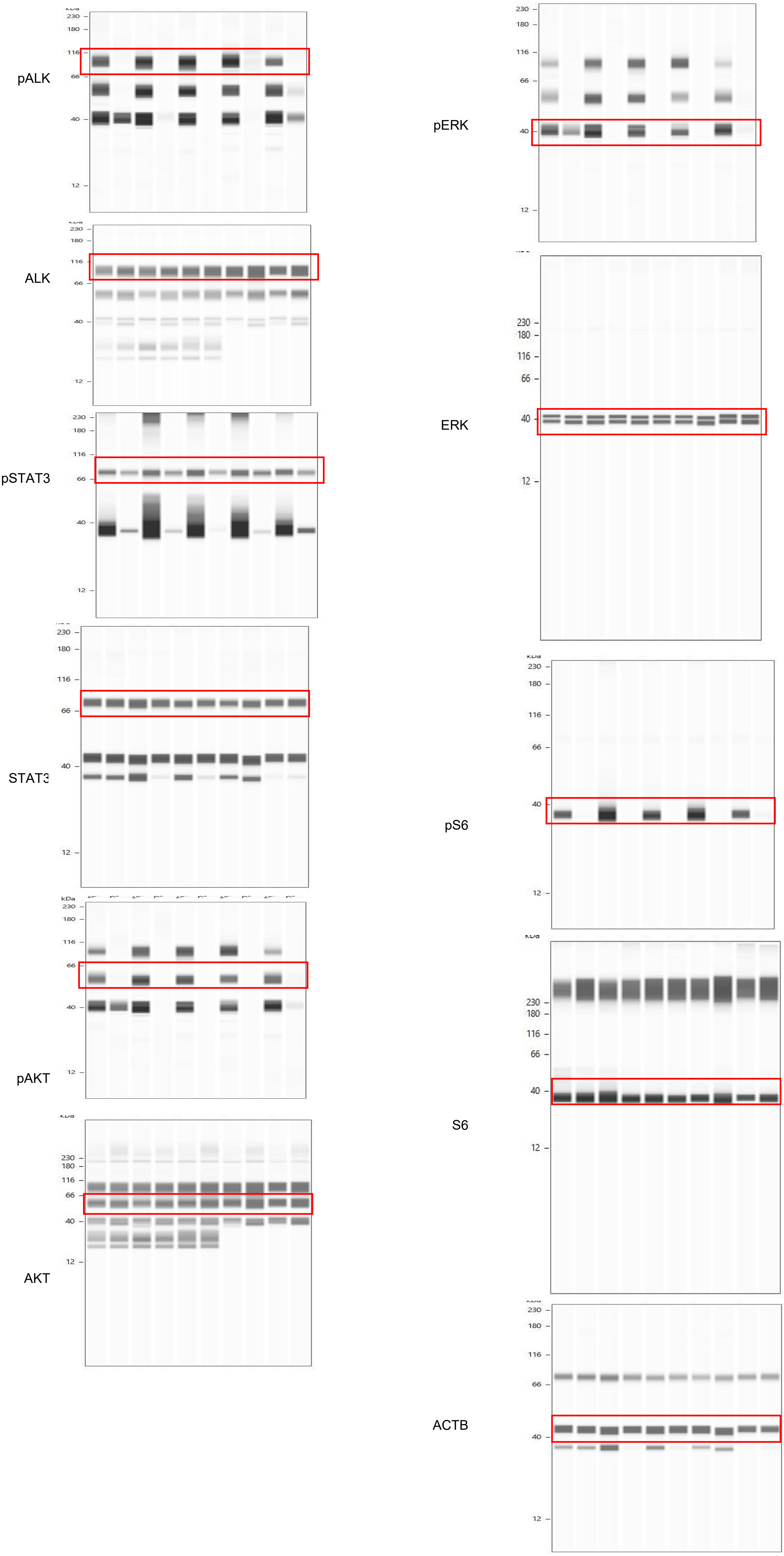

Supplementary Figure 13 of uncropped immunoblots blots of Figure 2c

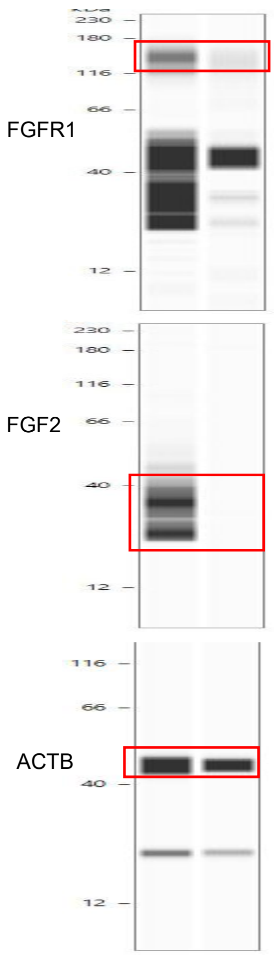

Supplementary Figure 13 of uncropped immunoblots blots of Figure 2f

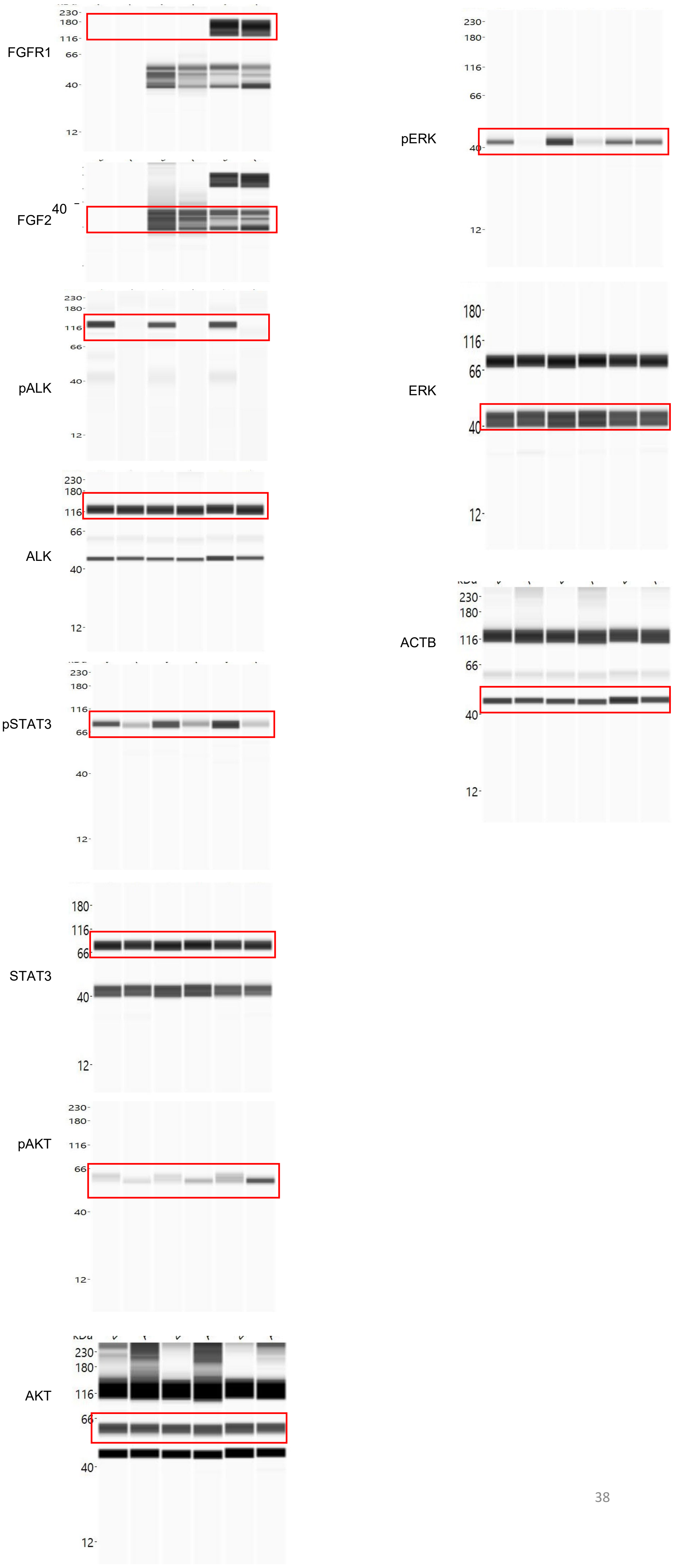

Supplementary Figure 13 of uncropped immunoblots blots of Figure 4c

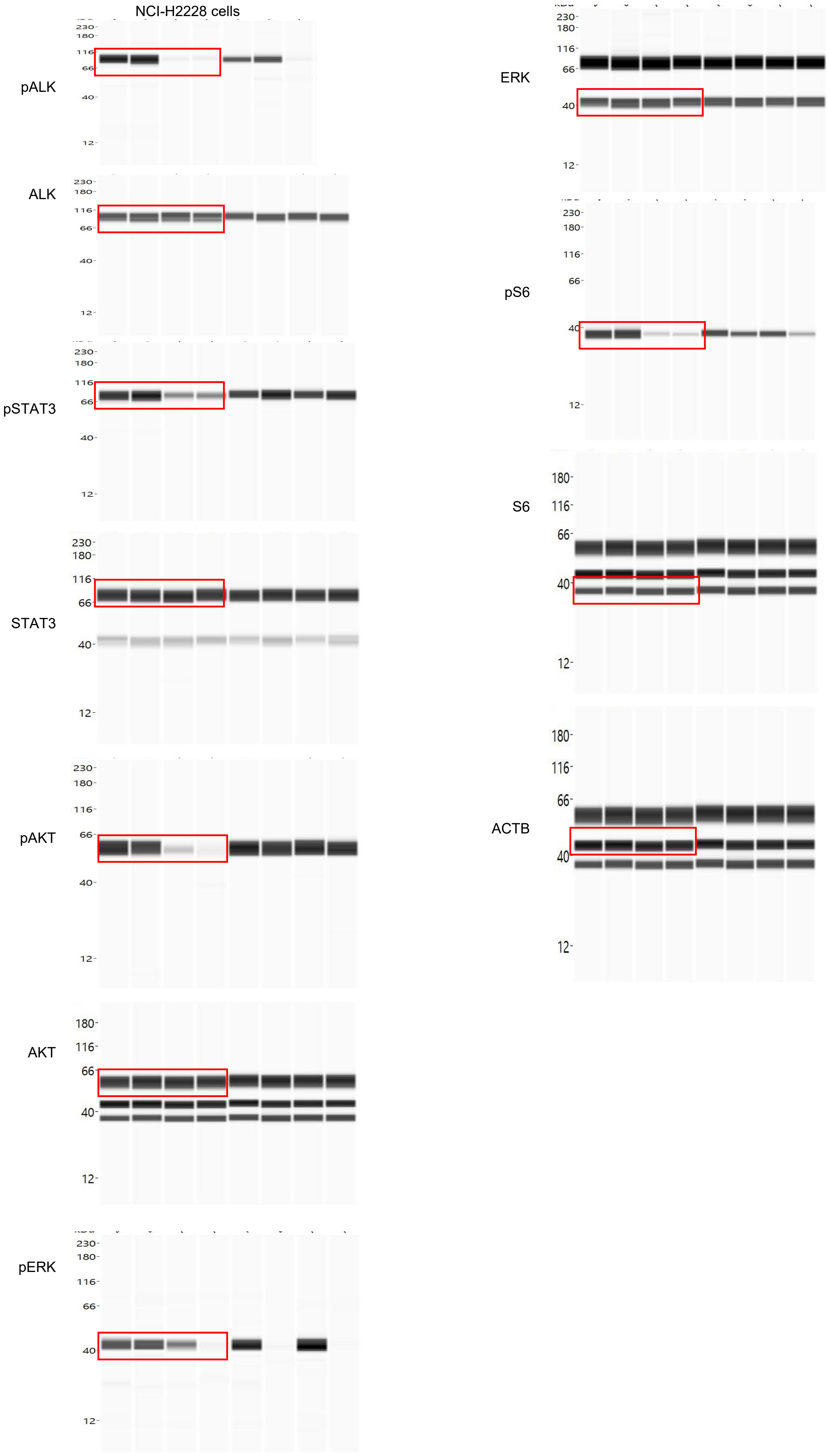

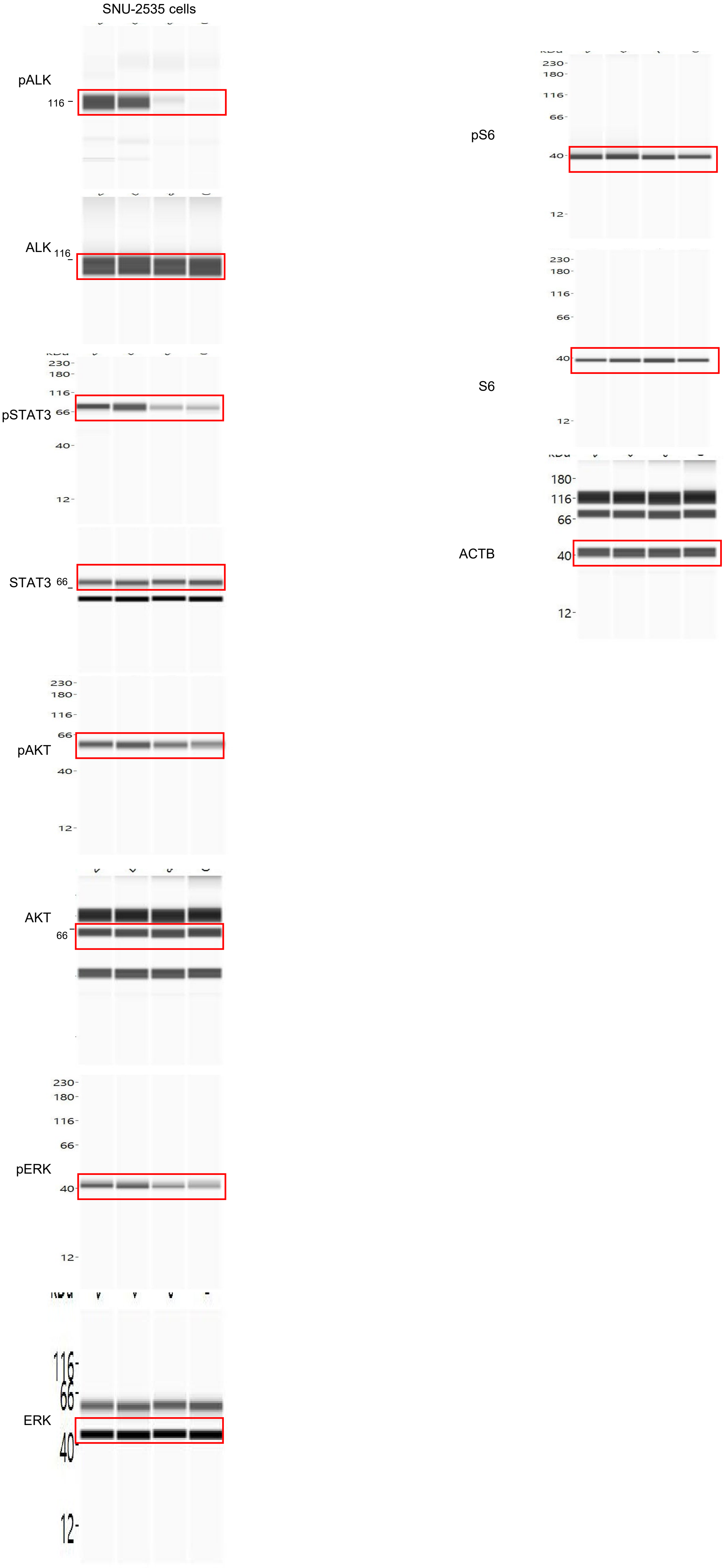
SNU-2535 cells
pALK
116
ALK
116
pSTAT3
230
180
116
66
40
12
STAT3
66
pAKT
230
180
116
66
40
12
AKT
66
pERK
230
180
116
66
40
12
ERK
116
66
40
12
pS6
230
180
116
66
40
12
S6
230
180
116
66
40
12
ACTB
180
116
66
40
12

Supplementary Figure 13 of uncropped immunoblots blots of Figure 4e

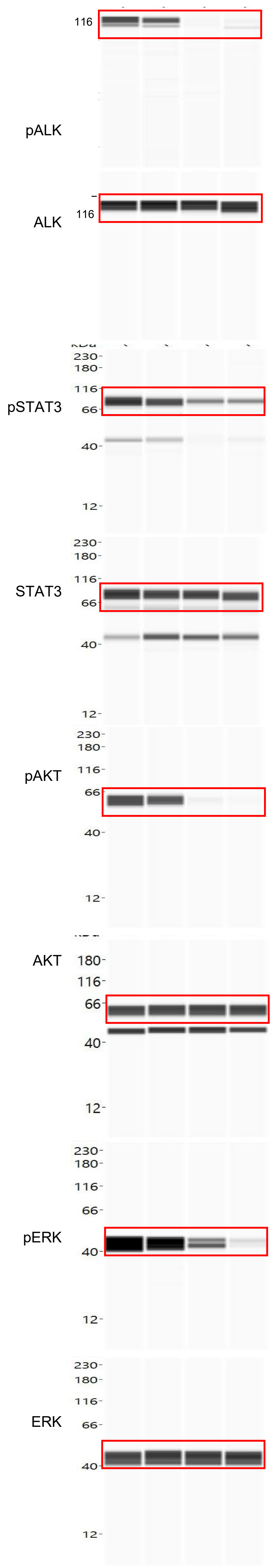

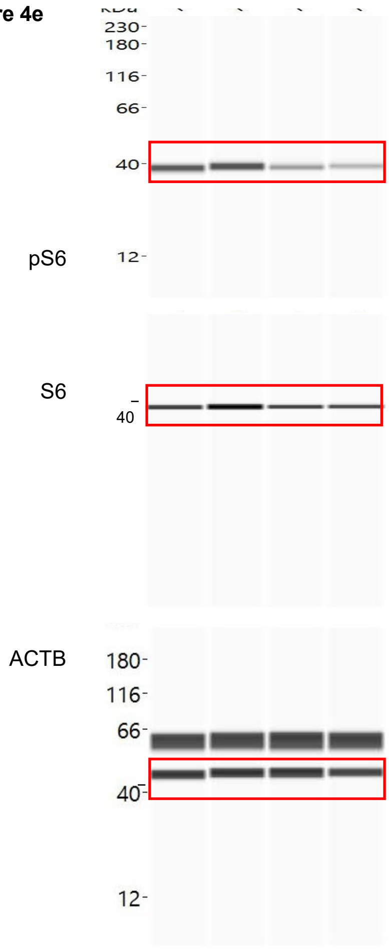

Supplementary Figure 13 of uncropped immunoblots blots of Figure 5a

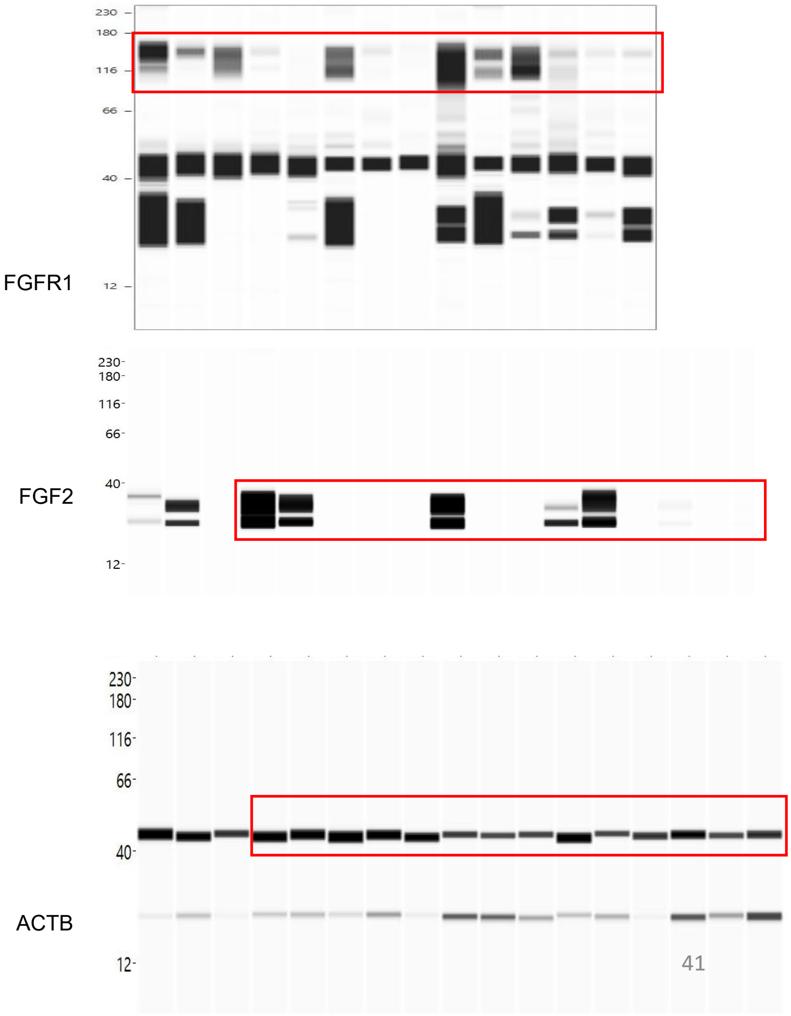

Supplementary Figure 13 of uncropped immunoblots blots of Figure 7c

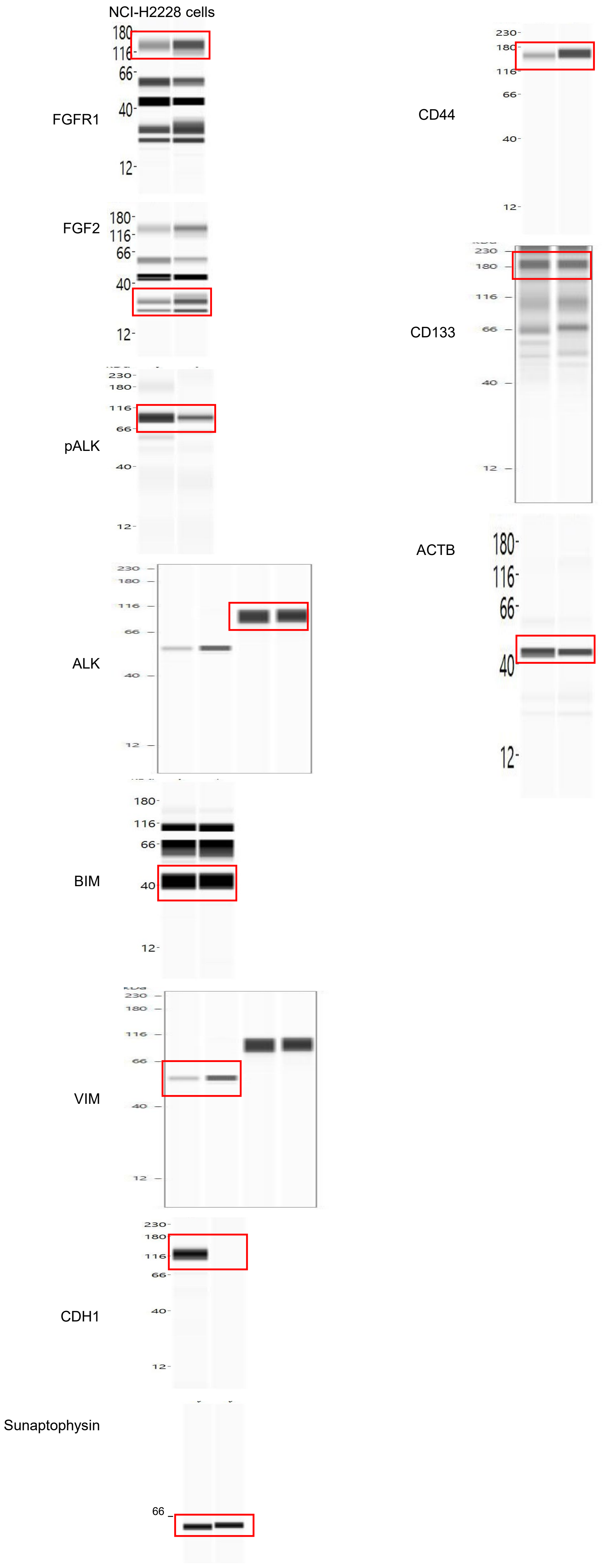

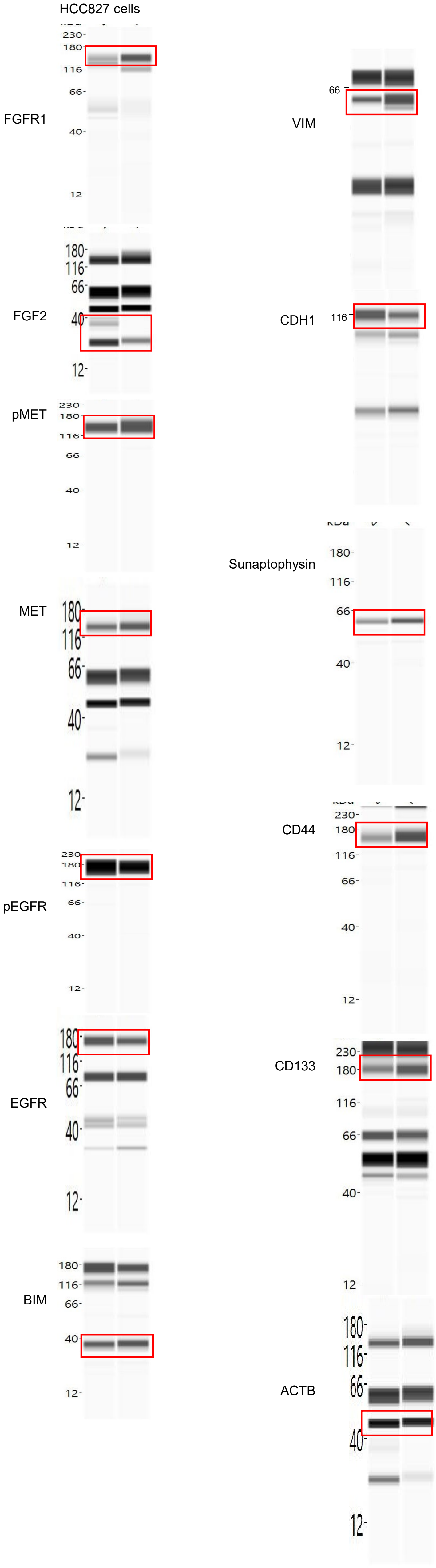
HCC827 cells
FGFR1
FGF2
pMET
MET
pEGFR
EGFR
BIM
VIM
CDH1
Sunaptophysin
CD44
CD133
ACTB
230
180
116
66
40
12

Supplementary Figure 13 of uncropped immunoblots blots of Supplementary Figure 4a

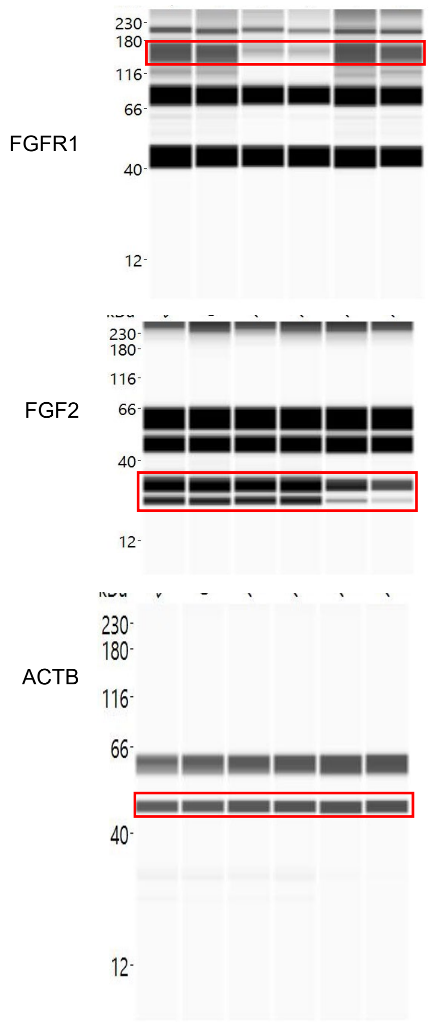

Supplementary Figure 13 of uncropped immunoblots blots of Supplementary Figure 4b

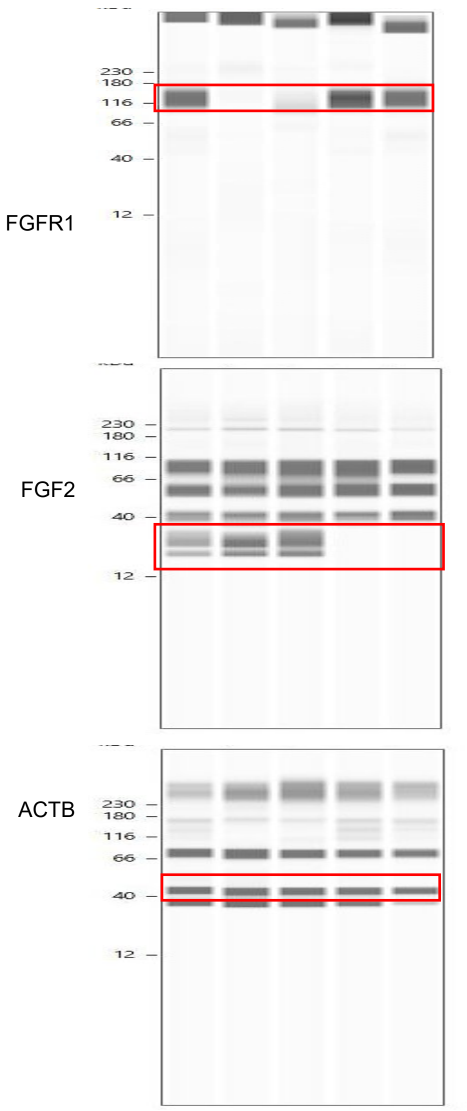

Supplementary Figure 13 of uncropped immunoblots blots of Supplementary Figure 4f

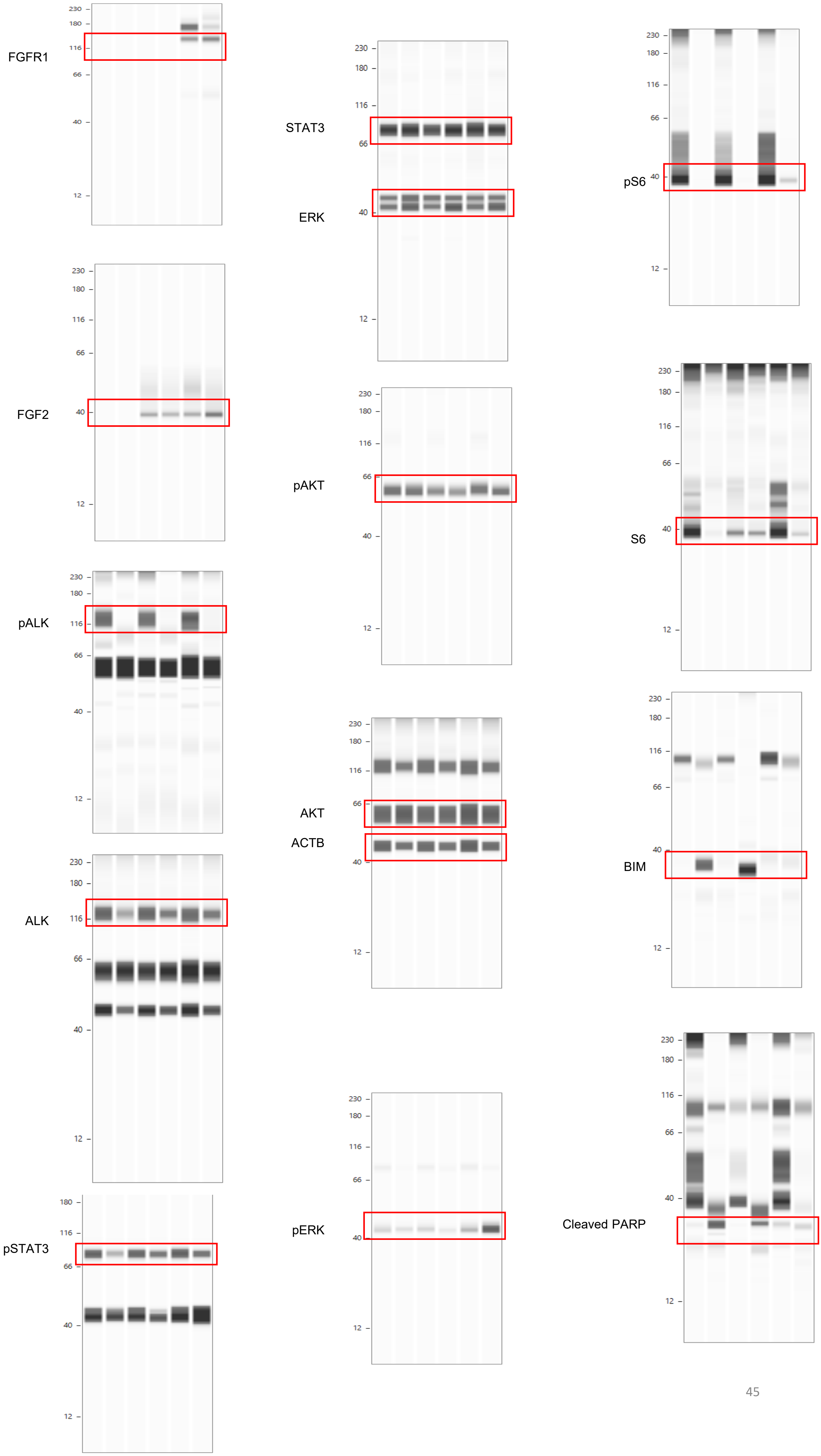

VIM

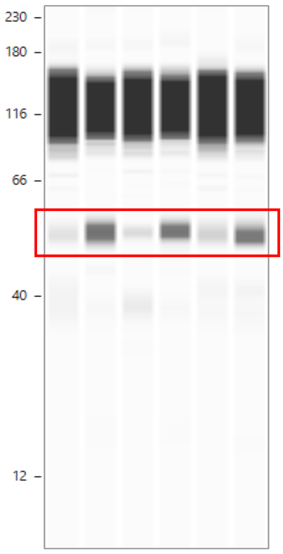

CDH1

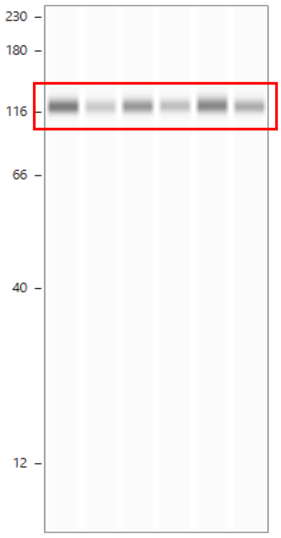

CD44

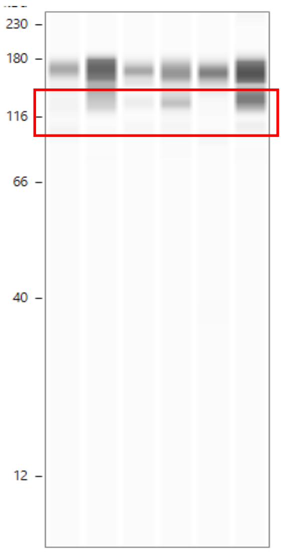

CD133

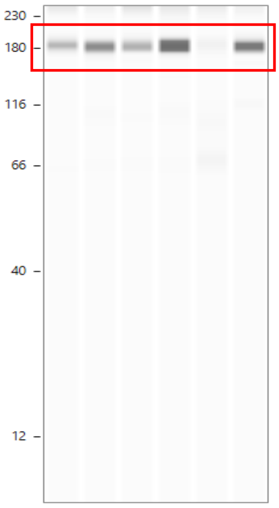

Supplementary Figure 13 of uncropped immunoblots blots of Supplementary Figure 4h

A. SNU2535 Lenti-Control cells

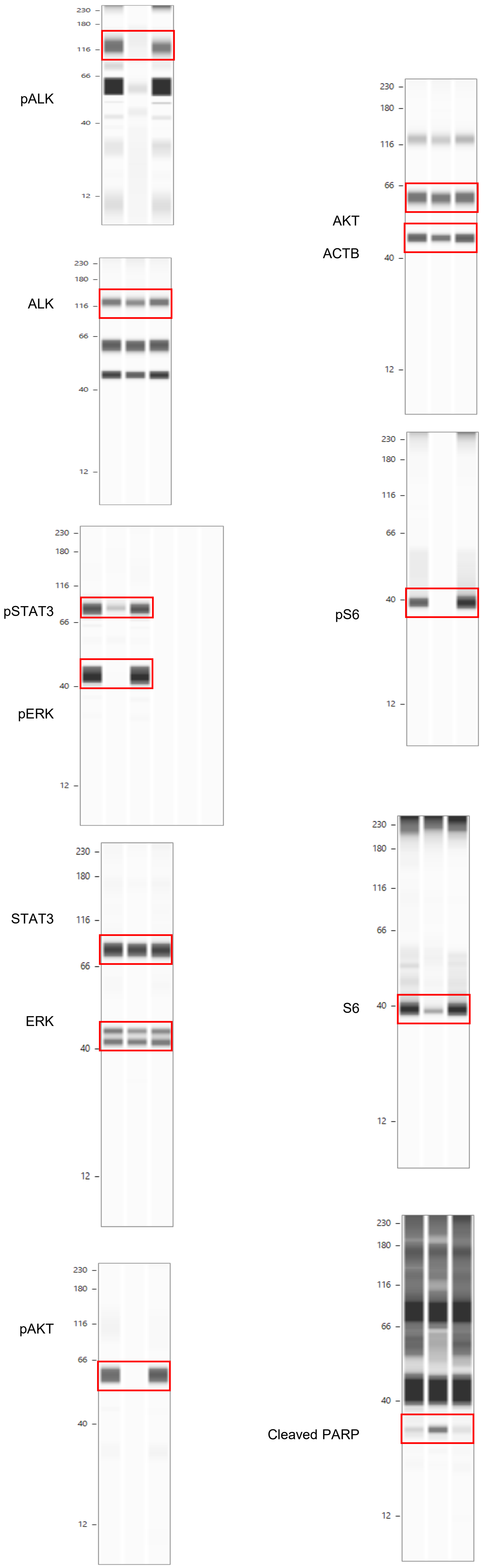

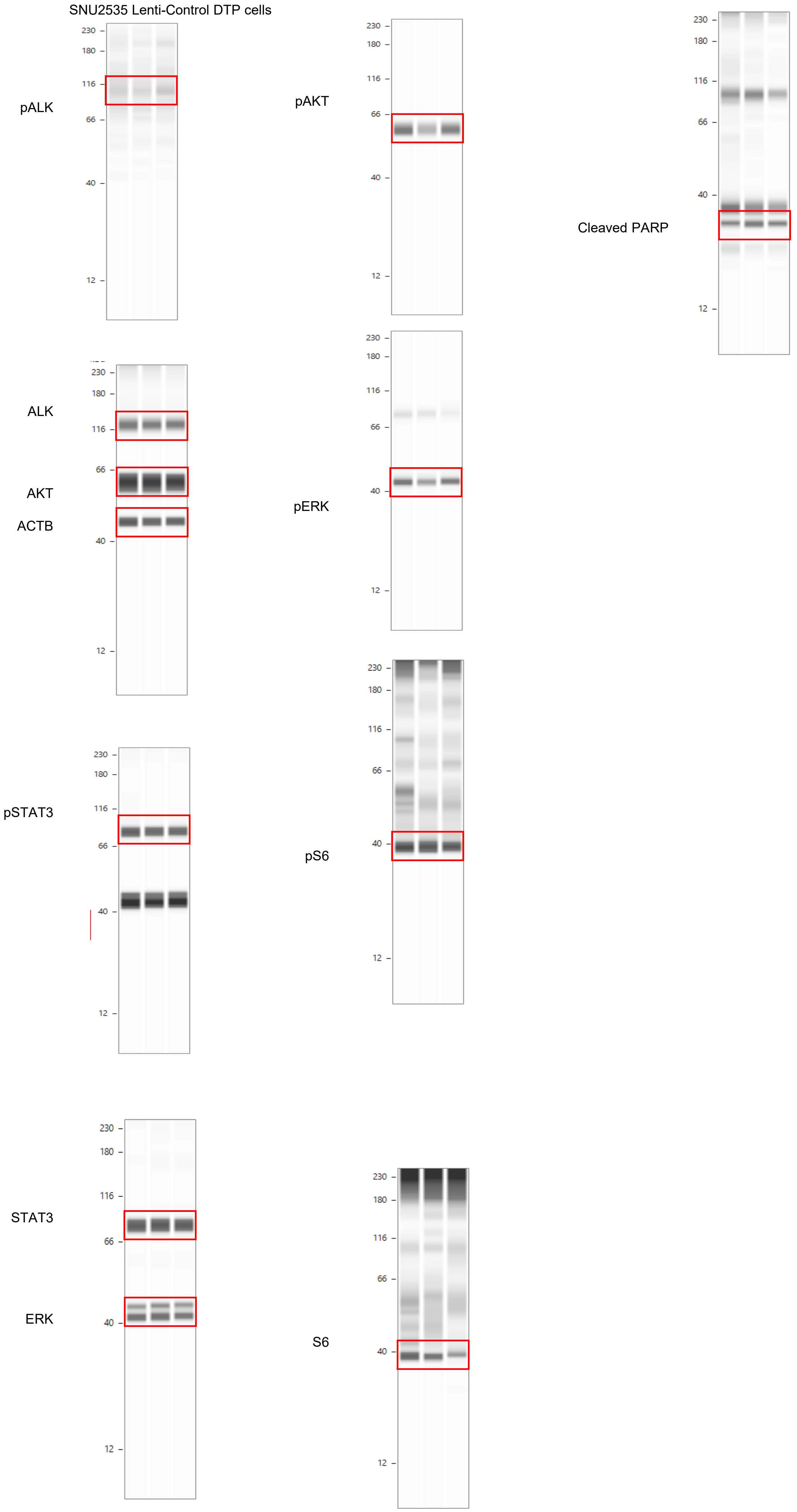
SNU2535 Lenti-Control DTP cells
pALK
ALK
AKT
ACTB
pSTAT3
STAT3
ERK
pAKT
pERK
pS6
S6
Cleaved PARP

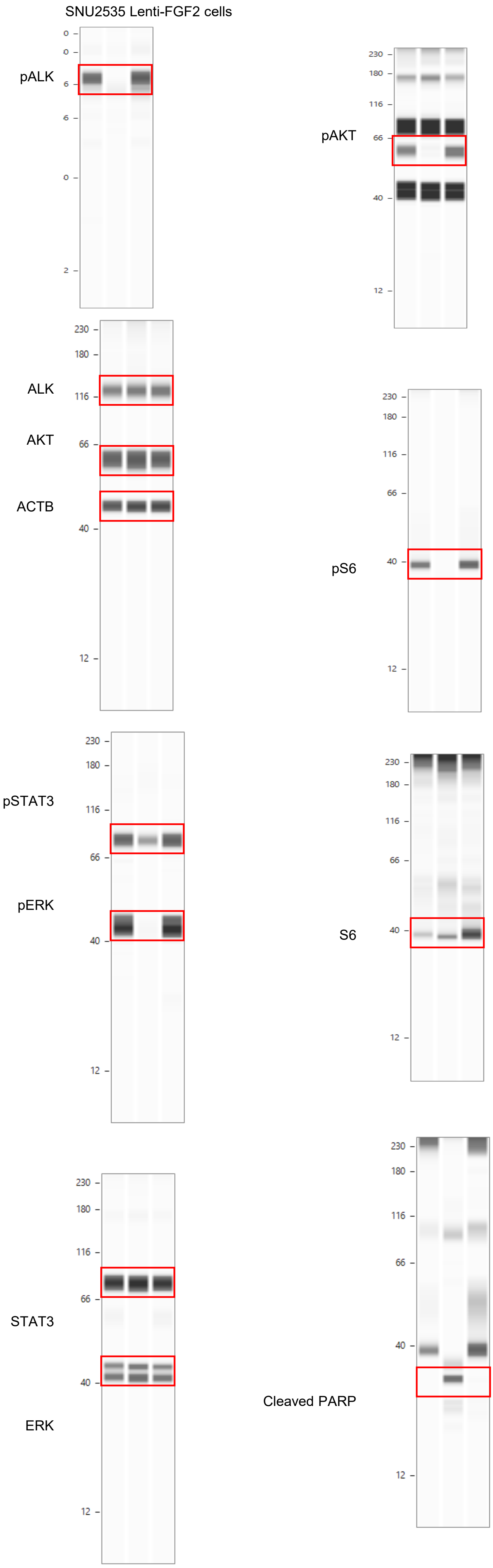
SNU2535 Lenti-FGF2 cells
pALK
pAKT
ALK
AKT
ACTB
pS6
pSTAT3
pERK
S6
STAT3
ERK
Cleaved PARP

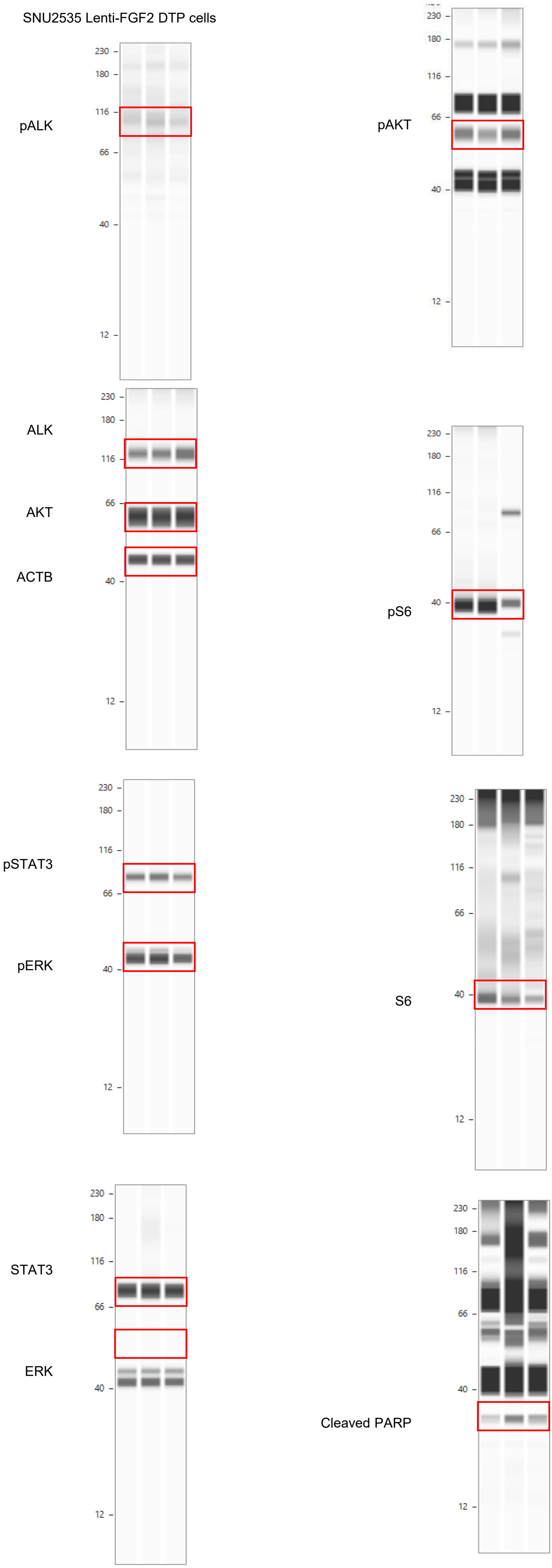
SNU2535 Lenti-FGF2 DTP cells
pALK
ALK
AKT
ACTB
pSTAT3
pERK
STAT3
ERK
pAKT
pS6
S6
Cleaved PARP
230
180
116
66
40
12

SNU2535 Lenti-FGFR1+FGF2 cells

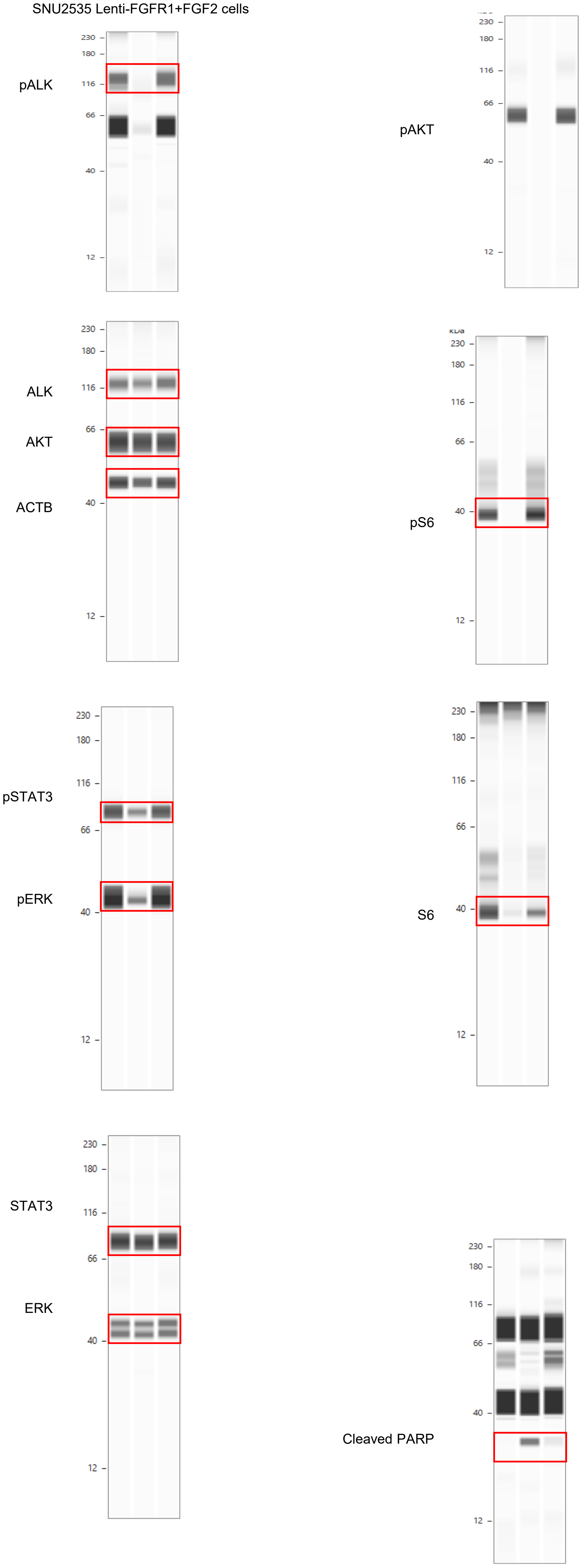

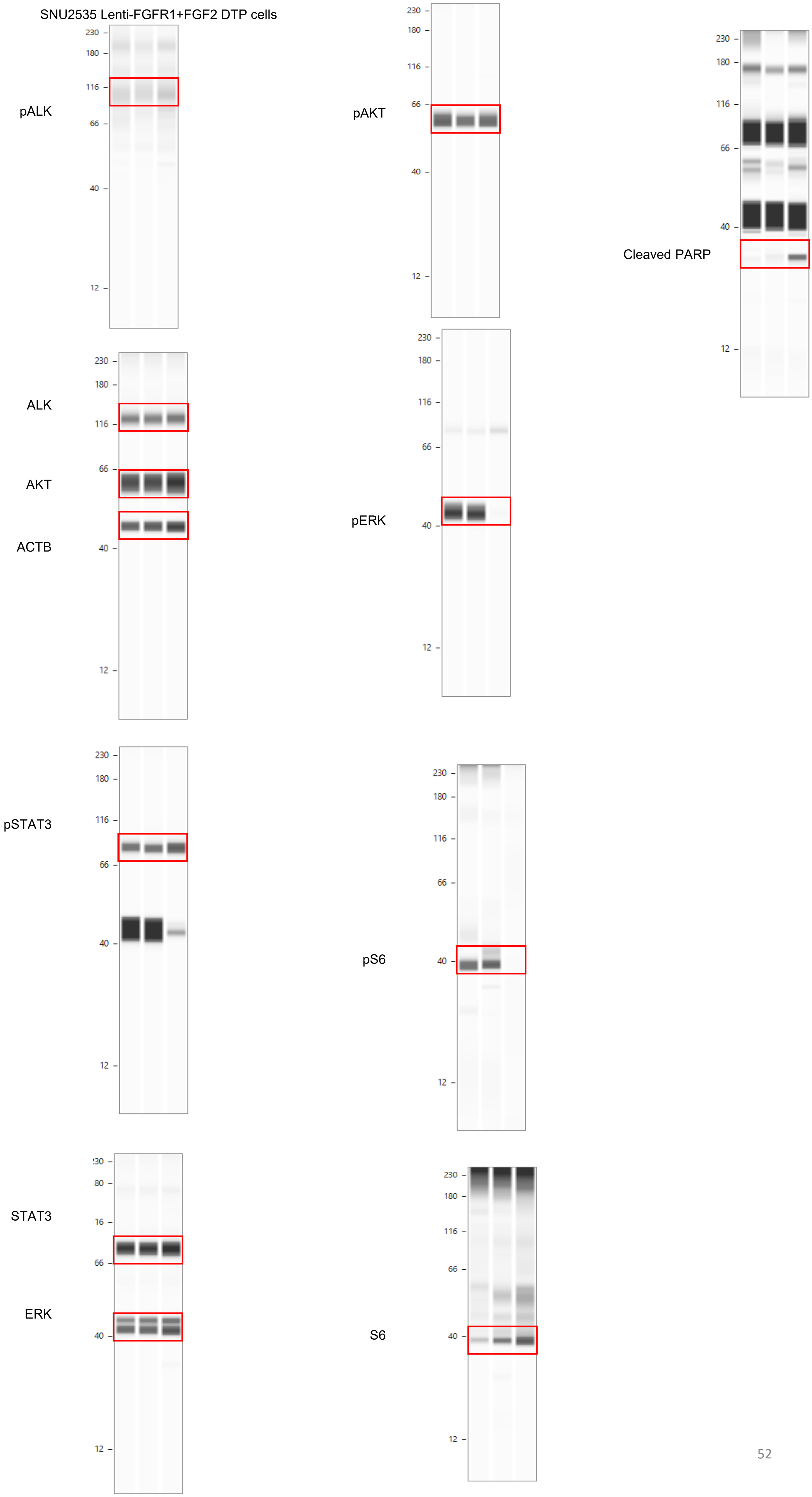
SNU2535 Lenti-FGFR1+FGF2 DTP cells
pALK
ALK
AKT
ACTB
pSTAT3
STAT3
ERK
pAKT
pERK
pS6
S6
Cleaved PARP

Supplementary Figure 13 of uncropped immunoblots blots of Supplementary Figure 4j

FGFR1

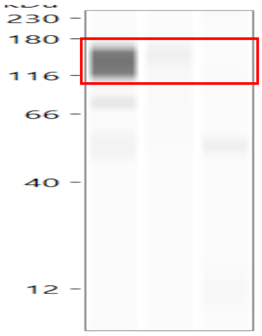

FGF2

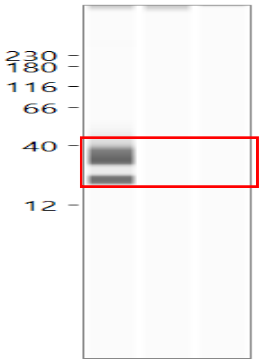

ACTB

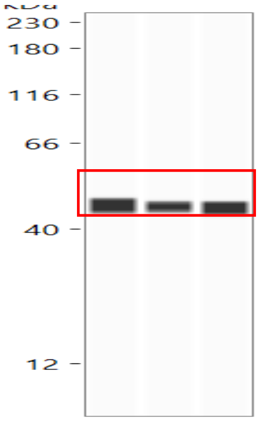

Supplementary Figure 13 of uncropped immunoblots blots of Supplementary Figure 4k

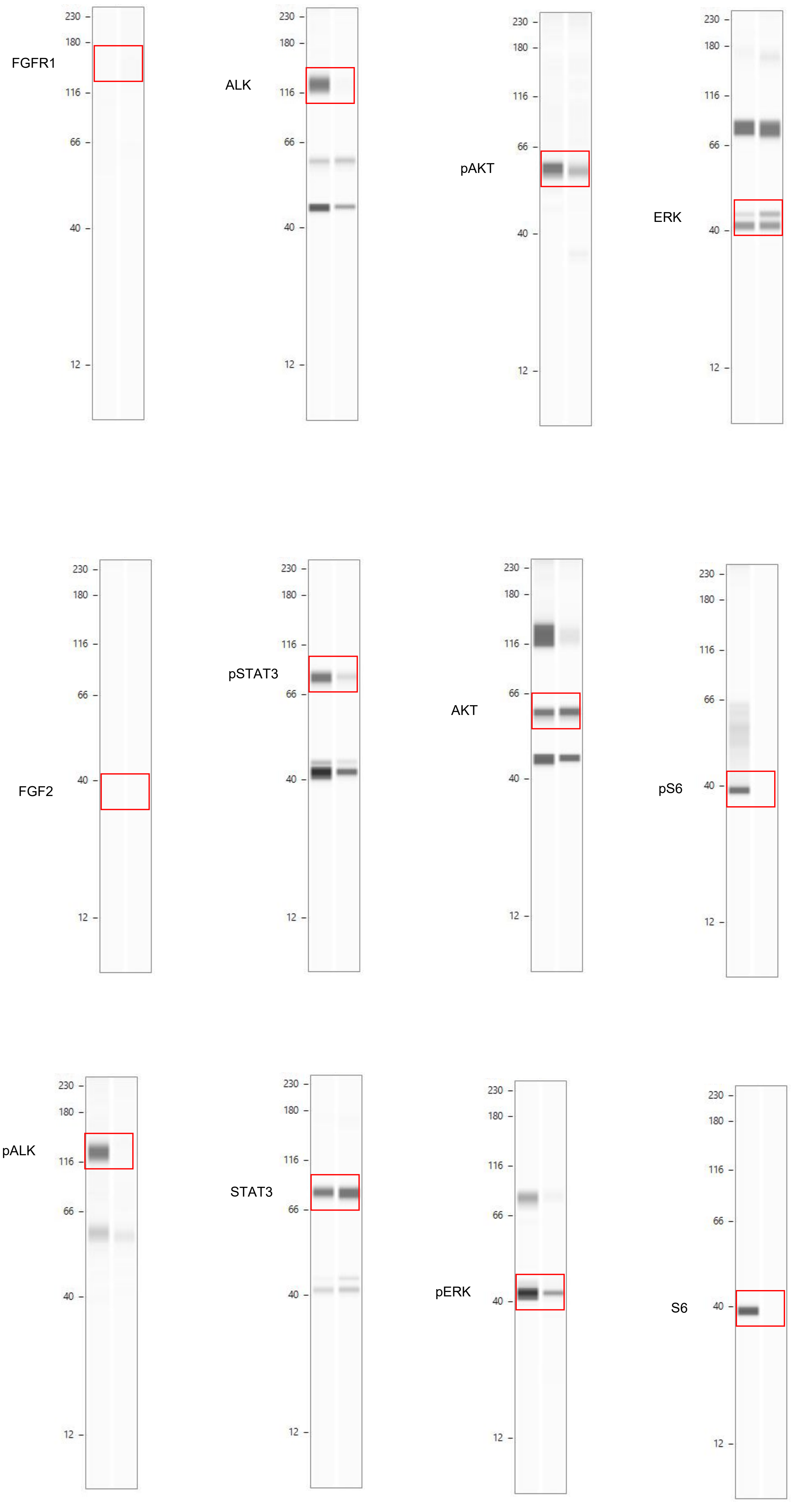

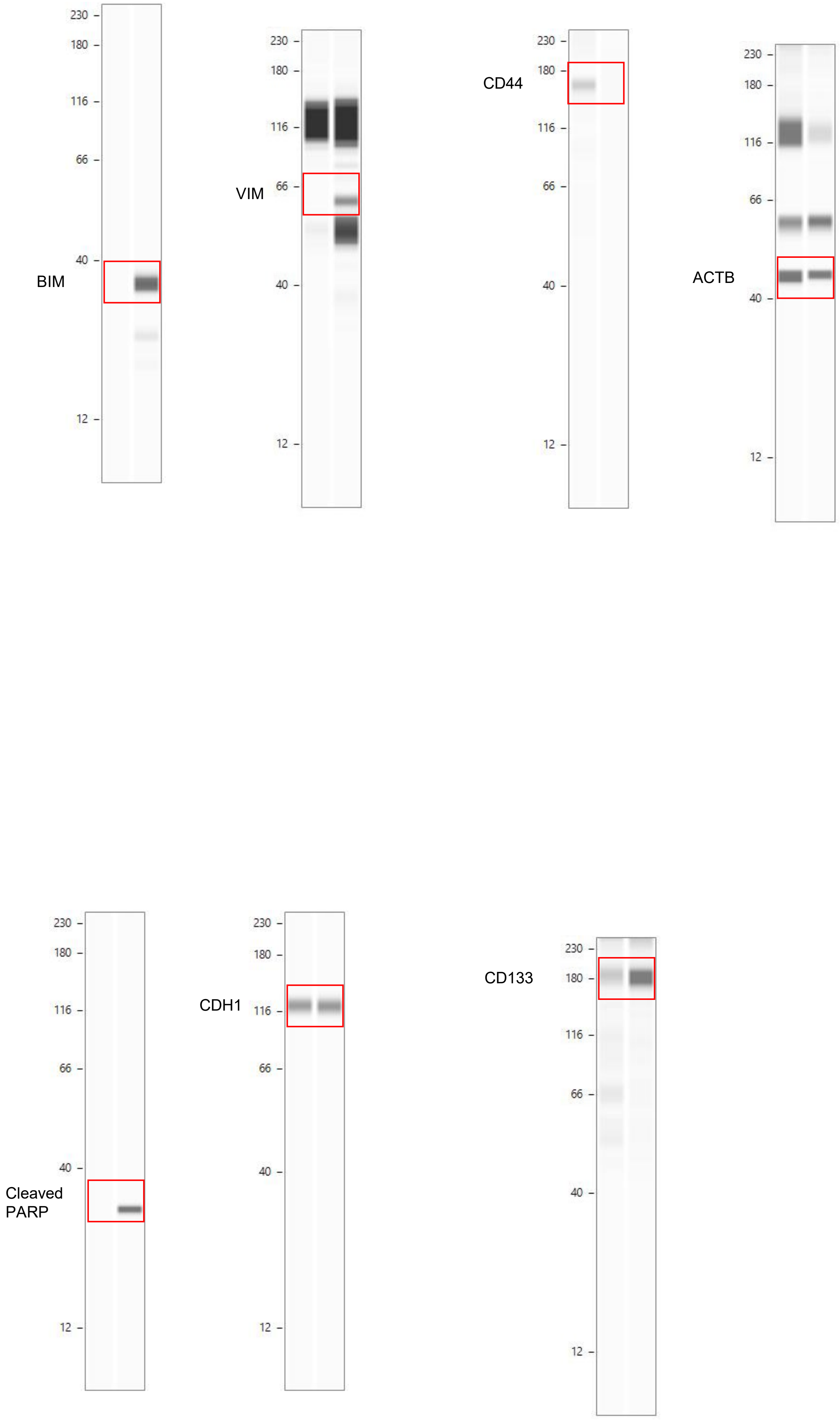
BIM
VIM
CD44
ACTB
Cleaved PARP
CDH1
CD133
230
180
116
66
40
12

Supplementary Figure 13 of uncropped immunoblots blots of Supplementary Figure 4m

Parental cells

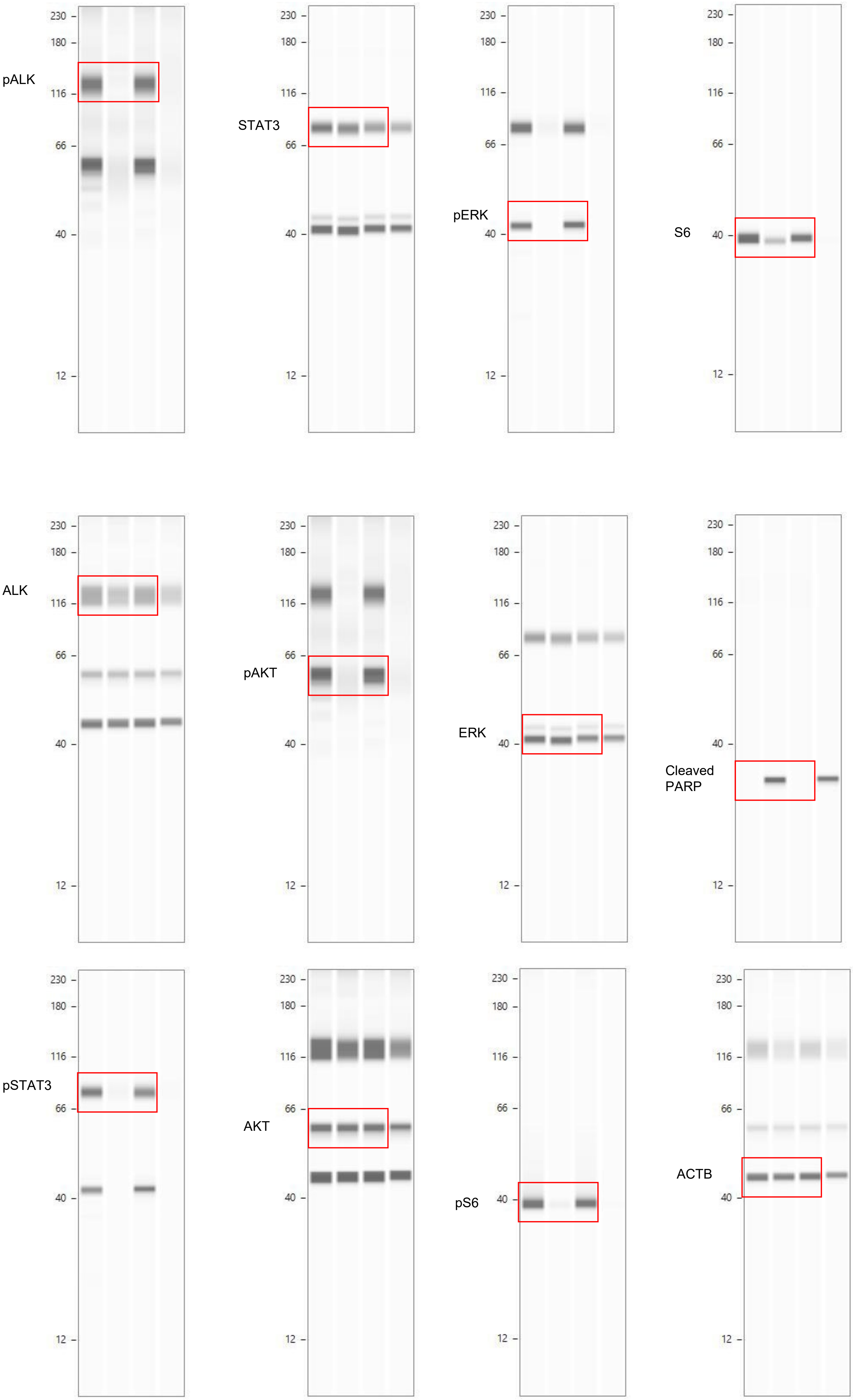

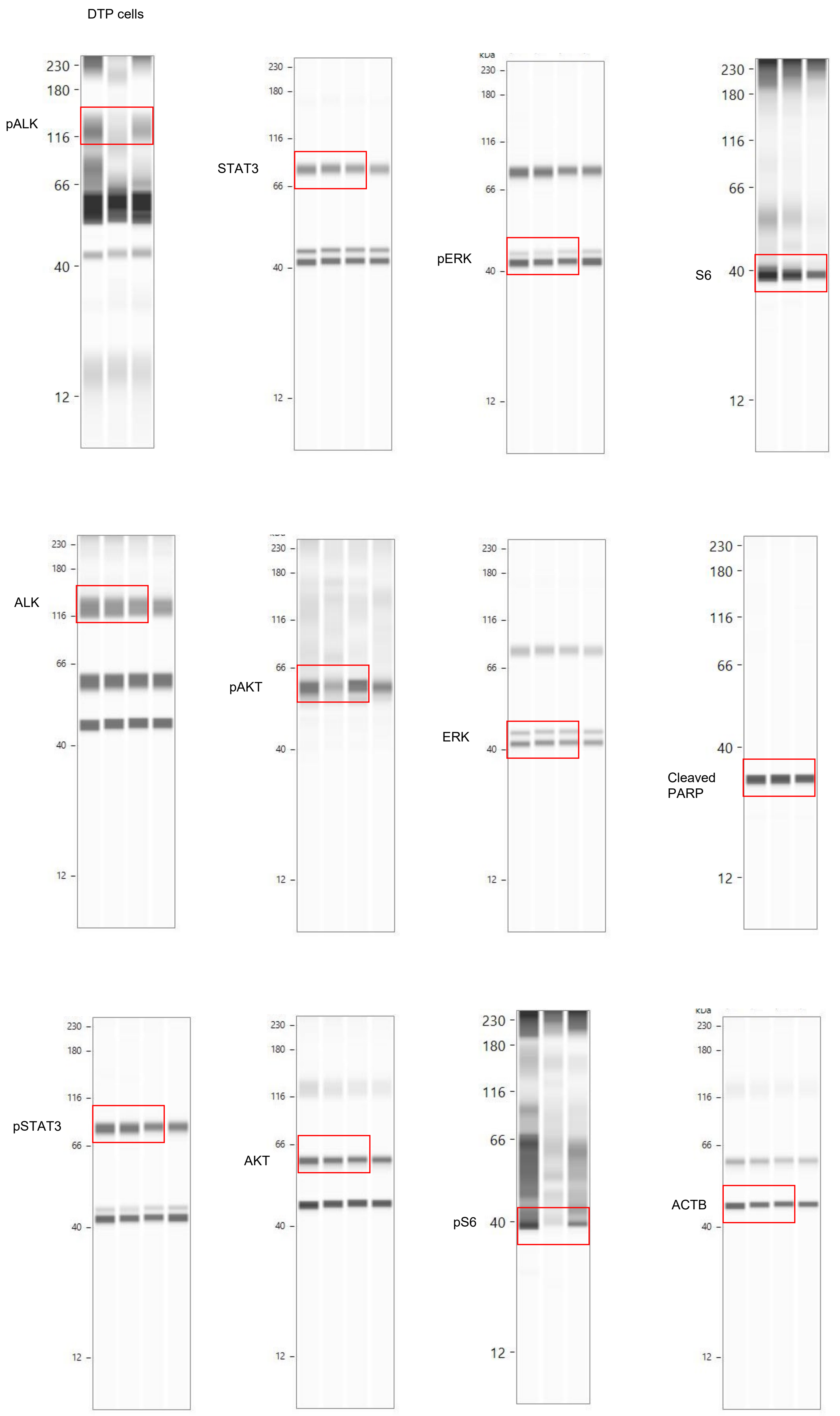
DTP cells
pALK
STAT3
pERK
S6
ALK
pAKT
ERK
Cleaved PARP
pSTAT3
AKT
pS6
ACTB
kDa
230
180
116
66
40
12

Supplementary Figure 13 of uncropped immunoblots blots of Supplementary Figure 7d

FGFR1^{high} and FGF2^{high} NCI-H1650 cells

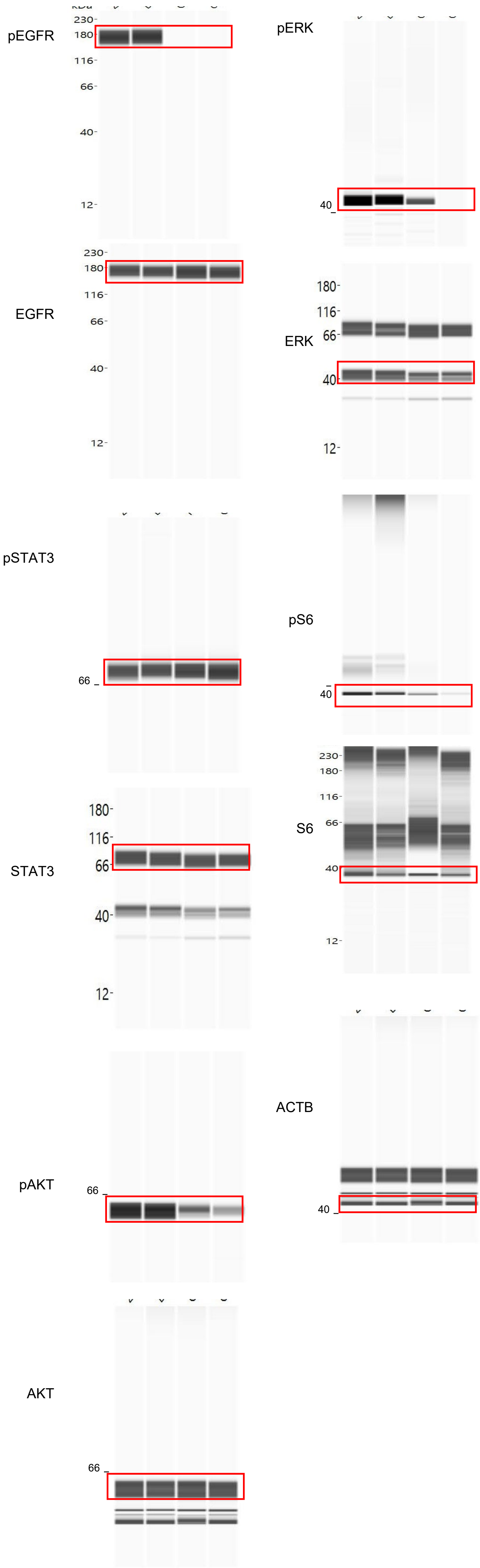

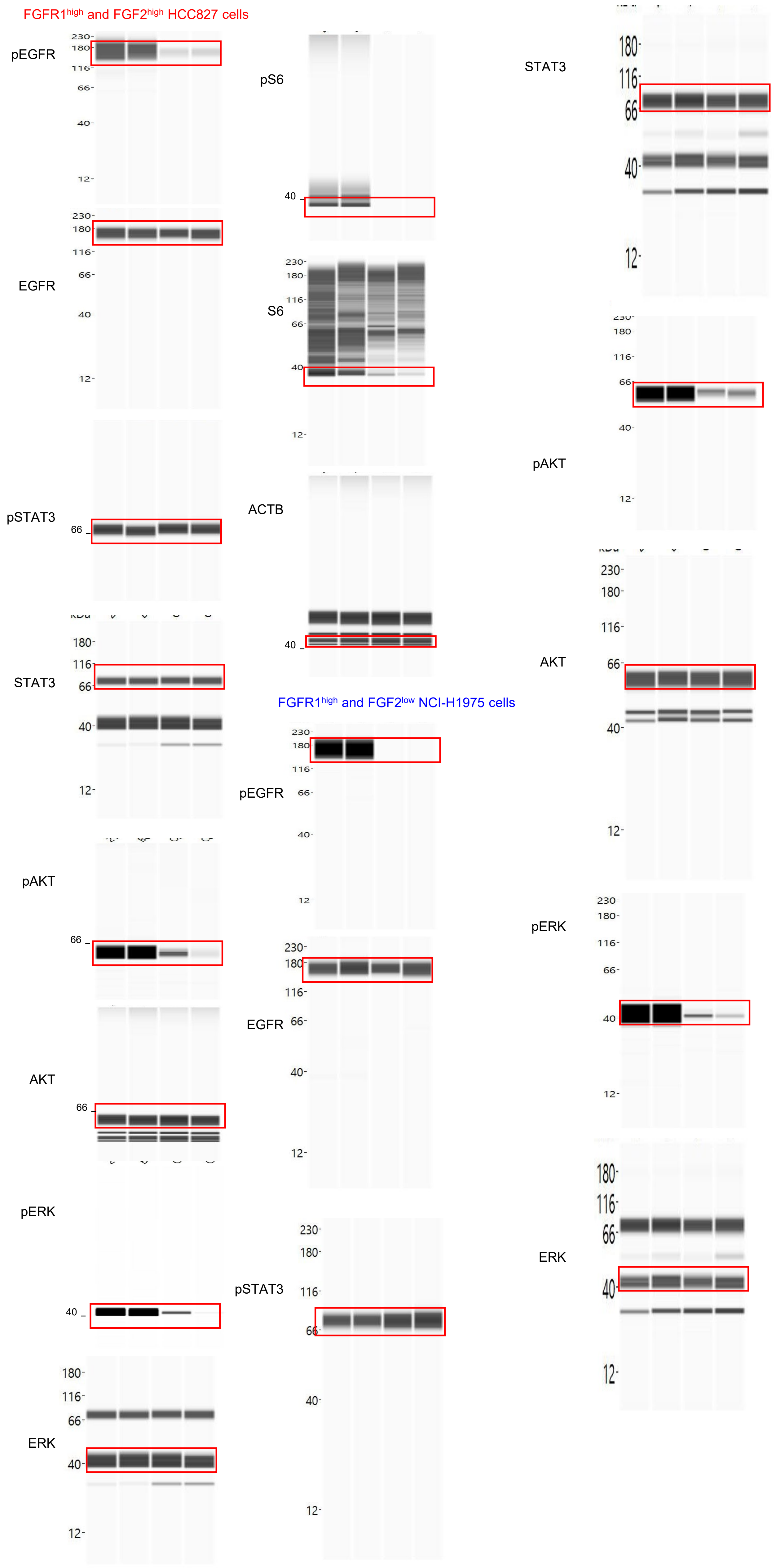
FGFR1high and FGF2high HCC827 cells
pEGFR
EGFR
pSTAT3
STAT3
pAKT
AKT
pERK
ERK
pS6
S6
ACTB
FGFR1high and FGF2low NCI-H1975 cells
pEGFR
EGFR
pSTAT3
STAT3
pAKT
AKT
pERK
ERK

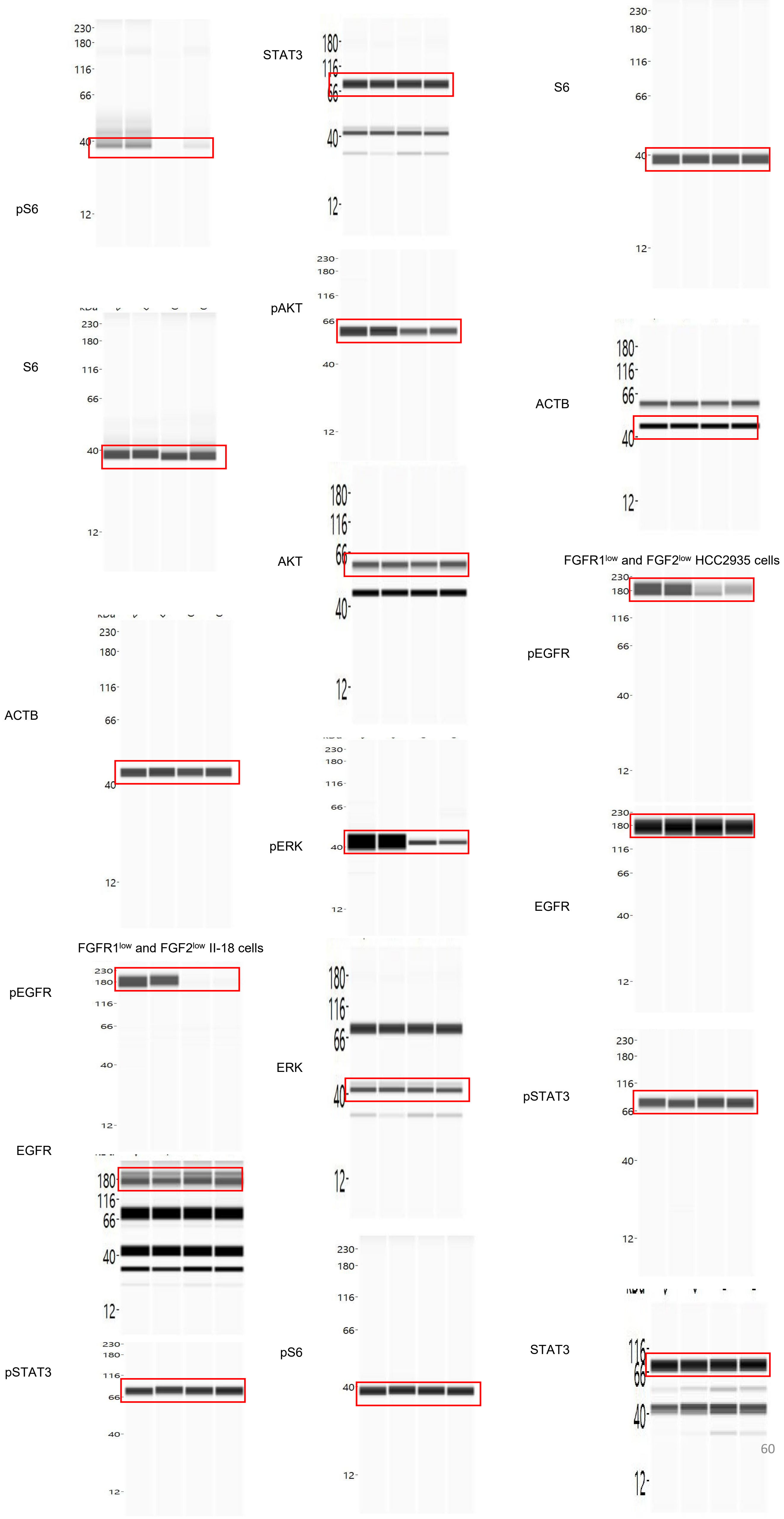
pS6
STAT3
S6
S6
pAKT
ACTB
AKT
FGFR1low and FGF2low HCC2935 cells
ACTB
pEGFR
pERK
EGFR
FGFR1low and FGF2low II-18 cells
pEGFR
ERK
pSTAT3
EGFR
pSTAT3
pS6
STAT3

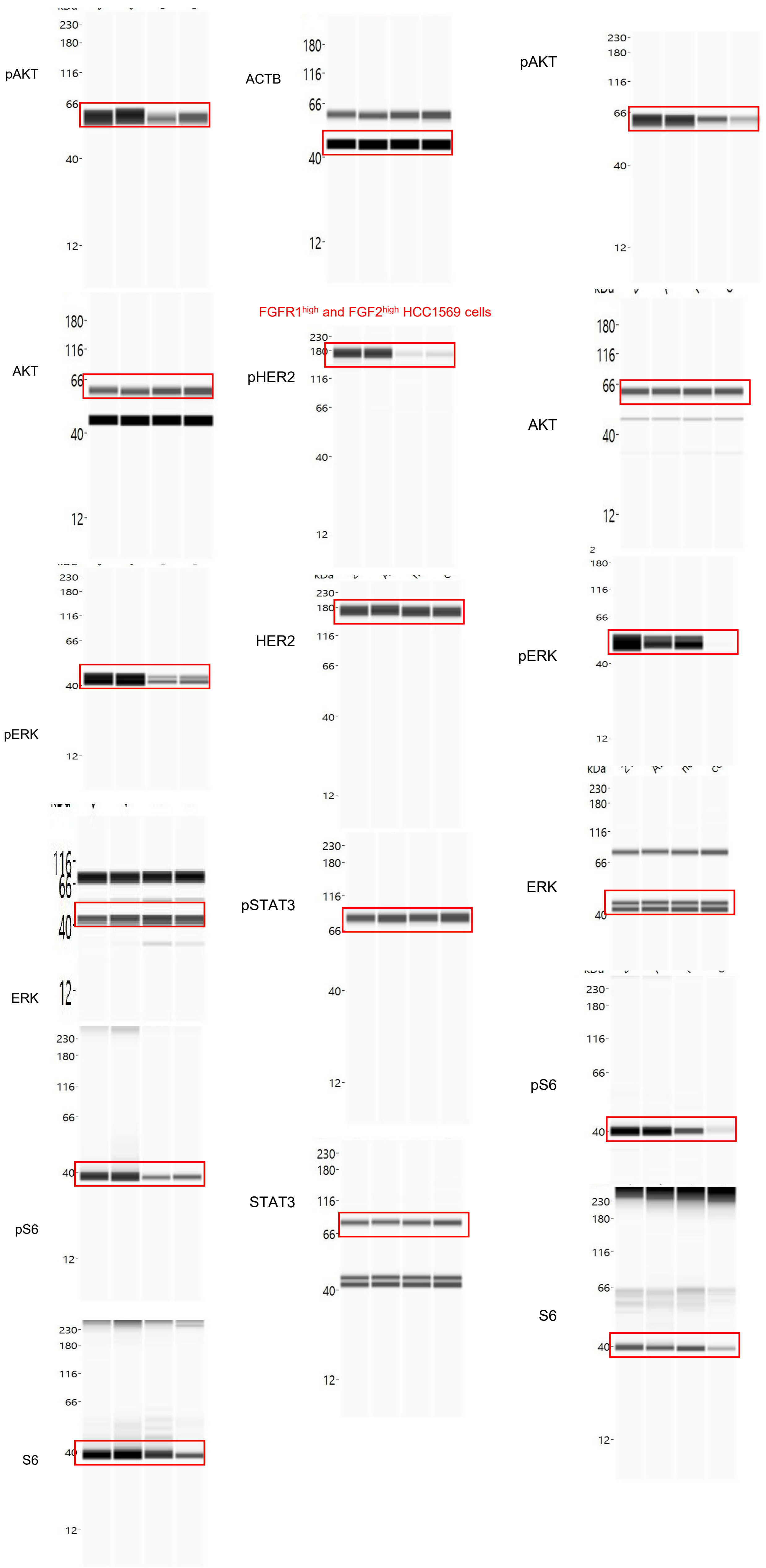
FGFR1high and FGF2high HCC1569 cells
pAKT
ACTB
pAKT
AKT
pHER2
AKT
pERK
HER2
pERK
ERK
pSTAT3
ERK
pS6
STAT3
pS6
S6
S6

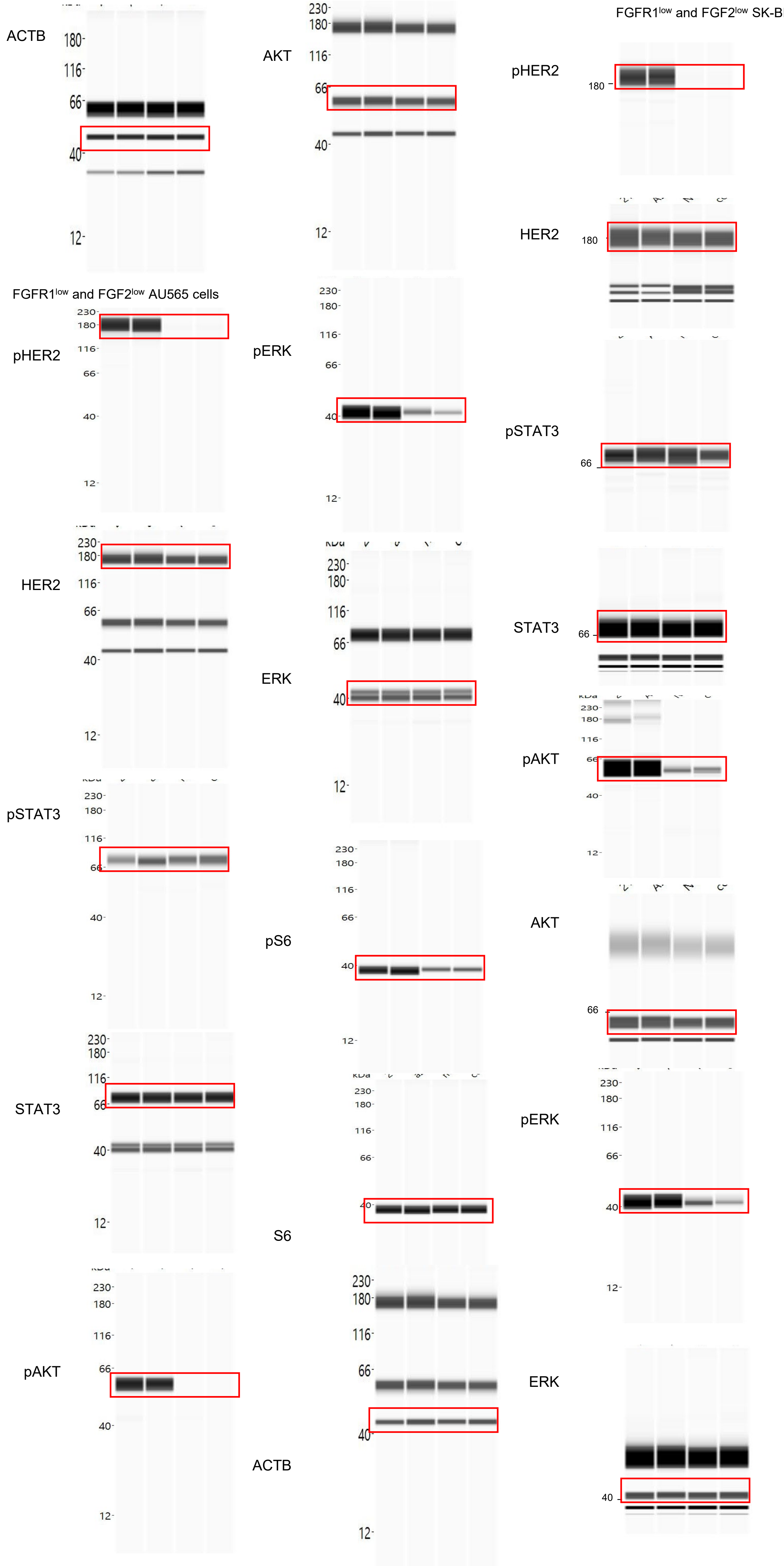
FGFR1low and FGF2low AU565 cells
ACTB
AKT
pHER2
pERK
HER2
ERK
pSTAT3
pS6
STAT3
S6
pAKT
ACTB
FGFR1low and FGF2low SK-BR-3 cells
pHER2
HER2
pSTAT3
STAT3
pAKT
AKT
pERK
ERK

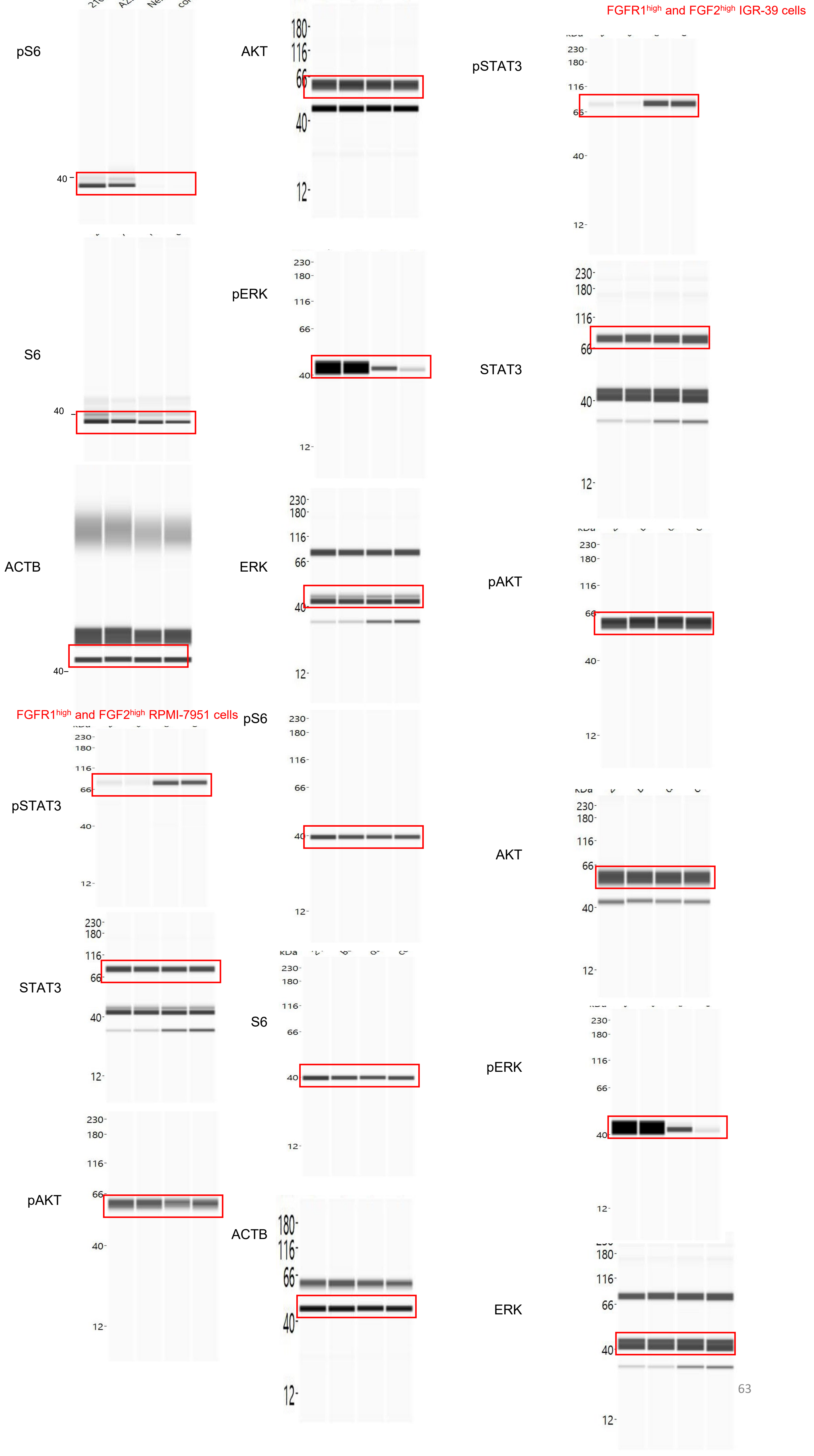
pS6
S6
ACTB
AKT
pERK
ERK
pS6
S6
ACTB
FGFR1high and FGF2high RPMI-7951 cells
pSTAT3
STAT3
pAKT
FGFR1high and FGF2high IGR-39 cells
pSTAT3
STAT3
pAKT
AKT
pERK
ERK

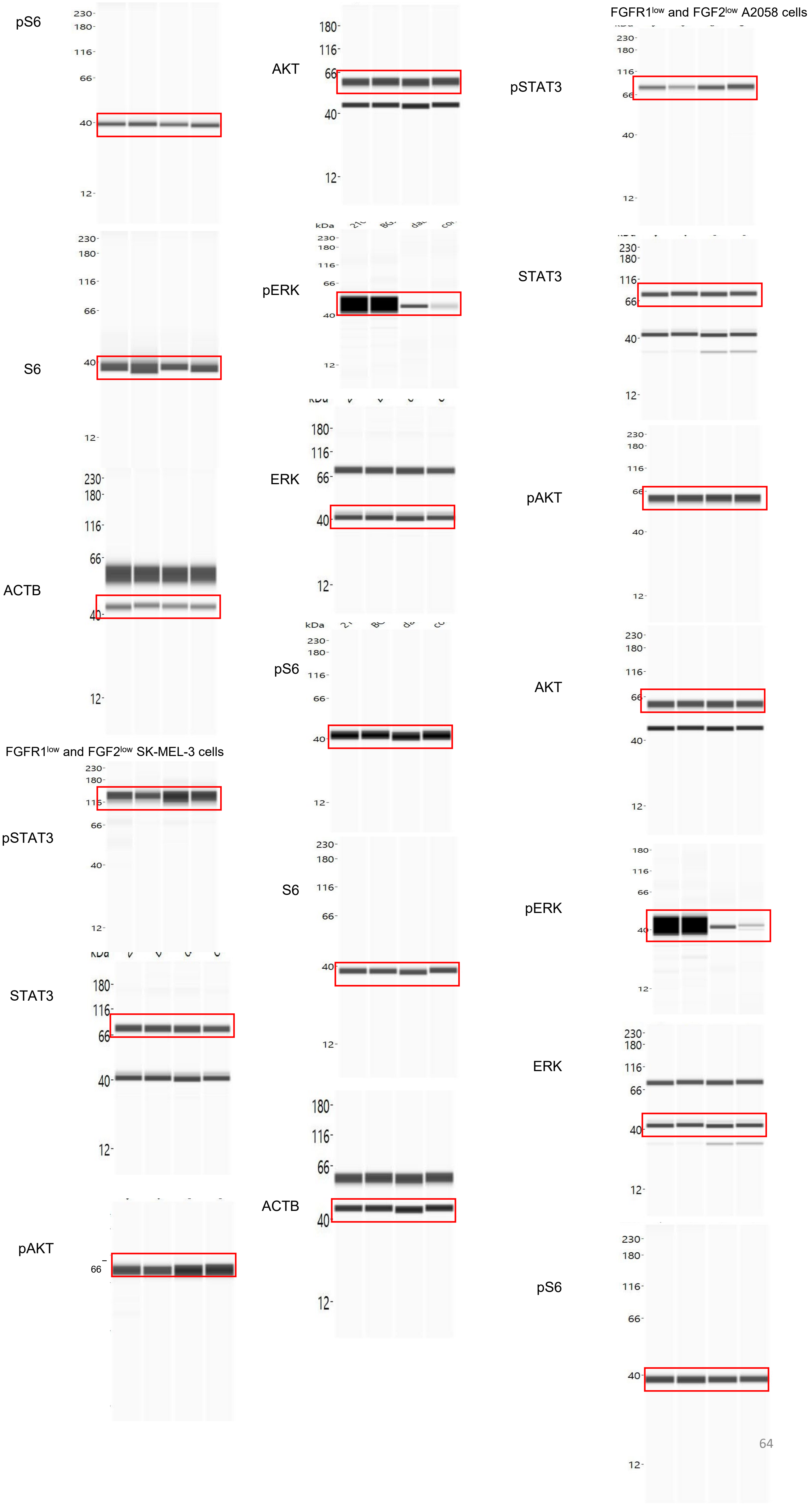
pS6
AKT
FGFR1low and FGF2low A2058 cells
pSTAT3
S6
pERK
STAT3
ACTB
ERK
pAKT
pS6
AKT
FGFR1low and FGF2low SK-MEL-3 cells
pSTAT3
S6
pERK
STAT3
ERK
ACTB
pAKT
pS6

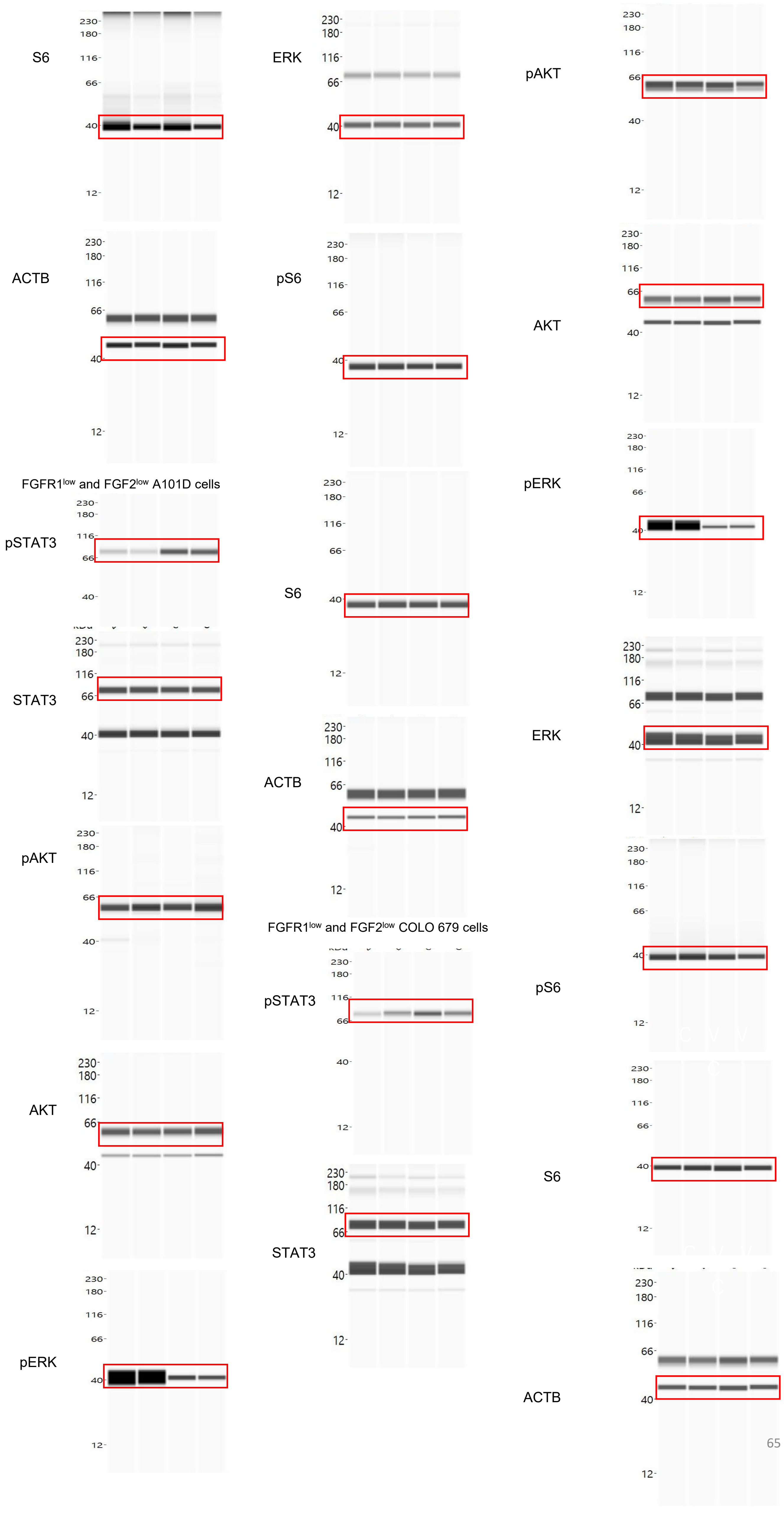
S6
ERK
pAKT
ACTB
pS6
AKT
pERK
FGFR1low and FGF2low A101D cells
pSTAT3
S6
STAT3
ERK
ACTB
pAKT
FGFR1low and FGF2low COLO 679 cells
pS6
pSTAT3
AKT
S6
STAT3
ACTB
pERK

Supplementary Figure 13 of uncropped immunoblots blots of Supplementary Figure 7g

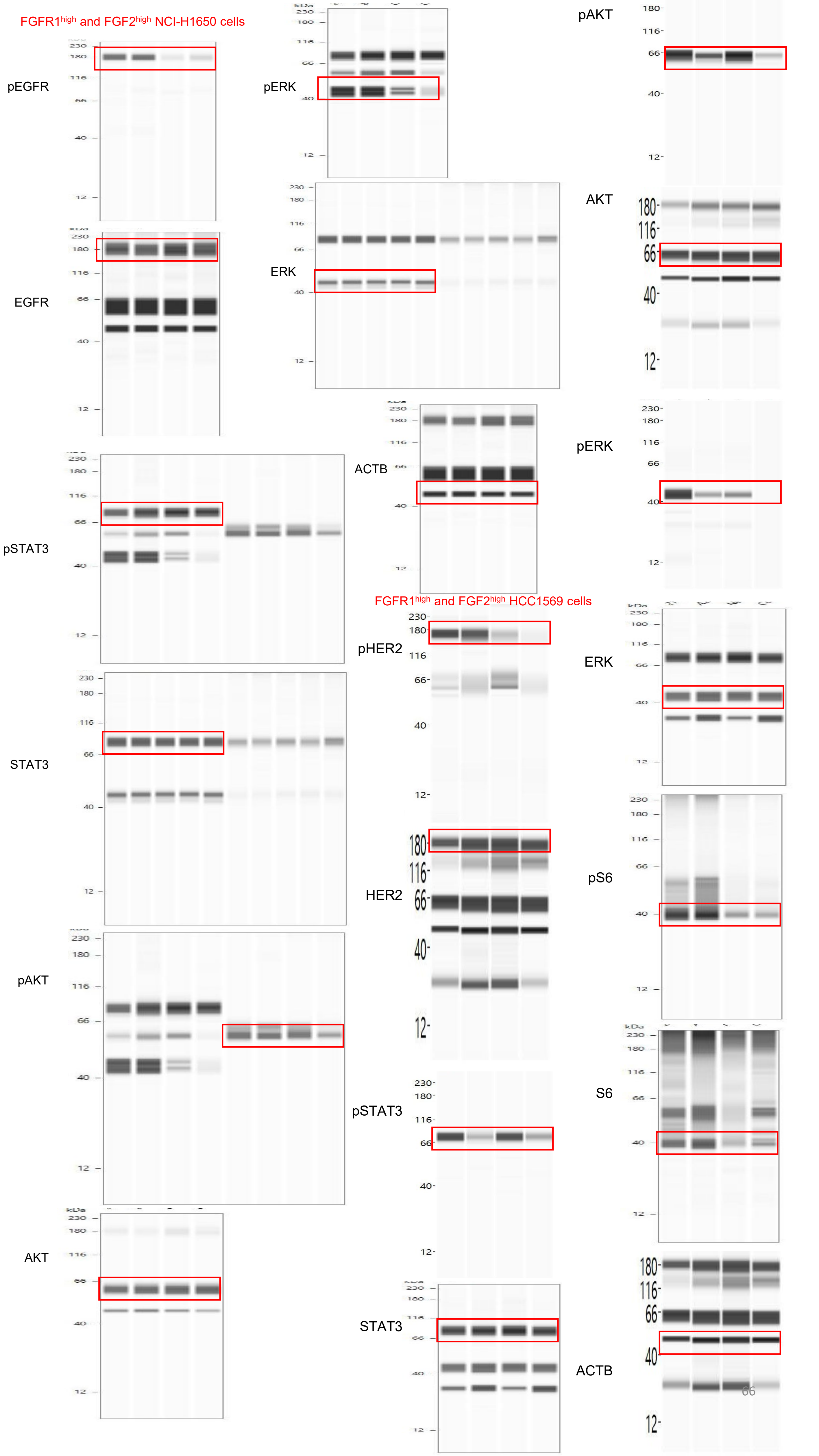

Supplementary Figure 13 of uncropped immunoblots blots of Supplementary Figure 7i

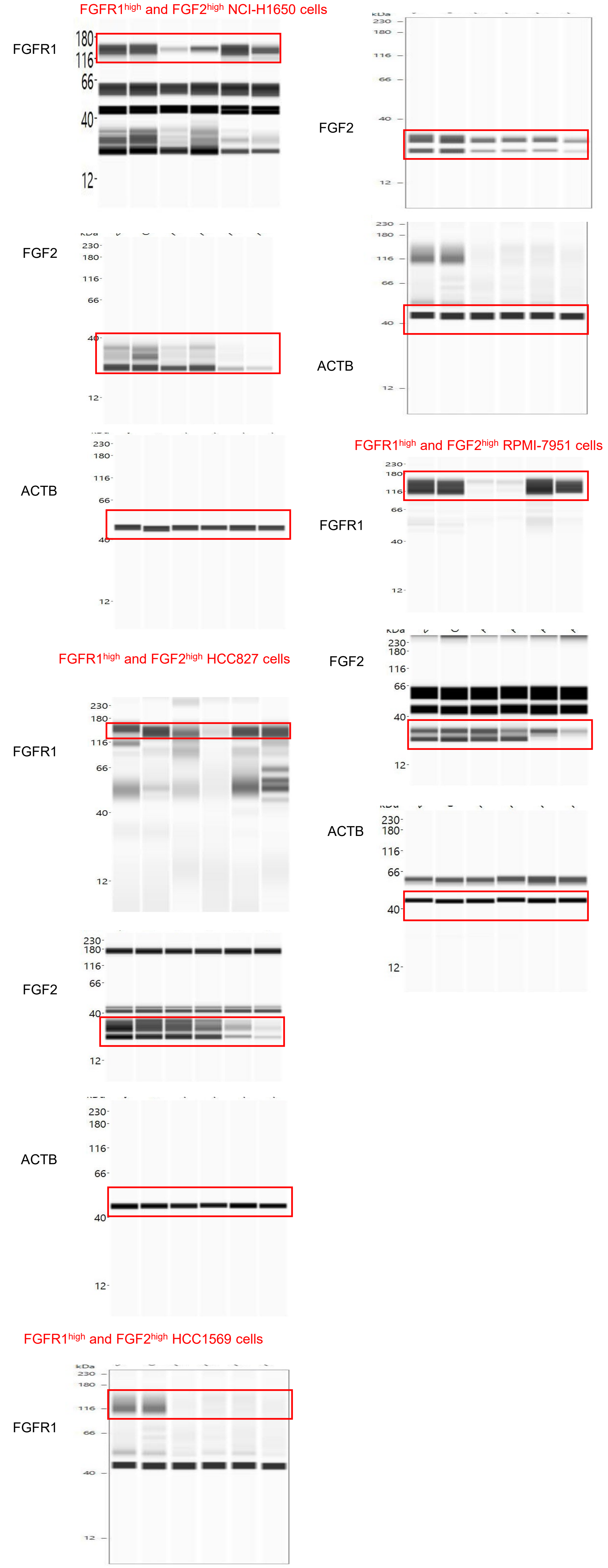

Supplementary Figure 13 of uncropped immunoblots blots of Supplementary Figure 8b

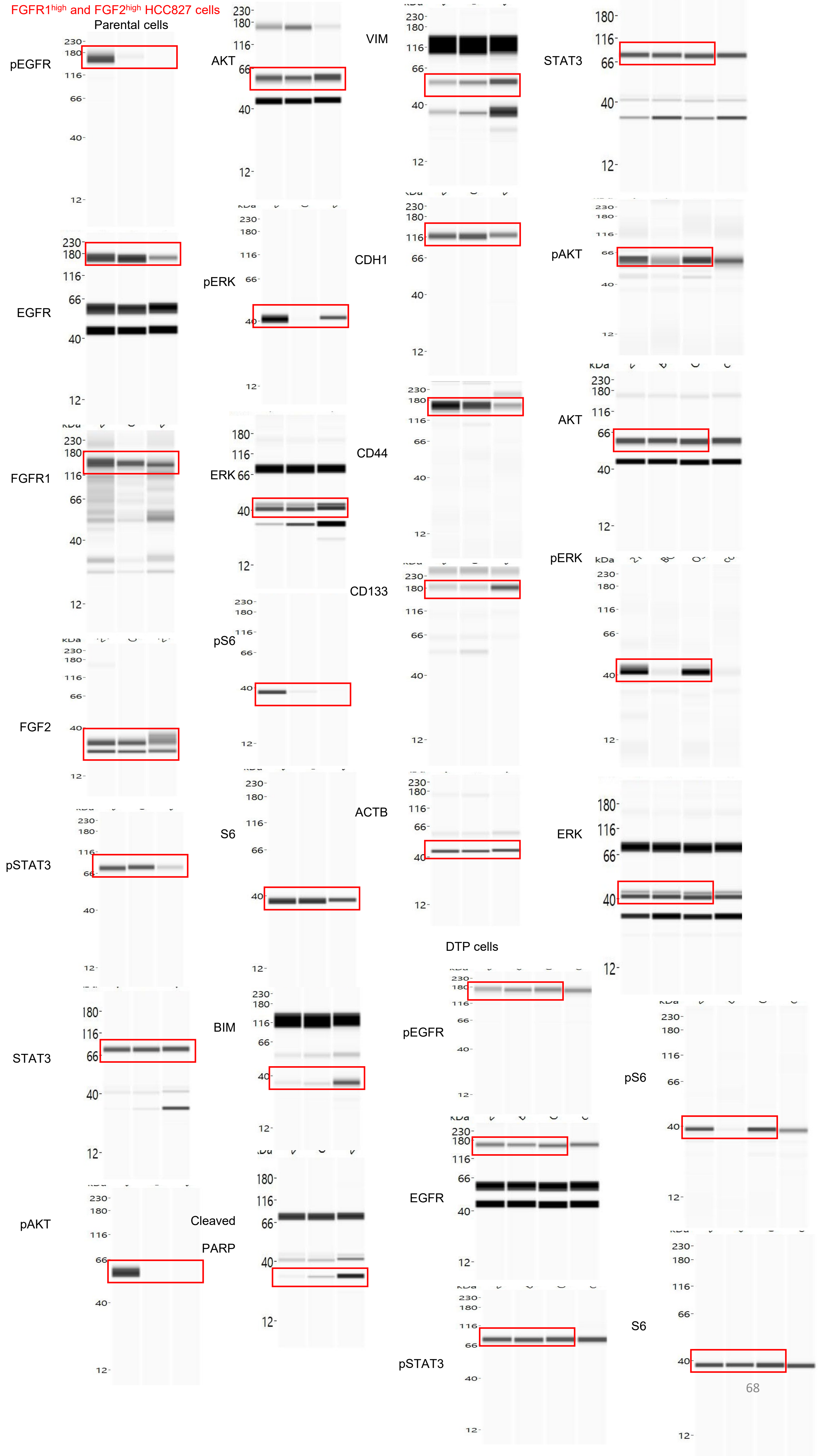

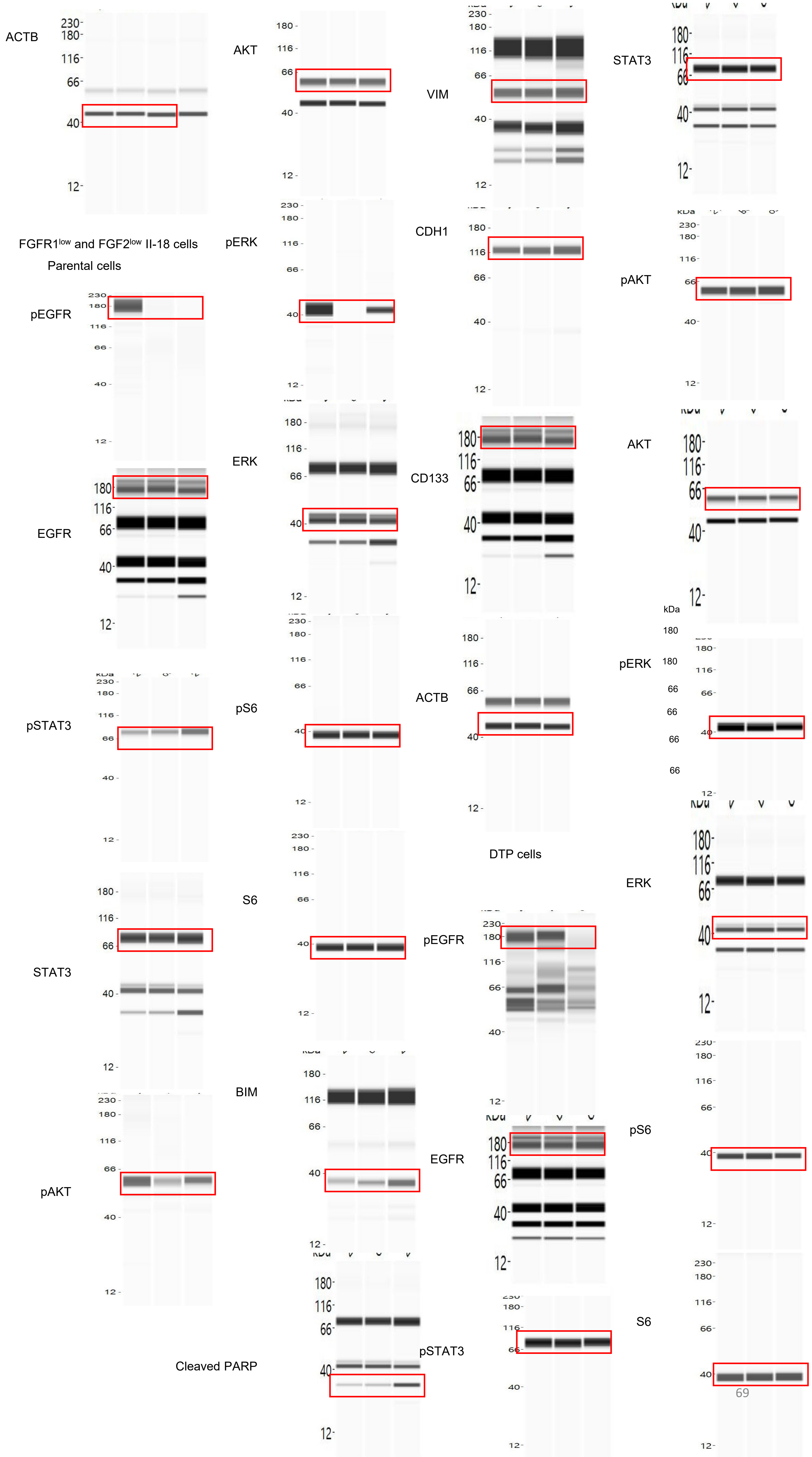

ACTB
AKT
VIM
STAT3
FGFR1low and FGF2low II-18 cells
Parental cells
pERK
CDH1
pAKT
pEGFR
ERK
CD133
AKT
EGFR
pERK
pS6
ACTB
pSTAT3
S6
DTP cells
ERK
STAT3
pEGFR
BIM
pS6
EGFR
pAKT
S6
Cleaved PARP
pSTAT3
kDa

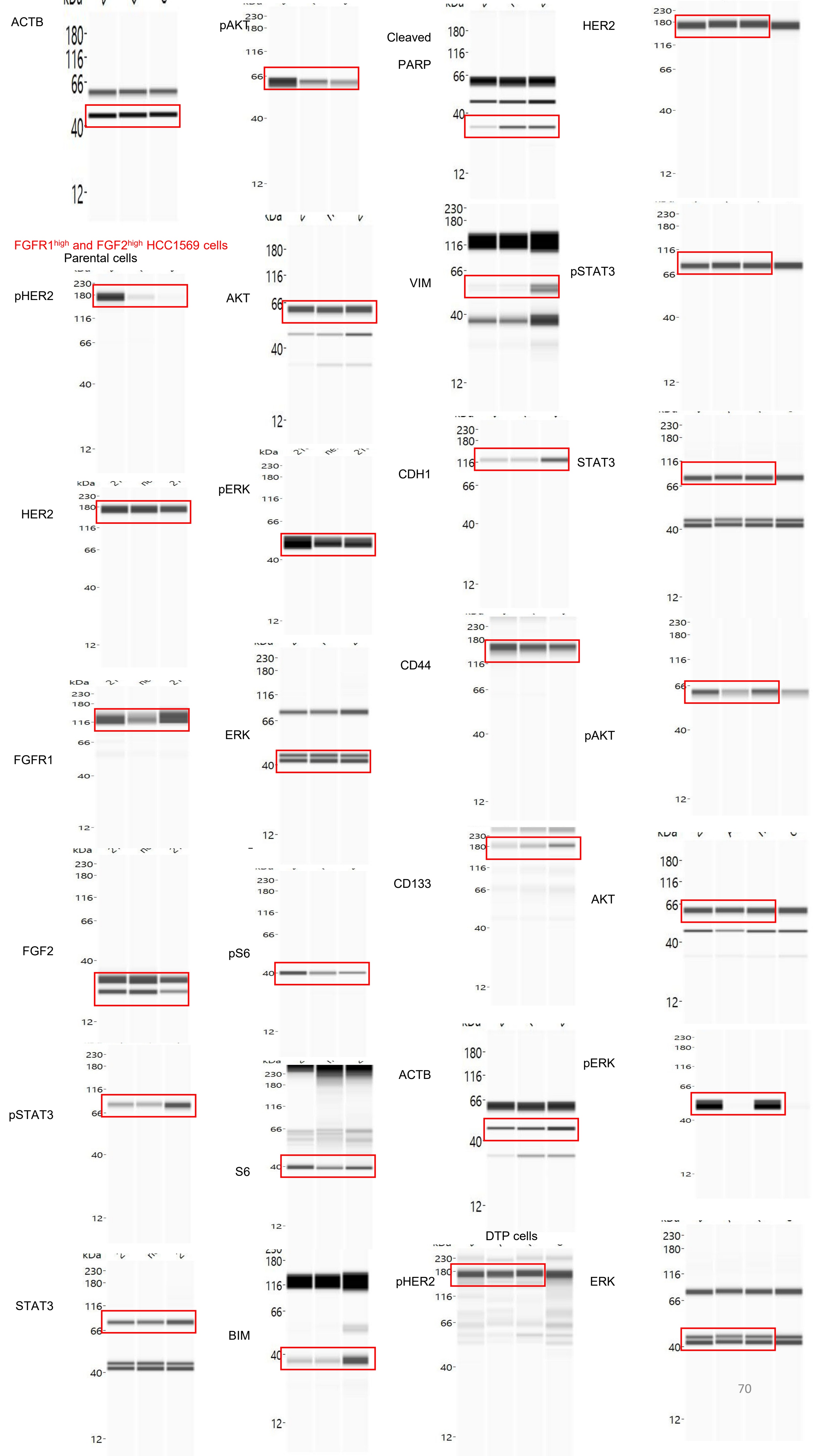
FGFR1high and FGF2high HCC1569 cells
Parental cells
DTP cells
ACTB
pAKT
Cleaved PARP
HER2
pHER2
AKT
VIM
pSTAT3
HER2
pERK
CDH1
STAT3
FGFR1
ERK
CD44
pAKT
FGF2
pS6
CD133
AKT
pSTAT3
S6
ACTB
pERK
STAT3
BIM
pHER2
ERK
kDa
230
180
116
66
40
12

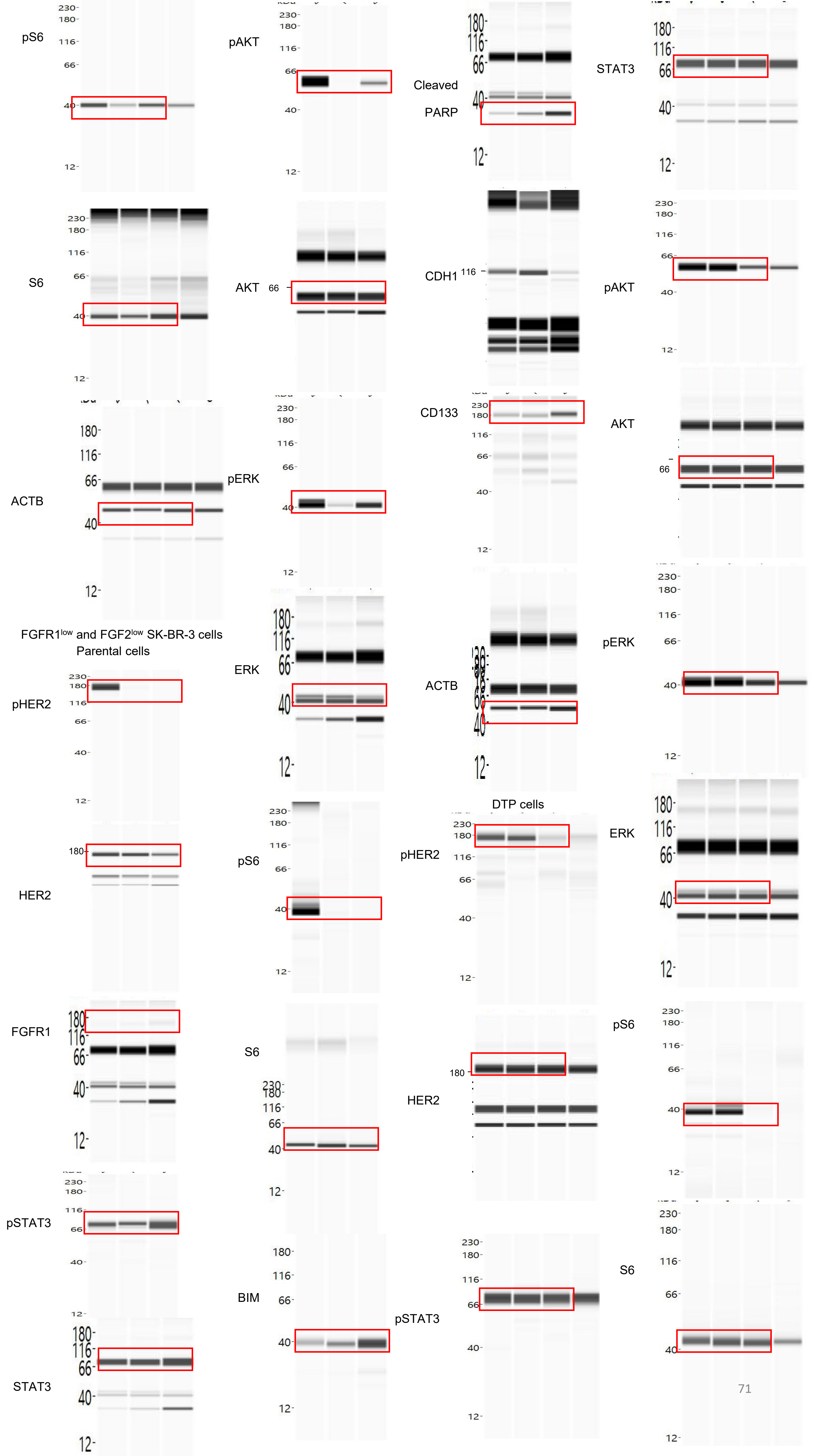
pS6
pAKT
Cleaved
PARP
STAT3
S6
AKT
CDH1
pAKT
ACTB
pERK
CD133
AKT
FGFR1low and FGF2low SK-BR-3 cells
Parental cells
ERK
ACTB
pERK
pHER2
HER2
pS6
DTP cells
pHER2
ERK
FGFR1
S6
HER2
pS6
pSTAT3
BIM
pSTAT3
S6
STAT3

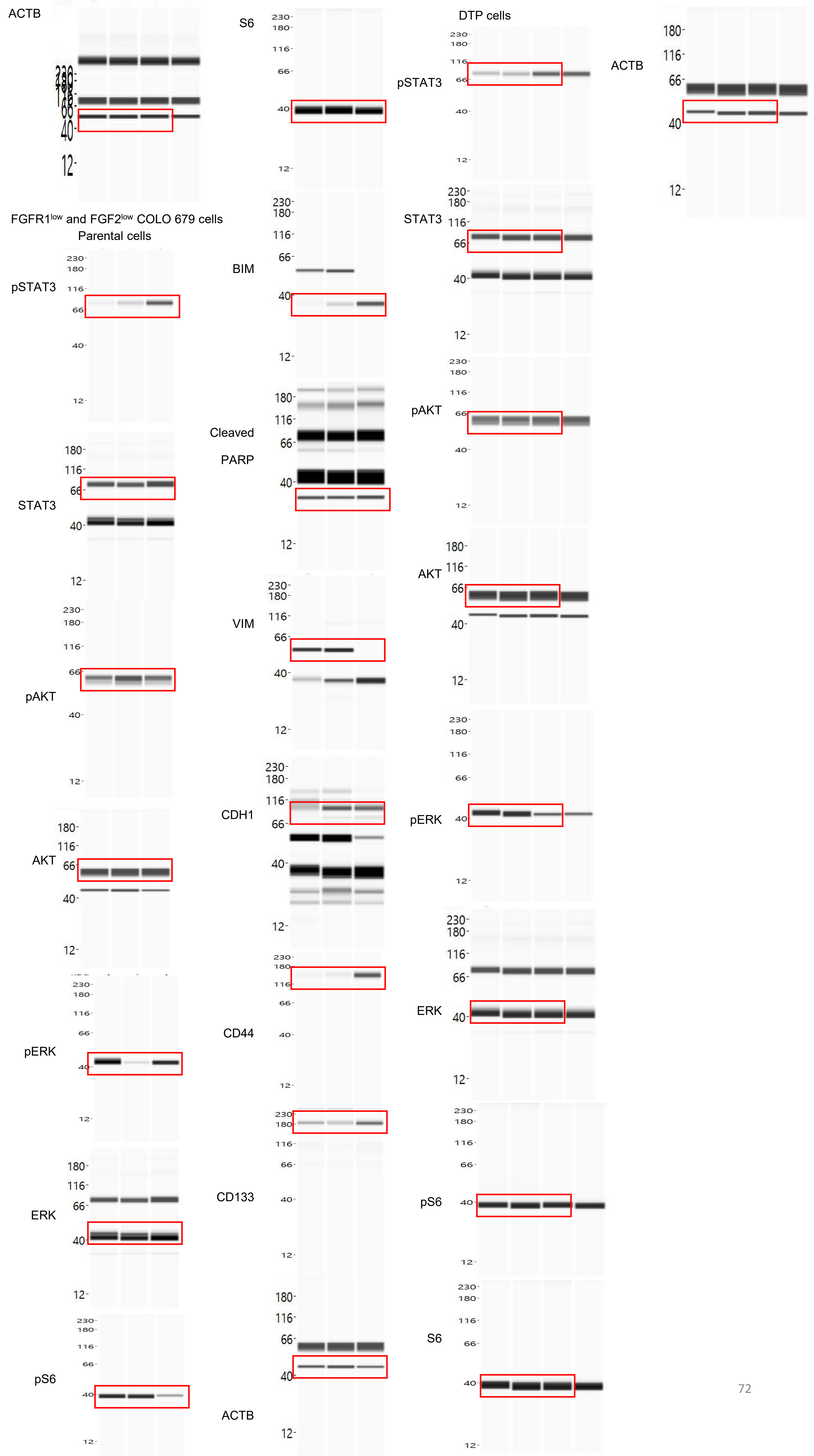
ACTB
FGFR1low and FGF2low COLO 679 cells
Parental cells
pSTAT3
STAT3
pAKT
AKT
pERK
ERK
pS6
S6
BIM
Cleaved
PARP
VIM
CDH1
CD44
CD133
ACTB
DTP cells
pSTAT3
STAT3
pAKT
AKT
pERK
ERK
pS6
S6
ACTB

Supplementary Figure 13 of uncropped immunoblots blots of Supplementary Figure 9c

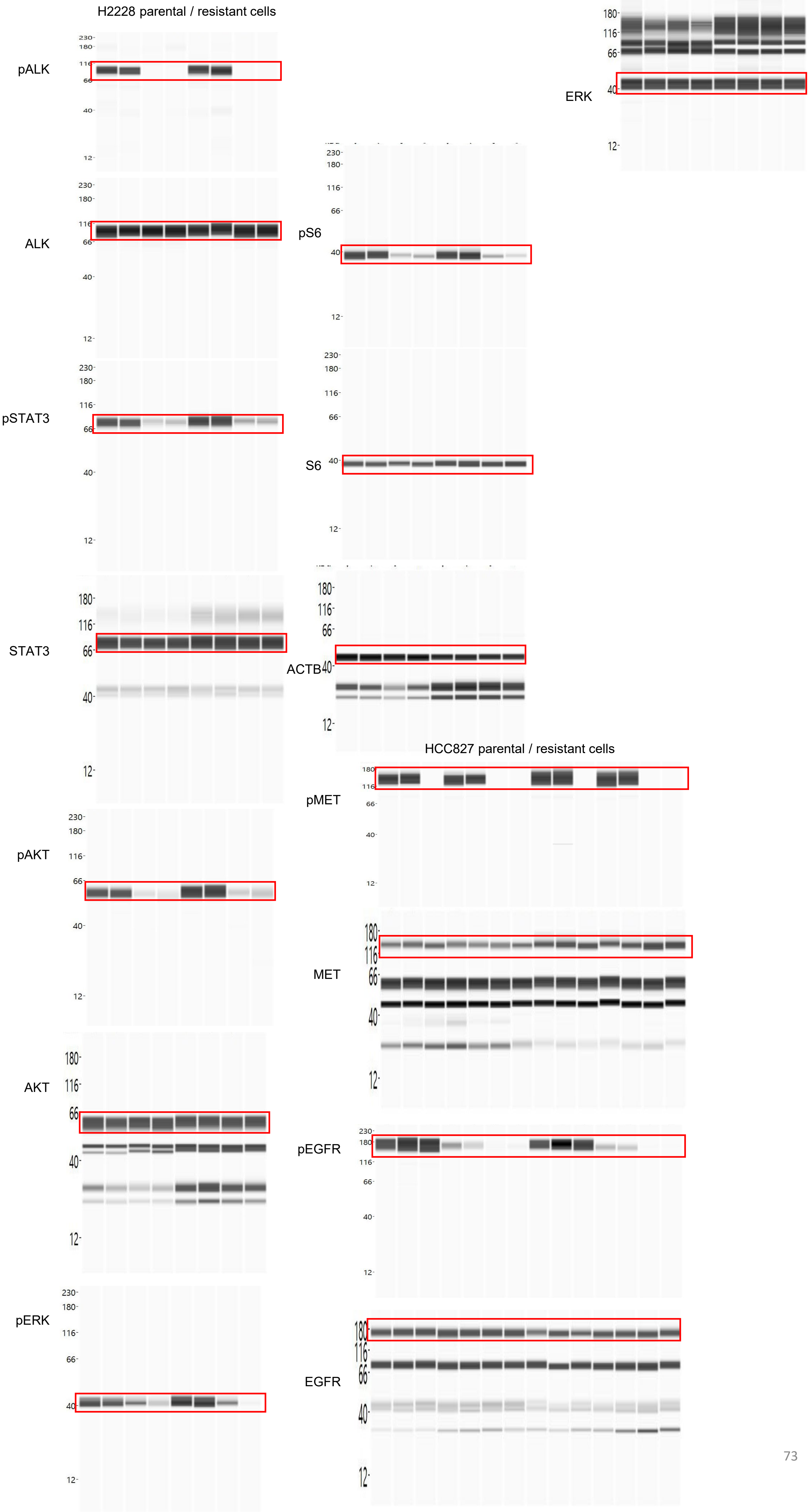

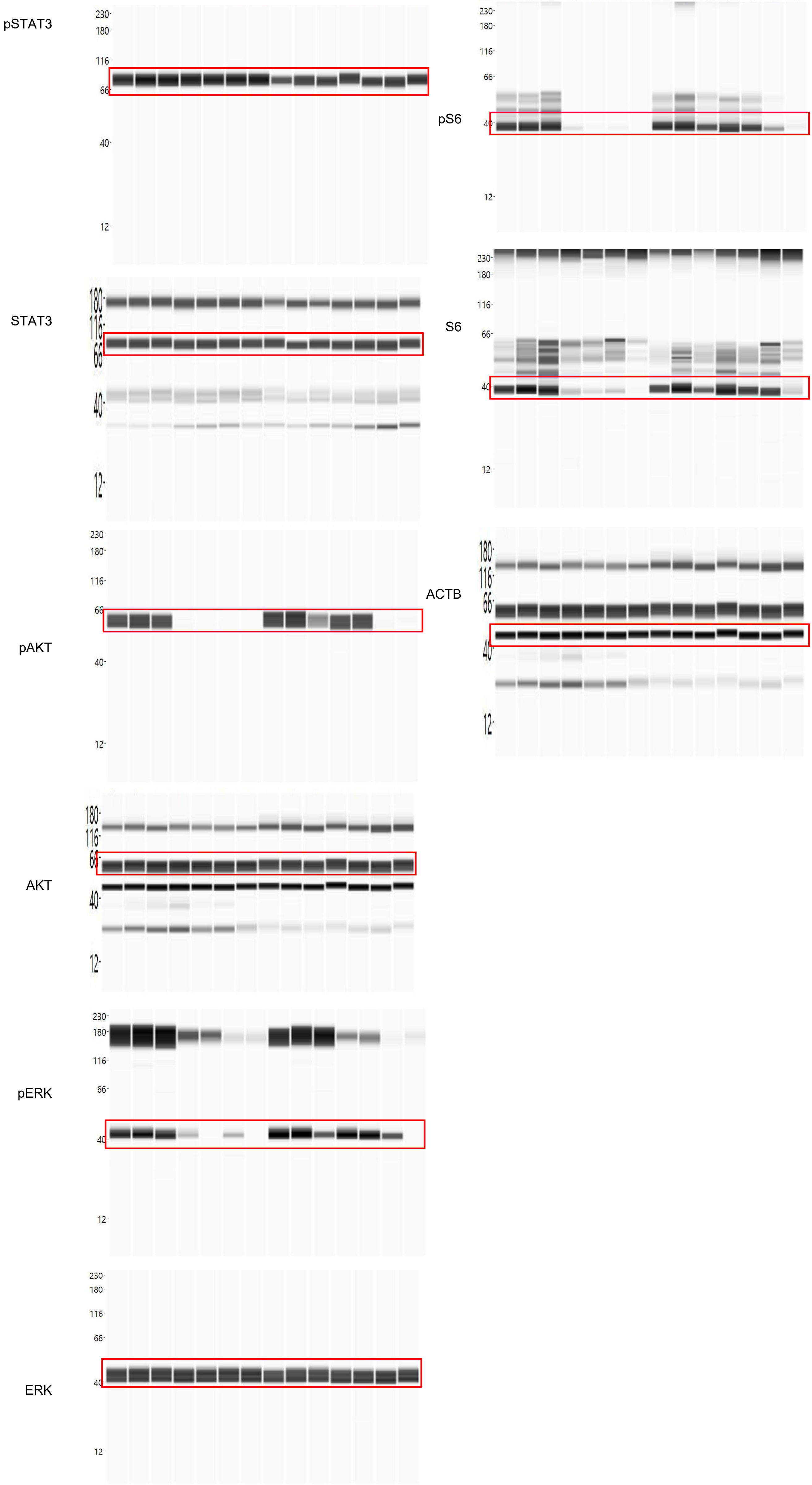
pSTAT3
pS6
STAT3
S6
pAKT
ACTB
AKT
pERK
ERK

Supplementary Figure 13 of uncropped immunoblots blots of Supplementary Figure 10a

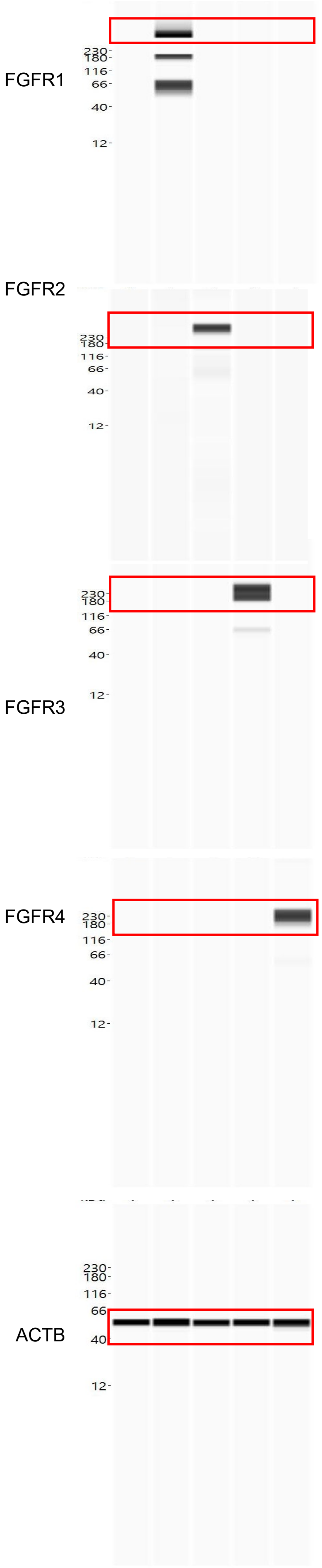

Supplementary Figure 13 of uncropped immunoblots blots of Supplementary Figure 10c

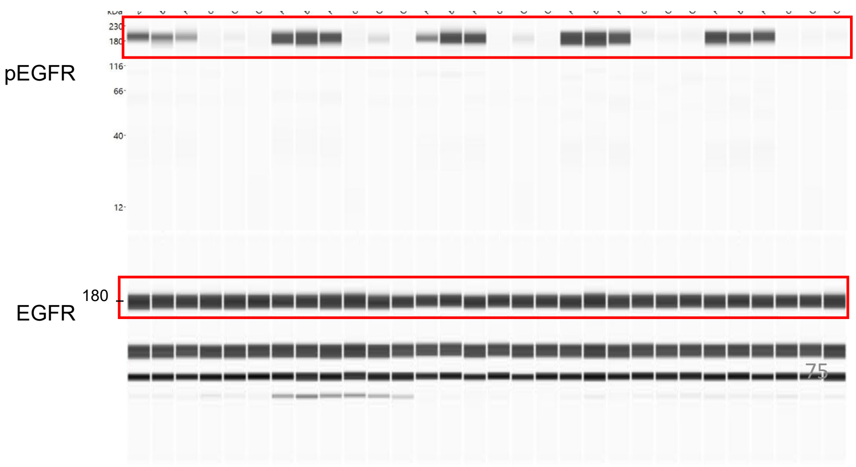

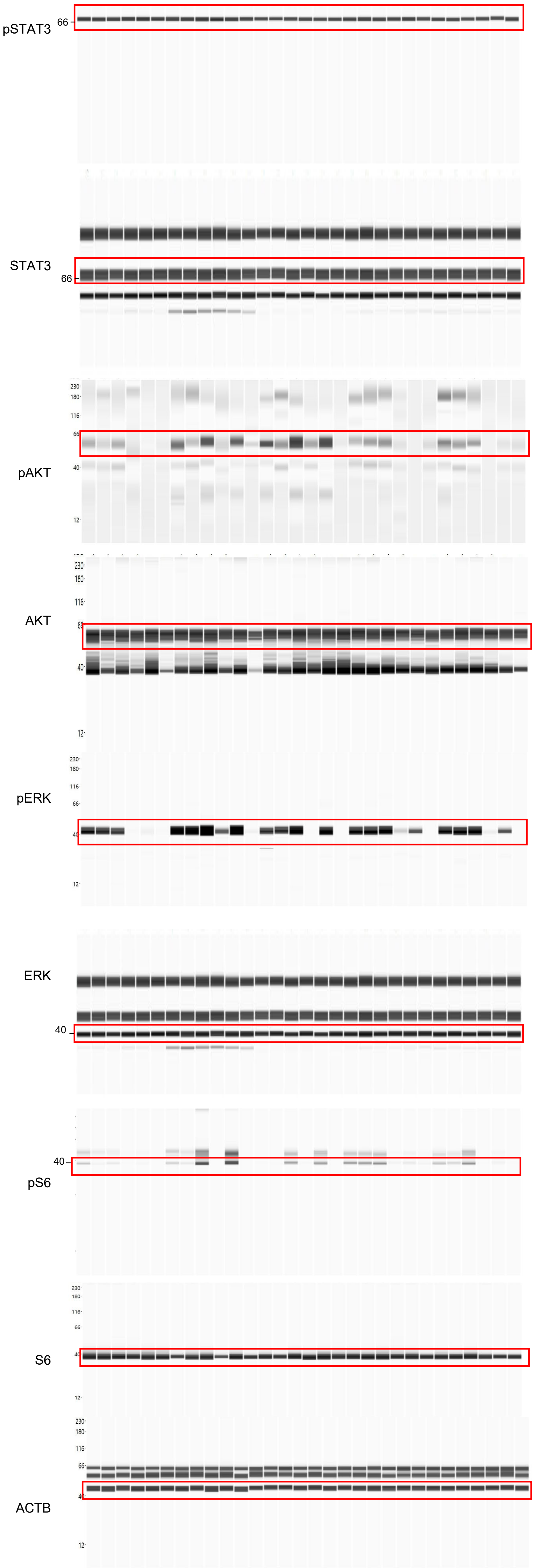
pSTAT3
66
STAT3
66
230
180
116
66
40
12
pAKT
AKT
pERK
ERK
pS6
S6
ACTB

Supplementary Figure 13. Uncropped immunoblots blots

Uncropped immunoblots blots of each Figure and Supplementary Figure are shown.
