# Supplementary material for: FGFR blockade inhibits targeted therapy-tolerant persister in basal FGFR1- and FGF2-high cancers with driver oncogenes
Source: NPJ Precis Oncol. 2023 Oct 25;7:107. doi: 10.1038/s41698-023-00462-0 (PMC10600219; doi:10.1038/s41698-023-00462-0)
Supplement: Supplementary file 3 — Reporting Summary [file 41698_2023_462_MOESM3_ESM.pdf]

nature portfolio

Corresponding author(s): Shigeki Yoshiura
Last updated by author(s): Jan 10, 2023

Reporting Summary

Nature Portfolio wishes to improve the reproducibility of the work that we publish. This form provides structure for consistency and transparency in reporting. For further information on Nature Portfolio policies, see our Editorial Policies and the Editorial Policy Checklist.

Statistics

For all statistical analyses, confirm that the following items are present in the figure legend, table legend, main text, or Methods section.

n/a	Confirmed	
☐	☒	The exact sample size (*n*) for each experimental group/condition, given as a discrete number and unit of measurement
☐	☒	A statement on whether measurements were taken from distinct samples or whether the same sample was measured repeatedly
☐	☒	The statistical test(s) used AND whether they are one- or two-sided<br>*Only common tests should be described solely by name; describe more complex techniques in the Methods section.*
☐	☒	A description of all covariates tested
☐	☒	A description of any assumptions or corrections, such as tests of normality and adjustment for multiple comparisons
☐	☒	A full description of the statistical parameters including central tendency (e.g. means) or other basic estimates (e.g. regression coefficient) AND variation (e.g. standard deviation) or associated estimates of uncertainty (e.g. confidence intervals)
☐	☒	For null hypothesis testing, the test statistic (e.g. *F*, *t*, *r*) with confidence intervals, effect sizes, degrees of freedom and *P* value noted<br>*Give P values as exact values whenever suitable.*
☒	☐	For Bayesian analysis, information on the choice of priors and Markov chain Monte Carlo settings
☒	☐	For hierarchical and complex designs, identification of the appropriate level for tests and full reporting of outcomes
☒	☐	Estimates of effect sizes (e.g. Cohen's *d*, Pearson's *r*), indicating how they were calculated

Our web collection on statistics for biologists contains articles on many of the points above.

Software and code

Policy information about availability of computer code

Data collection	No software was used.
Data analysis	SkanIt version 6.0.2 was used to analyze the data in cell proliferation assay. STAR version 2.7.8a and StrandNGS version 4.0 were used to analyze the data of RNA sequence. R Studio version 1.0153 and R version 3.6.0. were used to analyze the data in retrospective analysis of clinical study.

For manuscripts utilizing custom algorithms or software that are central to the research but not yet described in published literature, software must be made available to editors and reviewers. We strongly encourage code deposition in a community repository (e.g. GitHub). See the Nature Portfolio guidelines for submitting code & software for further information.

Data

Policy information about availability of data

All manuscripts must include a data availability statement. This statement should provide the following information, where applicable:

- Accession codes, unique identifiers, or web links for publicly available datasets
- A description of any restrictions on data availability
- For clinical datasets or third party data, please ensure that the statement adheres to our policy

The authors declare that the data that support the findings of this study are available within the article and its supplementary materials.

Human research participants

Policy information about studies involving human research participants and Sex and Gender in Research.

Reporting on sex and gender	Sex and gender were determined based on their self-reporting. No sex- and gender-based analysis were performed in this clinical study. All patients provided written informed consent prior to any study-related procedures. The study protocol was reviewed by the institutional review board from the perspective of ethical, scientific, and medical validity in Chugai Pharmaceutical Co., Ltd.
Population characteristics	All patients were aged 20 years or older, with histologically or cytologically confirmed stage IIIB, stage IV, or postoperative recurrent non-small-cell lung cancer that was confirmed to be ALK-positive by immunohistochemistry and fluorescence in-situ hybridisation (FISH), or RT-PCR using tissue or cell samples.
Recruitment	All patients who were ALK inhibitor-naïve and chemotherapy-naïve or those who had received one prior chemotherapy regimen were enrolled with inclusion criteria (aged ≥20 years, Major organ function meeting the following criteria within 14 days before enrollment, etc) and exclusion criteria (pleural effusion, ascites, pericardial effusion requiring drainage, or meningeal metastases or brain metastases that are symptomatic or require treatment, etc).
Ethics oversight	Institutional review board at Chugai Pharmaceutical Co., Ltd.

Note that full information on the approval of the study protocol must also be provided in the manuscript.

Field-specific reporting

Please select the one below that is the best fit for your research. If you are not sure, read the appropriate sections before making your selection.

☒ Life sciences ☐ Behavioural & social sciences ☐ Ecological, evolutionary & environmental sciences

For a reference copy of the document with all sections, see nature.com/documents/nr-reporting-summary-flat.pdf

Life sciences study design

All studies must disclose on these points even when the disclosure is negative.

Sample size	Sample size of at least three or six was used in most in vitro or in vivo experimental research for statistical analysis. Sample size of 69 was used in this clinical study of a multivariate Cox proportional hazard model.
Data exclusions	No data was excluded in experimental research in this study. In this clinical analysis, we obtained 80 tumor samples. However, after RNA sequencing analysis, we excluded six samples because of insufficient amount of the library. Then, we additionally excluded five samples because the mapping rate was less than 40%.
Replication	Each experiment was independently performed at least twice.
Randomization	No randomization was used in in vitro experiments and in this clinical study. In in vivo experiments, the mice were randomized once their mean of tumor volume.
Blinding	All experiments and this clinical study were not performed blind.

Reporting for specific materials, systems and methods

We require information from authors about some types of materials, experimental systems and methods used in many studies. Here, indicate whether each material, system or method listed is relevant to your study. If you are not sure if a list item applies to your research, read the appropriate section before selecting a response.

Materials & experimental systems

n/a	Involved in the study	
☐	☒	Antibodies
☐	☒	Eukaryotic cell lines
☒	☐	Palaeontology and archaeology
☐	☒	Animals and other organisms
☐	☒	Clinical data
☒	☐	Dual use research of concern

Methods

n/a	Involved in the study	
☒	☐	ChIP-seq
☒	☐	Flow cytometry
☒	☐	MRI-based neuroimaging

Antibodies

Antibodies used	ALK (#3633), phospho-ALK (#3341), FGFR1 (#9740), FGFR2 (#23328), FGFR3 (#4574), FGFR4 (#2894), ERK (#9102), phospho-ERK (#4377), AKT (#4691), phospho-AKT (#4058), EGFR (#4267), phospho-EGFR (#3777), MET (#4560), β-actin (#4970), phospho-MET (#3077), HER2 (#3250), CD44 (#37259), CD133 (#86781), BIM (#2933), VIM (#5741), CDH1 (#3195), S6 (#2217), pS6 (#5364) and STAT3 (#4904), phospho-STAT3 (#9145) (Cell Signaling Technology)<br>cleaved PARP (#ab32064), phospho-HER2 (#ab47262), FGF2 (#ab208687), and FGF12 (#ab231956) (Abcam)
Validation	All antibodies are commercially available and have been validated by the suppliers.

Eukaryotic cell lines

Policy information about cell lines and Sex and Gender in Research

Cell line source(s)	NCI-H2228, NCI-H1650, HCC827, NCI-H1975, HCC2935, HCC1569, AU565, SK-BR-3, RPMI-7951, SK-MEL-3 (ATCC: American Type Culture Collection). IGR-39 (DSMZ: DSMZ-German Collection of Microorganisms and Cell Cultures). COLO 679 (ECACC: European Collection of Authenticated Cell Cultures). A2058 (JCRB: Japanese Collection of Research Bioresources). SNU-2535 (KCLB: Korean Cell Line Bank). II-18 (RIKEN: RIKEN BioResource Research Center).
Authentication	All cell lines were purchased new from the supplier and authenticated by the supplier before purchase.
Mycoplasma contamination	All cell lines were not detected mycoplasma by PCR based assay.
Commonly misidentified lines<br>(See ICLAC register)	Commonly misidentified lines were not used in the study.

Animals and other research organisms

Policy information about studies involving animals; ARRIVE guidelines recommended for reporting animal research, and Sex and Gender in Research

Laboratory animals	Male five-week-old SCID mice (C.B-17/Icr-scid/scidJcl) were obtained from CLEA Japan. Male 4–5-week-old BALB/c-nu/nu mice (CAnN Cg-Foxn1<nu>/CrlCrlj nu/nu) were purchased from Charles River Laboratories Japan.
Wild animals	This study did not include wild animals.
Reporting on sex	Male five-week-old SCID mice and 4–5-week-old BALB/c-nu/nu mice were used.
Field-collected samples	This study did not involve samples collected from the field.
Ethics oversight	In vivo experimental protocols were approved by the institutional review board of Chugai Pharmaceutical Co., Ltd (approval no. 22-015).

Note that full information on the approval of the study protocol must also be provided in the manuscript.

Clinical data

Policy information about clinical studies

All manuscripts should comply with the ICMJE guidelines for publication of clinical research and a completed CONSORT checklist must be included with all submissions.

Clinical trial registration	JapicCTI-132316; JO28928
Study protocol	Qualified researchers may request access to the data including study protocol through the clinical study data request platform (https://www.clinicalstudydatarequest.com/Default.aspx). For further details on Chugai's Data Sharing Policy and how to request access to related clinical study documents, see here (www.chugai-pharm.co.jp/english/profile/rd/ctds_request.html).
Data collection	The clinical data was collected from November 2013 to June 2018 by Chugai Pharmaceutical Co., Ltd.
Outcomes	To determine whether the patients with high FGFR1 or FGF2 expression have shorter PFS of ALK TKIs than those who with low expression, a multivariate Cox proportional hazard model was used to estimate the adjusted hazard ratio (HR) and 95% confidence bands of PFS associated with the level of either FGFR1 or FGF2 mRNA expression in each subset with various patterns of categorical values for these expressions.
